# The association between e-cigarette use and dyslipidemia: A systematic review and meta-analysis of observational studies

**DOI:** 10.1371/journal.pone.0361200

**Published:** 2026-10-26

**Authors:** Humam Emad Rajha, Sara Mohsen, Hadeel Alkuwari, Janatul Naeim, Wejdan Al-Marri, Ahmad Hamdan, Tawanda Chivese, Rafif Mahmood Al Saady

**Affiliations:** 1 College of Medicine, QU Health, Qatar University, Doha, Qatar; 2 Department of Science and Mathematics, School of Interdisciplinary Arts and Sciences, University of Washington, Tacoma, Washington, United States of America; National Institutes of Health, University of the Philippines Manila / De La Salle University, PHILIPPINES

## Abstract

**Background:**

E-cigarette use, also known as vaping, has rapidly increased in recent years, often marketed as a safer alternative to traditional smoking. While the cardiovascular risks of vaping are increasingly being recognized, its relationship with dyslipidemia remains unclear. This systematic review and meta-analysis evaluated the association between e-cigarette use and serum lipid levels, specifically total cholesterol (TC), high-density lipoprotein cholesterol (HDL), low-density lipoprotein cholesterol (LDL), and triglycerides (TG).

**Methods:**

A search of PubMed, Scopus, EMBASE, CENTRAL, CINAHL, medRxiv, and Google Scholar was conducted up to May 31,2025. Eligible studies included adult populations and assessed the association between exclusive e-cigarette use and lipids. Study quality was assessed using the Methodological Standards for Epidemiological Research (MASTER) scale. Meta-analysis of adjusted odds ratios (aOR) for dyslipidemia and unadjusted weighted mean differences (uWMD) for each lipid was conducted using a bias-adjusted inverse-variance heterogeneity method. Heterogeneity was assessed using the I^2^ statistic, and publication bias using Doi plots and the LFK index, with the LFK index used as the primary quantitative criterion. Planned subgroup analyses by age group and sex could not be performed due to insufficient stratified reporting in included studies. The overall quality of evidence for each lipid outcome was evaluated using the Grading of Recommendations Assessment, Development and Evaluation (GRADE) framework,

**Results:**

Of 4,160 identified data sources, seven observational studies from the U.S.A., South Korea, Romania, and Iraq with a total of 25,690 participants met the inclusion criteria. Meta-analysis showed that vaping was associated with higher odds of hypertriglyceridemia (aOR 3.10, 95% CI 1.18 to 8.16, I^2^ = 80.1%, LFK = 1.34), and higher odds of low HDL (aOR 3.26, 95% CI 2.28 to 4.65, I^2^ = 3.1%, LFK = 1.41). In studies with unadjusted mean lipid levels, the primary quality-effects model showed no statistically significant difference in TC (unadjusted weighted mean difference (uWMD) +3.66 mg/dL, 95% CI -13.89 to 21.20, I^2^ = 86.9%, LFK = 9.44) but showed higher LDL (uWMD +12.40 mg/dL, 95% CI 7.91 to 16.88, I^2^ = 29.1%, LFK = 1.05), and VLDL (uWMD +3.23 mg/dL, 95% CI 1.43 to 5.02, I^2^ = 0.0%, publication bias not assessed). For all outcomes, the GRADE certainty of evidence was very low.

**Conclusion:**

This synthesis suggests that e-cigarette use may be associated with adverse lipid profiles, but the evidence remains observational and of very low certainty, limiting causal inference. Further well-designed longitudinal studies, using validated smoking-status assessment, detailed vaping exposure measures, fasting lipid panels, repeated lipid measurements, and adjustment for key cardiometabolic confounders, are needed to clarify these associations.

## 1. Introduction

Tobacco smoking remains one of the biggest global public health problems of the 21^st^ century, because of its high prevalence and strong association with morbidity and mortality [1,2]. As of June 2025, over a fifth of the global adult population, an estimated 1.3 billion individuals, were tobacco users, and 80% of these were people living in low- and middle-income countries (LMICs), where health systems are struggling to cope with multiple burdens of infectious diseases, violence, over-and under-nutrition, and the escalating non-communicable disease pandemic [3]. Tobacco smoking affects almost every body system [4] and is attributable for up to 90% of lung cancer and chronic obstructive pulmonary disease, 25% of cardiovascular disease, and contributes to increased risk of most cancers, other chronic diseases, infertility, and pregnancy complications [5]. Tobacco kills around half of its users and is responsible for an estimated seven million deaths annually, again with the majority of deaths occurring in LMICs [6]. In recognition of the global public health impact of tobacco smoking, many interventions have been implemented to control tobacco use, and these include: policies on plain packaging, health warnings on tobacco packaging, sin taxes, limiting tobacco advertising, putting age limits on tobacco consumption, and widespread use of tobacco cessation programs [6].

Introduced into the U.S. market in 2007, electronic nicotine delivery systems (ENDS), known as e-cigarettes or vaping, were initially marketed as a tobacco cessation tool that delivered smokeless nicotine, and therefore helped wean tobacco smokers from cigarette smoking by replacing the nicotine in tobacco smoke [7]. However, the rapid uptake of vaping by youths [8] and the resultant spate of many unexplained cases of vaping-related lung injury brought about a paradigm shift from vaping as a tobacco smoking cessation strategy to vaping as a public health threat [9]. Despite claims of their relative safety, e-cigarettes have been associated with a range of health risks, particularly concerning lung and cardiovascular health [10–13]. A recent systematic review and meta-analysis reported a 33% higher risk of myocardial infarction among e-cigarette users compared to non-users [12]. Associations between vaping and other cardiovascular diseases have also been described [14]. The mechanisms by which vaping increases the risk of CVD remain understudied, and some of the suggested mechanisms include the direct effects of nicotine on heart rate and blood pressure, impairment of endothelial cells, oxidative stress from reactive oxygen species from chemicals in vaping liquids, inflammation and, possibly, vaping-related dyslipidemia [14].

Dyslipidemia, a major risk factor for CVD, is known to be exacerbated by tobacco use [15,16]. Dyslipidemia involves imbalances in lipid levels, particularly low-density lipoprotein (LDL), high-density lipoprotein (HDL), and triglycerides (TG). Understanding this relationship is vital because even marginal, sustained perturbations in lipid profiles, such as elevated LDL or depressed HDL, can accelerate the pathogenesis of atherosclerotic cardiovascular disease (ASCVD), potentially translating into long-term cardiovascular risk even among younger cohorts [17]. Nicotine, a primary constituent of traditional cigarettes, has been implicated in this association. It is believed to induce changes in adipocyte function through adenosine monophosphate-activated protein kinase (AMPK) activation and catecholamine release, resulting in increased lipolysis and subsequent elevations in LDL levels, reductions in HDL levels, and the development of dyslipidemia [18,19]. Although various cardiovascular outcomes linked to e-cigarette use have been investigated, the relationship between e-cigarettes and dyslipidemia remains underexplored. Recent primary studies examining this association have yielded inconsistent results [20,21]. To reduce confounding from concurrent combustible tobacco use, this review focused on “exclusive vaping,” defined here as e-cigarette use without concurrent use of combustible tobacco products. However, past combustible tobacco use may still confound observed lipid associations and was therefore considered in the interpretation of the findings. This systematic review and meta-analysis aimed to clarify these inconsistencies by synthesizing the evidence from existing research on the association between e-cigarette use and dyslipidemia. Specific objectives of the meta-analysis were to compare levels of total cholesterol (TC), high-density lipoprotein cholesterol (HDL), low-density lipoprotein cholesterol (LDL), very low-density lipoprotein cholesterol (VLDL) and triglycerides (TG).

## 2. Methods

### 2.1. Study design

This systematic review and meta-analysis was carried out and reported in accordance with the Preferred Reporting Items for Systematic Reviews and Meta-Analyses (PRISMA) 2020 Guidelines [22]. The PRISMA checklist for reporting this systematic review and meta-analysis is found in Supplementary document 1. The protocol of the study was registered in the International Prospective Register of Systematic Reviews (PROSPERO) (PROSPERO ID: CRD420251022048). No major deviations from the primary protocol were made.

### 2.2. Data sources & search terms

A comprehensive search strategy was conducted on November 26, 2024, and last updated on May 31, 2025. We searched the Cochrane Central Register of Controlled Trials (CENTRAL), PubMed, Scopus, Cumulative Index to Nursing and Allied Health Literature (CINAHL), Google Scholar, and EMBASE for peer-reviewed publications. The primary search terms and keywords used were focused on vaping and dyslipidemia, guided by previously conducted systematic reviews and the full search is shown in Supplementary document 2. No restrictions were applied on language or publication date. Additionally, we screened reference lists of included articles, as well as relevant reviews and meta-analyses, to identify further eligible studies. A preprint search was also conducted on medRxiv.

### 2.3. Screening of studies

All search results were exported to EndNote (Version 21, Clarivate Analytics) [23], where duplicates were removed, and then exported to Rayyan systematic review management software [24] for title and abstract screening. Six authors (HR, SM, AH, HA, WA, JN) performed the screening in three independent paired teams. After the title and abstract screening, studies preliminarily selected during this stage were retrieved for the full-text screening stage. Full-text screening was then conducted independently by the same six authors, who were assigned to three different paired teams to minimize bias. Each pair conducted a blinded full-text screening. Any conflicts arising during the screening stages were resolved by a third reviewer. Primary reasons for exclusion of studies in the full-text screening were recorded in Microsoft Excel.

### 2.4. Eligibility criteria

We included observational studies, including cohort, case-control, and cross-sectional studies that included adult populations and assessed the association between e-cigarette use and lipids: TC, HDL, LDL, VLDL, TG. Studies were eligible if they compared exclusive e-cigarette users, defined as current e-cigarette users without concurrent tobacco smoking, regardless of their past smoking history, with non-current e-cigarette users or non-smokers. Only biochemically validated measures of dyslipidemia were accepted. Studies of children under 18 or participants who used both e-cigarettes and tobacco cigarettes simultaneously were excluded. Reviews, editorials, conference abstracts, and animal studies were also excluded.

### 2.5. Outcomes

The primary outcome was dyslipidemia, defined in accordance with thresholds from The National Cholesterol Education Program (NCEP) Adult Treatment Panel III (ATP III) criteria as any of the following; TC ≥ 200 mg/dL (≥5.2 mmol/L), LDL‑C ≥ 130 mg/dL (≥3.4 mmol/L), HDL‑C < 40 mg/dL (<1.0 mmol/L) in men or <50 mg/dL (<1.3 mmol/L) in women, and TG ≥ 150 mg/dL (≥1.7 mmol/L) [25,26]. Secondary outcomes were mean blood levels of each lipid marker.

### 2.6. Data extraction

Data were extracted using a standardised Microsoft Excel spreadsheet. The extraction form was not formally pilot-tested on a subset of studies because only seven studies met the inclusion criteria; instead, the form was developed to capture all prespecified study, participant, exposure, comparator, and lipid-outcome variables and was applied uniformly to all included studies. Six authors (HR, SM, AH, HA, WA, JN) worked in three pairs, and within each pair, reviewers extracted all data fields independently. Formal inter-rater reliability or inter-extractor agreement statistics were not calculated before full extraction. Discrepancies were resolved through discussion or adjudication by a third reviewer. Extracted data included study characteristics, participant demographics, exposure definitions, comparator definitions, covariates, and lipid outcome data.

For lipid outcomes, we extracted two complementary data types to support both primary and secondary analyses. Adjusted odds ratios (aOR) and their 95% confidence intervals (95% CI) were prioritized for the primary meta-analysis when reported. Raw continuous mean lipid levels and standard deviations were extracted from all eligible studies whenever available, regardless of whether adjusted effect estimates were reported, to enable secondary analyses of pooled mean concentrations. Studies that reported both data types contributed to both analyses. When raw values were not reported in the main text, we reviewed tables, figures, appendices, and supplementary materials.

### 2.7. Quality assessment & certainty

The quality of the included articles was assessed using the Methodological Standard for Epidemiological Research (MASTER) scale [27]. This tool was selected over other common scales as it is specifically tailored so that it can be used to assess different observational epidemiological research using the same standards. The MASTER scale also provides a detailed numerical assessment across multiple domains, evaluating not only bias but also the rigour of design, analysis, and interpretation.

The MASTER scale comprises seven core domains, subdivided into 36 safeguards, which were used to evaluate the internal validity of the included comparative studies. These domains assess the equality of key methodological processes: [1] Equal Recruitment [2], Equal Retention [3], Equal Ascertainment [4], Equal Implementation [5], Equal Prognosis [6], Sufficient Analysis, and [7] Temporal Precedence.

The studies were assigned to three reviewer pairs. Within each pair, reviewers independently assessed the studies, compared their assessments, and resolved disagreements through discussion or consultation with a third senior researcher. Any disagreements between the pairs were resolved through consultation with a third senior researcher. The results of the quality assessment were incorporated into the review’s interpretation. Studies were critically interpreted with consideration of their methodological strengths and limitations.

### 2.8. Data synthesis

Statistical analyses were performed in Stata version 18 (College Station, TX, USA) using the *metan* package. For each lipid parameter (TC, HDL, LDL, VLDL, and TG), we conducted meta-analyses using a hierarchical approach to effect estimates: adjusted effect measures (aOR, and their 95% CIs) were prioritized when available; if adjusted estimates were absent, unadjusted effect measures were extracted and pooled; when neither adjusted nor unadjusted effect estimates were reported, raw continuous data (mean lipid levels and standard deviations) were used to compute weighted mean differences (WMDs). This hierarchy ensured maximal inclusion of eligible effect sizes while prioritising estimates least susceptible to confounding.

The primary analysis employed the bias-adjusted inverse-variance heterogeneity (quality-effects) model, which incorporates study-specific quality scores derived from the MASTER scale to adjust weighting and account for methodological variability across studies. A DerSimonian–Laird random-effects model was also fitted as a sensitivity analysis and to facilitate comparison with other meta-analyses.

Heterogeneity was quantified using the *I*² statistic, Cochran’s *Q* test (*P* < 0.10 indicating significance), and τ². *I*² values of 25%, 50%, and 75% were interpreted as low, moderate, and high inconsistency, respectively. Pre-specified subgroup analyses evaluated effect modification by age (<40 vs. ≥ 40 years), sex (male vs. female), and population type (general vs. clinical cohorts).

Leave-one-out sensitivity analyses were performed systematically for all lipid outcomes by iteratively removing each study to assess its influence on the pooled estimate and heterogeneity [28]. These analyses were conducted post-hoc to explore drivers of heterogeneity after initial meta-analysis. A study was considered disproportionately influential if its removal reduced *I*² by ≥20 percentage points or narrowed the 95% confidence interval width by ≥30%. All studies underwent this assessment; only those meeting the influence criterion were highlighted in the results narrative.

Publication bias was assessed using complementary visual and quantitative methods: [1] visual inspection of Doi plots, and [2] quantitative assessment via the LFK index (asymmetry >2 or <−2 interpreted as major bias) [29]. The LFK index served as the primary basis for inference regarding bias presence; when visual and quantitative assessments diverged, the quantitative LFK result was prioritized to minimize subjectivity. Findings from both tools are reported to enhance transparency. The overall quality of evidence for each lipid outcome was evaluated using the Grading of Recommendations Assessment, Development and Evaluation (GRADE) framework.

### 2.9. Ethics and patient and public involvement

This systematic review utilized published data; therefore, there was no need for ethical approval. There was no patient or public involvement in the design and conduct of this study.

## 3. Results

### 3.1. Search results (PRISMA)

The database search identified 4,160 potentially relevant study reports. After removing 1,106 duplicates, we screened 3,054 unique records by title and abstract and excluded 3,009 that did not meet the inclusion criteria. Full-text review was performed for the remaining 45 articles, and 38 were excluded because they did not assess dyslipidemia (n = 6), did not examine vaping (n = 6), were reviews, editorials, case reports, conference abstracts (n = 20), did not compare vaping to no vaping (n = 3), or had no full text available (n = 3) (Fig 1). Seven studies [20,30–35] that met eligibility criteria were finally included.

**Fig 1:**
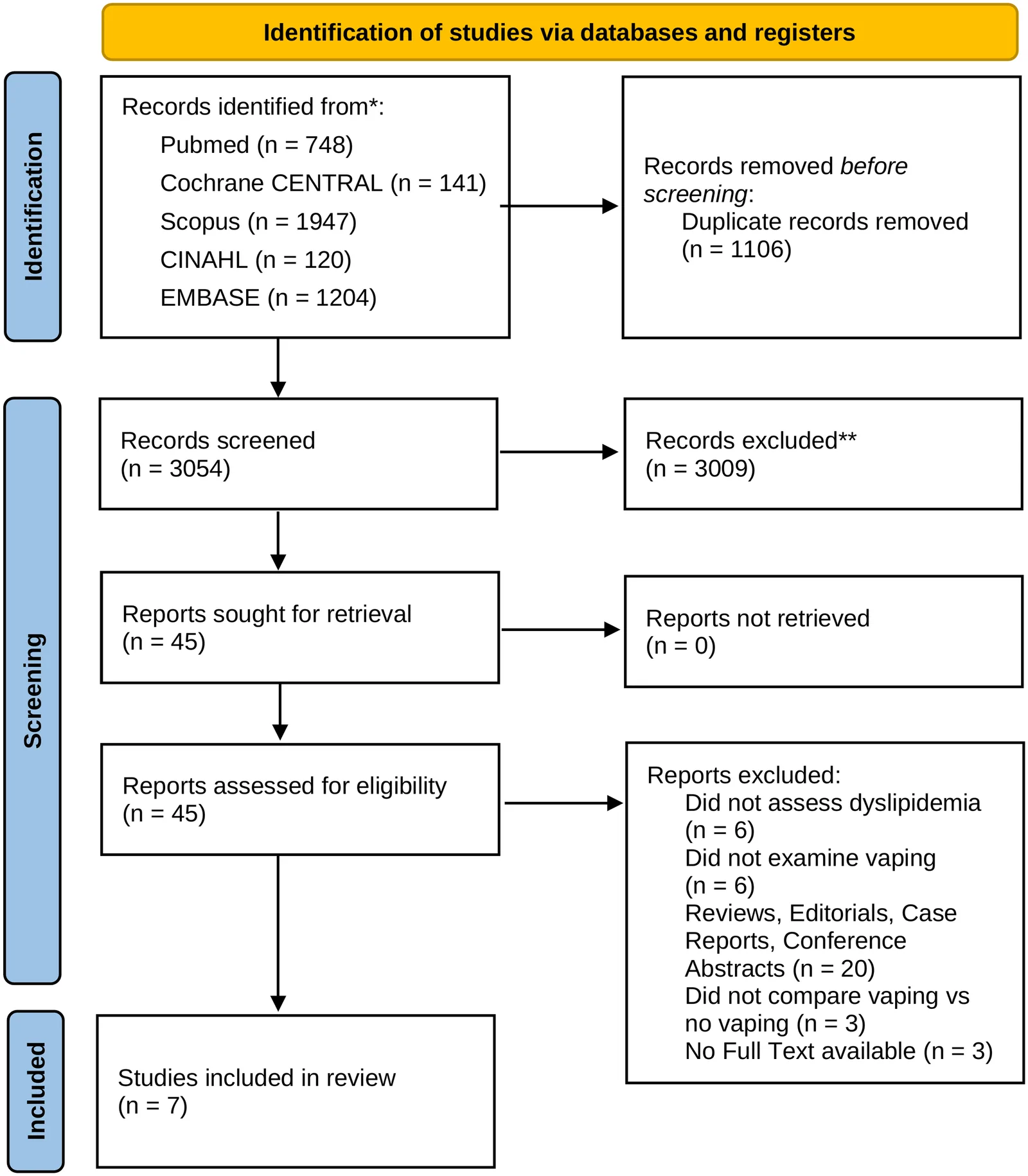
The PRISMA flow chart.

### 3.2. Characteristics of included studies

The seven included studies were published between 2019 and 2024; six were cross-sectional studies [20,30,31,33–35], and the remaining study was a case-control study [36]. The studies were conducted in the United States (n = 3) [20,33,34], South Korea (n = 2) [31,35], Romania (n = 1) [30], and Iraq (n = 1) [36]. The total sample size of all studies was 25,690 participants and ranged from 42 to 14,738 participants within studies [31,33]. The mean age of participants across studies generally reflected young adult populations (23–46.6 years), with the oldest cohort reported in Kim (2020) [31]. Body mass index (BMI) data were available in five of the seven studies, with mean values ranging from 23.2 kg/m^2^ in Matheson (2024) to 28.3 kg/m^2^ in Okafor (2022) [33,34]. Most studies had a smaller vaping group than the control groups, which primarily consisted of never smokers or never e-cigarette users. Further details are shown in Table 1 and in Supplementary Table S1 in S2 File.

**Table 1:** Characteristics of Included Studies.

Author, Year	Study Design	Study Duration	Country	Males No. (%)	Mean Age (±SD)	Mean BMI (±SD)	Vaping Sample Size	Control Type	Control Sample Size
Hasan, 2024 [36]	Case-control	2023-2024	Iraq	150 (100%)	24 (1)	Not reported	100	Non-smokers	50
Lim, 2024 [35]	Cross-sectional	2019-2020	Korea	2094 (47%)	42 (0.5)	24 (0.2)	296	Non-smokers	4,108
Matheson, 2024 [33]	Cross-sectional	Not reported	US	19 (45.2%)	23 (3)	23.2 (4)	21	Non-smokers	21
Okafor, 2022 [34]	Cross-sectional	2015-2018	US	4205 (59.7%)	29.9 (1.41)	28.3 (0.8)	187	Non-smokers	6,862
Majid, 2021 [20]	Cross-sectional	2014-2020	US	95 (56.2%)	28 (6)	Not reported	65	Non-smokers	104
Kim, 2020 [31]	Cross-sectional	2013-2015	Korea	7164 (52.0%)	46.6 (2)	24.1 (2)	325	Non-E-cig users	13,459
Badea, 2019 [30]	Cross-sectional	2017-2019	Romania	18 (19.6%)	28.43 (9.94)	23.48 (3.91)	34	Non-smokers	58

NM; Not Mentioned

### 3.3. Quality assessment

The included studies [20,30,31,33–36] had MASTER scale scores with a mean of 24 (Supplementary Figure S1 and Supplementary Table S2 in S2 File). Most studies demonstrated moderate performance in the equal implementation domain but fell short in the temporal precedence and equal prognosis domains, likely due to their study designs. All studies had low scores in equal recruitment and equal ascertainment due to the lack of randomisation in their study designs.

### 3.4. Association between vaping and levels of TC

Five studies were included in the meta-analysis assessing the association between e-cigarette use and serum total cholesterol (TC) levels (Fig 2). None of the studies reported adjusted effect sizes on the association between vaping and total cholesterol. Results of the unadjusted analyses from all five studies [20,30,33,35,36] showed a tendency toward higher TC levels in vapers, compared to non-vapers, although confidence intervals of three of the studies suggested considerable uncertainty. After meta-analysis, the overall weighted mean difference (WMD) was + 3.66 mg/dL higher in vapers compared to non-vapers (95% CI −13.89 to 21.20). However, the confidence interval crossed the null value (zero), indicating no statistically significant difference. Even if statistically significant, a mean difference of +3.66 mg/dL would likely be of minimal clinical relevance for cardiovascular risk assessment. The analysis also showed high heterogeneity (I² = 86.9%) and major asymmetry on the Doi plot (LFK index = +9.44), suggesting possible publication bias (Supplementary Figure S2 in S2 File). However, with only five studies included, both the magnitude of heterogeneity and the bias assessment should be interpreted cautiously, and potential sources of heterogeneity could not be explored due to insufficient data for meaningful subgroup or meta-regression analyses. Leave-one-out sensitivity analyses showed that heterogeneity decreased and the confidence interval narrowed after excluding Hasan, 2024 [36], the only case–control study and the only study conducted exclusively in Iraqi men (Supplementary Figure S3 in S2 File). Analysis of the certainty of current evidence on the association between vaping and TC was of very low GRADE, downgraded due to low quality of studies, inconsistency (heterogeneity), uncertainty (wide confidence interval which crosses the null), and possible publication bias/small effect size (Supplementary Table S3 in S2 File).

**Fig 2:**
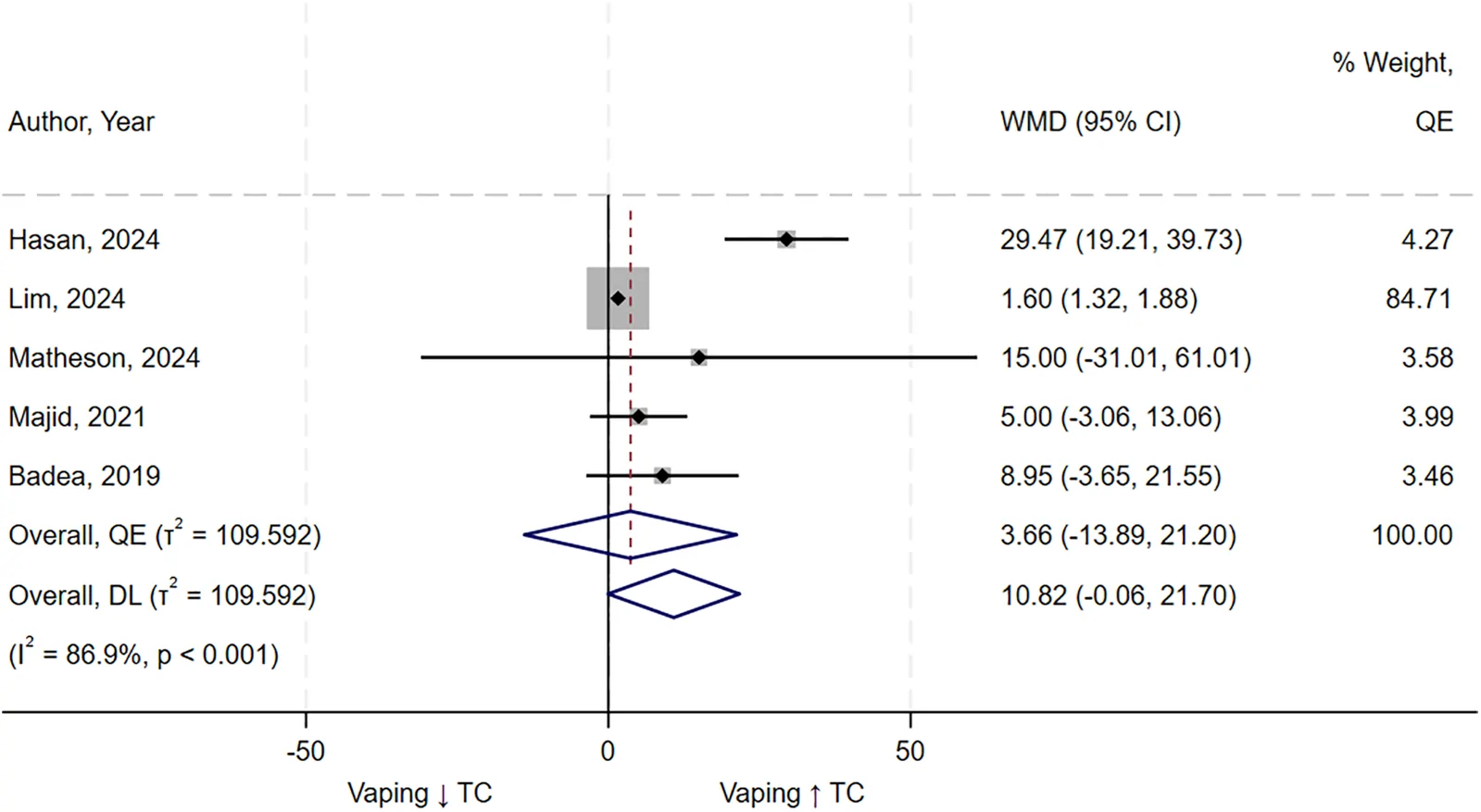
Forest plot showing a comparison of mean TC levels between vapers and non-vapers. Effect sizes are presented as weighted mean differences (WMD) in mg/dL with 95% confidence intervals (CI) using the Doi’s quality effects model (QE) and the random effects model (DL). Squares represent individual study estimates, with size proportional to study weight. The diamond indicates the overall estimate.

### 3.5. Association between vaping and HDL

Seven of the included studies [20,30–36] reported data comparing HDL cholesterol by vaping status. Only *t*wo studies reported adjusted ORs for the relationship between low HDL levels and vaping. Both studies reported odds ratios suggesting higher odds of low HDL levels in vapers [31,37]. The overall adjusted OR was + 3.26 (95% CI 2.28 to 4.65, I² = 3.1%) with minimal heterogeneity among the studies (Fig 3).

**Fig 3:**
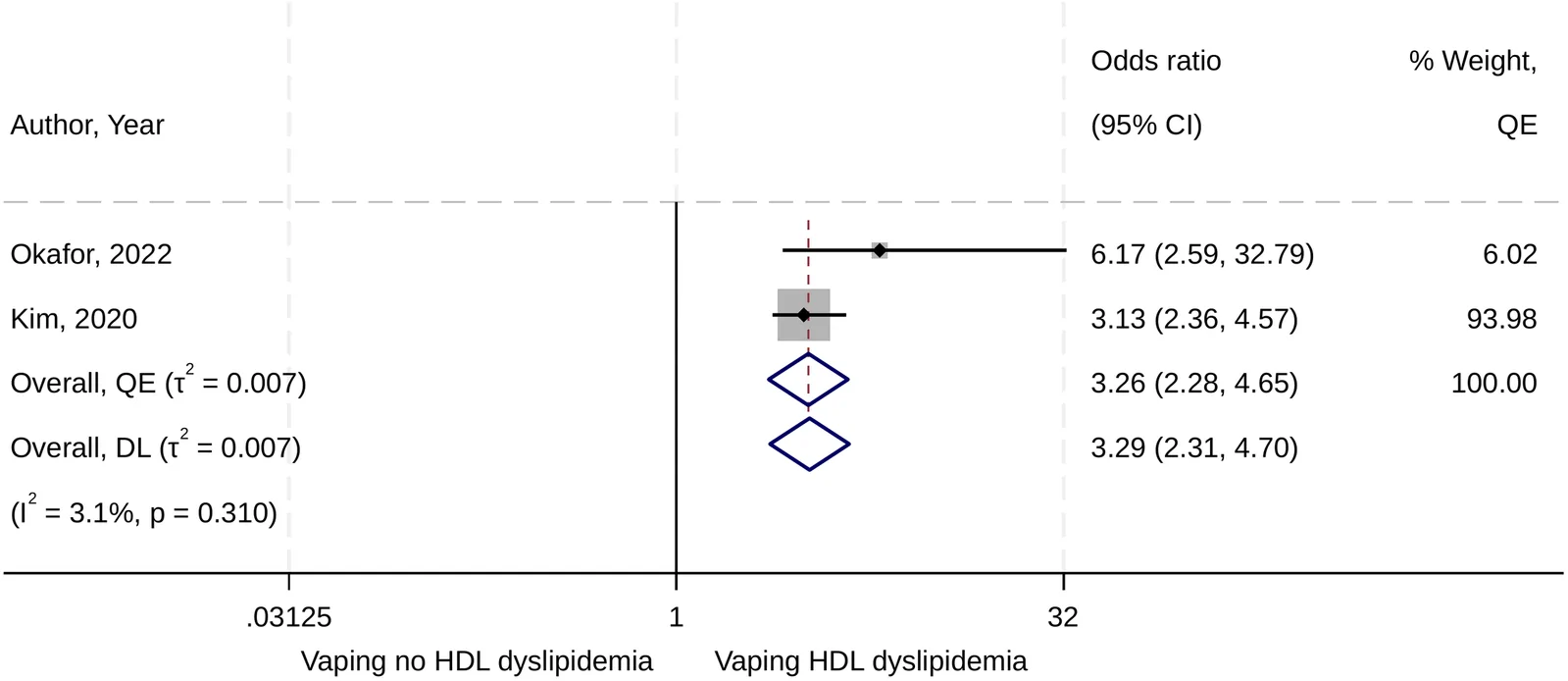
Forest plot of the adjusted association between vaping and HDL dyslipidemia. Effect sizes are presented as adjusted odds ratios (OR) with 95% confidence intervals (CI) using the Doi’s quality effects model and the random effects model. Squares represent individual study estimates, with size proportional to study weight. The diamond indicates the overall estimate. HDL dyslipidemia defined as HDL cholesterol <40 mg/dL (<1.0 mmol/L) in men or <50 mg/dL (<1.3 mmol/L) in women. Okafor, 2022, adjusted for age, sex, race/ethnicity status, education, income, alcohol use, other tobacco product use, physical activity level, and body mass index. Kim, 2020, adjusted for age, sex, cigarette use, alcohol consumption, physical activity, household income, and education level.

Data from six studies reporting unadjusted mean HDL levels showed varied findings by vaping status [20,30,31,33,35,36]. The overall unadjusted WMD suggested an almost one mg/dL decrease in HDL in vapers, compared to non-vapers (uWMD −0.91 mg/dL, 95% CI −1.40, −0.43, I² = 83.0%), with high heterogeneity (Fig 4). The Doi plot showed minor asymmetry, suggesting low concerns about publication bias (Supplementary Figure S4 in S2 File). The GRADE certainty of current evidence for HDL was very low (Supplementary Table S3 in S2 File).

**Fig 4:**
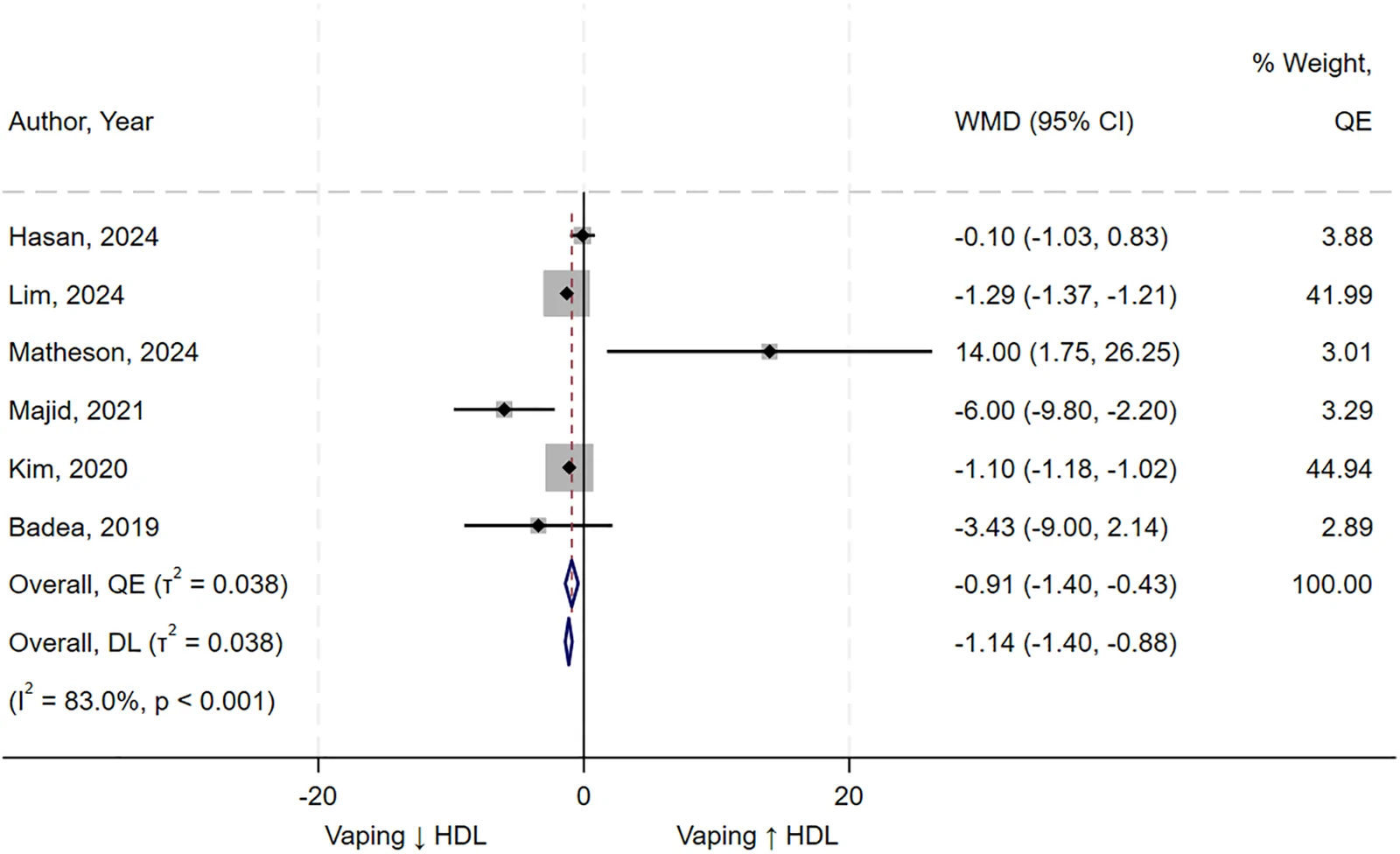
Forest plot showing a comparison of mean HDL levels between vapers and non-vapers. Effect sizes are presented as weighted mean differences (WMD) with 95% confidence intervals (CI) using the Doi’s quality effects model and the random effects model. Squares represent individual study estimates, with size proportional to study weight. The diamond indicates the overall estimate.

### 3.6. Association between vaping and LDL

The effect of e-cigarette use on LDL levels was investigated by four studies (Fig 5). Generally, these studies reported a higher LDL level among e-cigarette users compared to non-users [20,30,33,36]. None of the studies reported adjusted effect sizes for the association. After meta-analysis, the primary quality-effects model showed higher LDL levels among e-cigarette users than non-users (unWMD + 12.40 mg/dL, 95% CI 7.91 to 16.88) and the random-effects sensitivity estimate was similar (uWMD + 11.57 mg/dL, 95% CI 7.64 to 15.51, I² = 29.1%). The Doi plot showed a symmetrical distribution of the study effect sizes, suggesting no major concerns about publication bias (Supplementary Figure S5 in S2 File). Sensitivity analyses showed that the uncertainty significantly declined in the overall estimates in leave-one-out analyses upon leaving out Majid, 2021 [20] (Supplementary Figure S6 in S2 File). The GRADE certainty of current evidence on LDL was very low (Supplementary Table S3 in S2 File).

**Fig 5:**
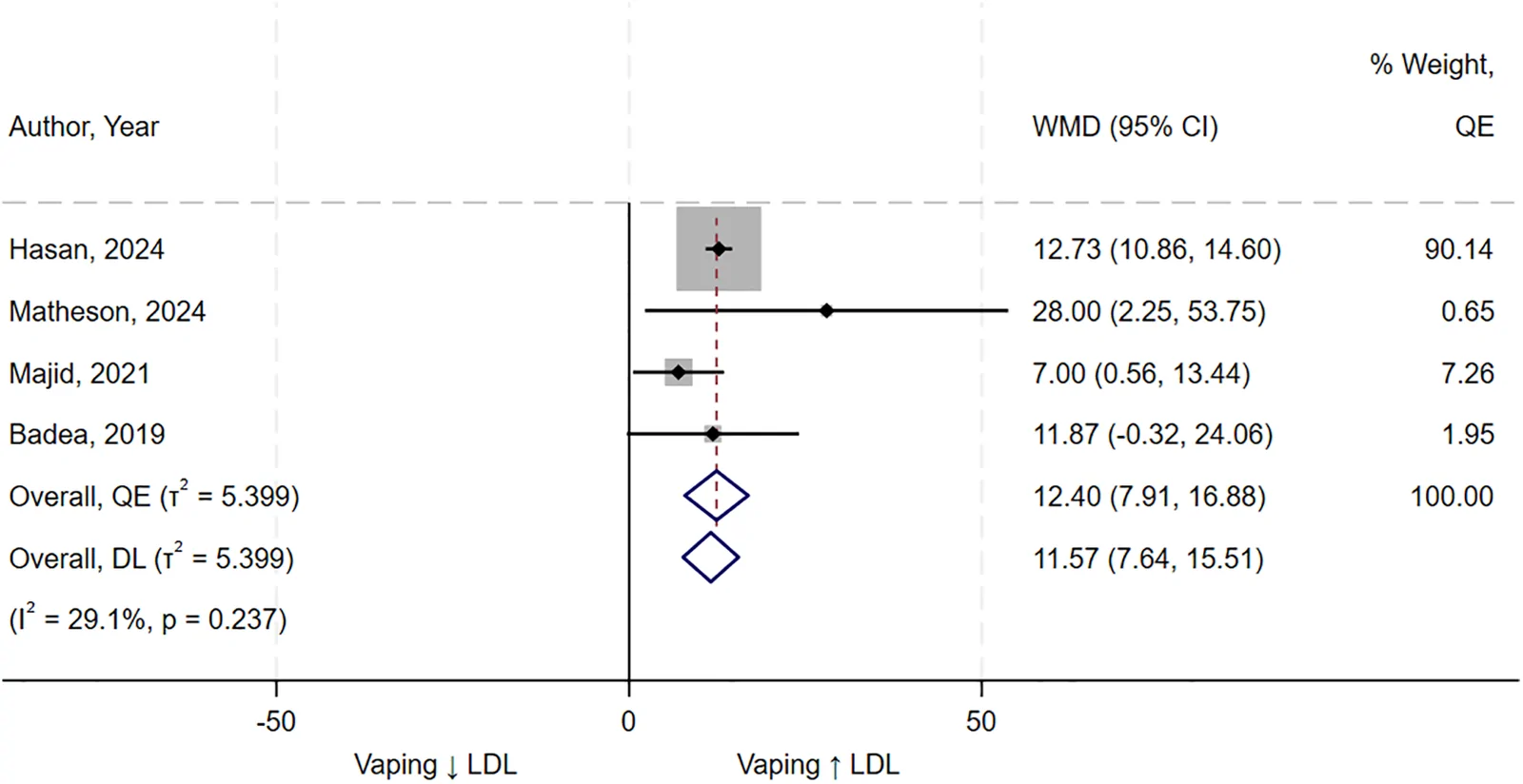
Forest plot showing a comparison of mean LDL levels between vapers and non-vapers. Effect sizes are presented as weighted mean differences (WMD) with 95% confidence intervals (CI) using the Doi’s quality effects model and the random effects model. Squares represent individual study estimates, with size proportional to study weight. The diamond indicates the overall estimate.

### 3.7. Association between vaping and VLDL

Two studies assessed the association between vaping and VLDL levels (Fig 6). Neither study reported adjusted effect sizes [20,30]. Both reported higher VLDL levels among vapers than controls, and the pooled unadjusted WMD was + 3.23 mg/dL (95% CI 1.43 to 5.02, I² = 0%). Assessment of publication bias was not possible because only two studies were included.

**Fig 6:**
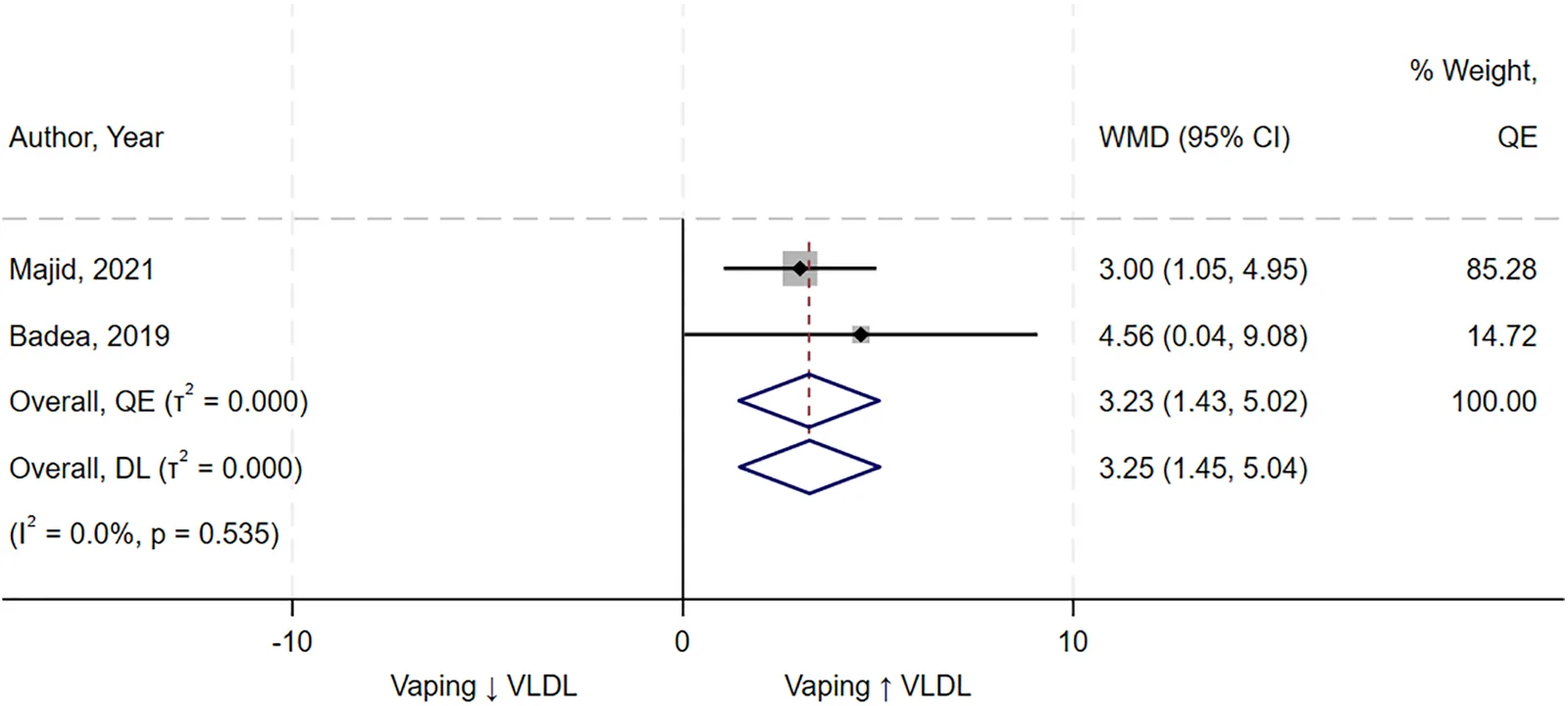
Forest plot showing a comparison of mean VLDL levels between vapers and non-vapers. Effect sizes are presented as weighted mean differences (WMD) with 95% confidence intervals (CI) using the Doi’s quality effects model and the random effects model (DL). Squares represent individual study estimates, with size proportional to study weight. The diamond indicates the overall estimate.

### 3.8. Association between vaping and TG

Two studies reported adjusted ORs for the association between vaping and elevated TG levels, with results from both studies suggesting a harmful effect of vaping on TG [35,37]. The overall adjusted OR was + 3.10 (95% CI 1.18 to 8.16, I² = 80.1%) with high heterogeneity (Fig 7).

**Fig 7:**
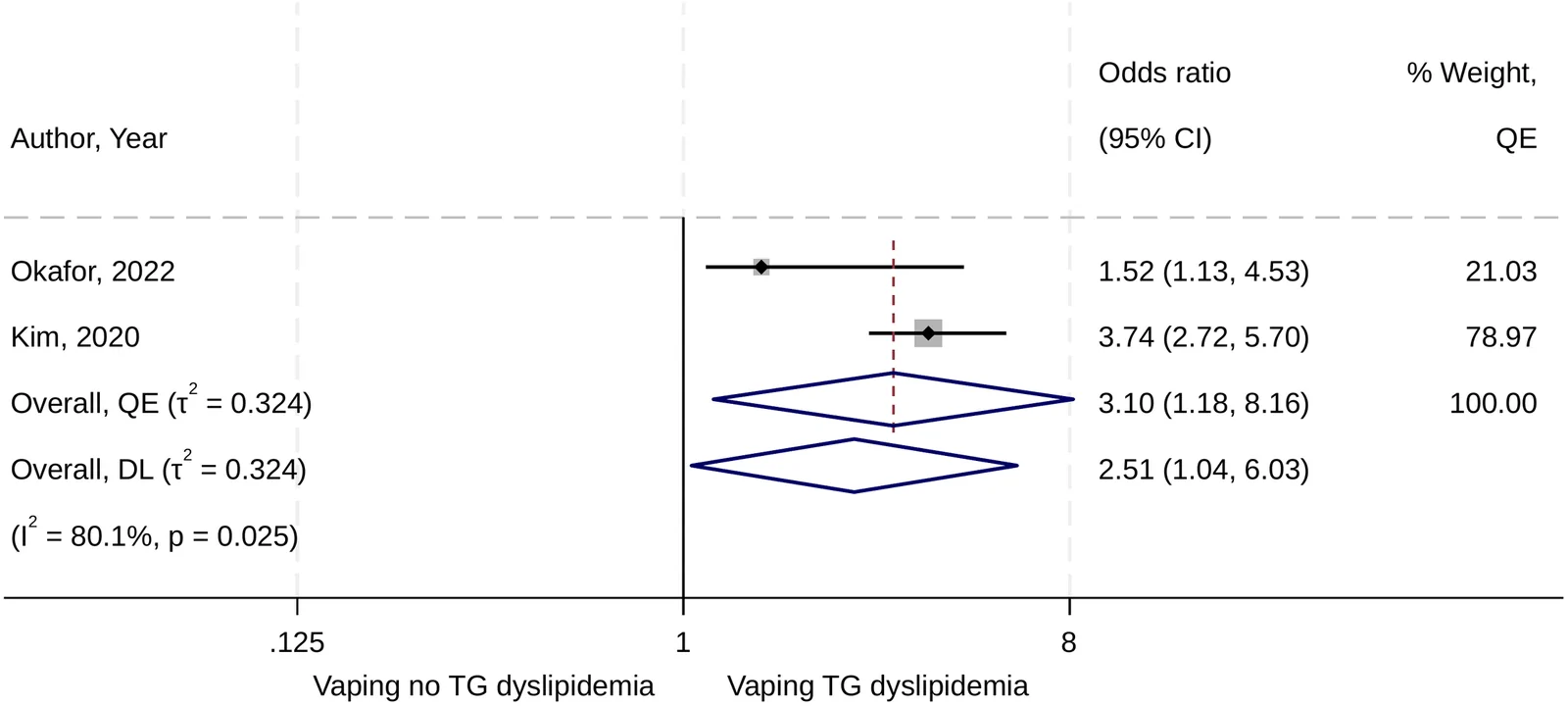
Forest plot of the adjusted association between vaping and hypertriglyceridemia (TG dyslipidemia). Effect sizes are presented as adjusted ORs with 95% confidence intervals (CI) using the Doi’s quality effects model and the random effects model. Squares represent individual study estimates, with size proportional to study weight. The diamond indicates the overall estimate. TG dyslipidemia defined as TG ≥ 150 mg/dL (≥ 1.7 mmol/L). Okafor, 2022, adjusted for age, sex, race/ethnicity status, education, income, alcohol use, other tobacco product use, physical activity level, and body mass index. Kim, 2020 adjusted for age, sex, cigarette use, alcohol consumption, physical activity, household income, and education level.

Data from the six studies reporting unadjusted mean TG levels suggested higher TG levels in vapers than in controls, with marked variability in the magnitude of effect [20,30,31,33,35,36]. The overall pooled unadjusted WMD was + 29.89 mg/dL (95% CI 6.01 to 53.78, I² = 99.7%) with high heterogeneity (Fig 8). The Doi plot showed minor asymmetry, and the LFK index was −1.34, indicating no major asymmetry (Supplementary Figure S7 in S2 File). The GRADE certainty of current evidence on TG was very low (Supplementary Table S3 in S2 File).

**Fig 8:**
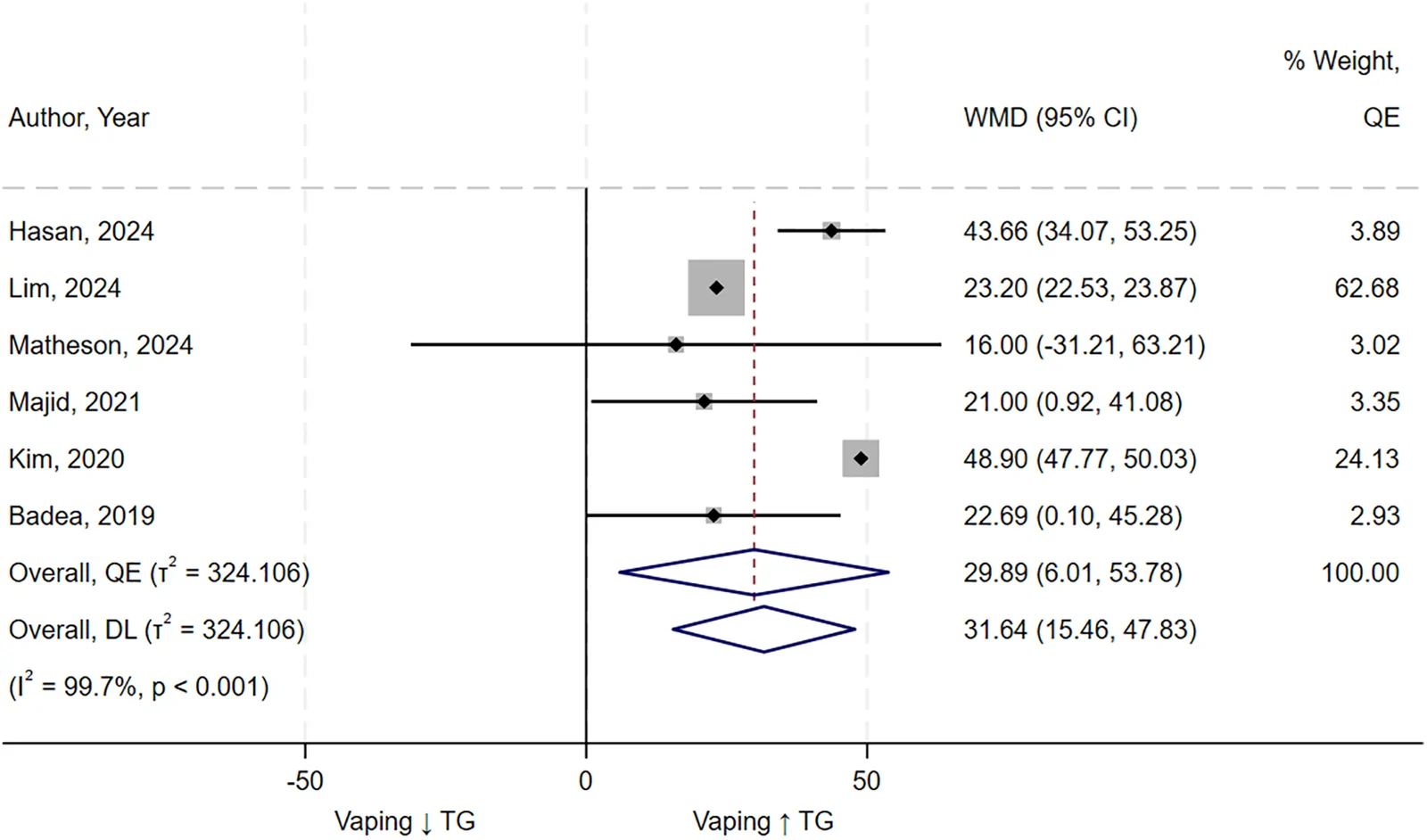
Forest plot showing a comparison of mean TG levels between vapers and non-vapers. Effect sizes are presented as weighted mean differences (WMD) in mg/dL with 95% confidence intervals (CI) using the Doi’s quality effects model and the random effects model. Squares represent individual study estimates, with size proportional to study weight. The diamond indicates the overall estimate.

### 3.9. GRADE certainty of evidence

The certainty of evidence was assessed using the GRADE framework, starting from low certainty for all outcomes due to the observational study designs. Evidence for all outcomes was downgraded to very low certainty because of risk of bias, inconsistency, imprecision, and possible publication bias /small-study effects (Table 2; Supplementary Tables S3 and S4 in S2 File).

**Table 2:** Summary of Findings.

Outcome	Number of studies	Number of participants	Weighted mean difference (WMD) (unadjusted)	Overall odds ratio (OR)	Heterogeneity	Publication bias	GRADE certainty
Total Cholesterol	WMD: 5	4857	+3.66 mg/dL(95% CI −13.89 to 21.20)	–	WMD: *I²* = 86.9%	Doi plot: Major asymmetry LFK Index: 9.44	Very Low ⊕○○○
HDL	WMD: 6 OR: 2	WMD: 18614 OR: 20,833	−0.91 mg/dL(95% CI −1.40 to −0.43)	3.26 (95% CI 2.28 to 4.65)	WMD: *I²* = 83.0% OR: *I²* = 3.1%	Doi plot: Minor asymmetry LFK Index: 1.41	Very Low ⊕○○○
LDL	WMD: 4	453	+12.40 mg/dL(95% CI 7.91 to 16.88)	–	WMD: *I²* = 29.1%	Doi plot: Minor asymmetry LFK Index: 1.05	Very Low ⊕○○○
VLDL	WMD: 2	261	+3.23 mg/dL(95% CI 1.43 to 5.02)	–	WMD: *I²* = 0%	Insufficient studies.	Very Low ⊕○○○
Triglycerides	WMD: 6 OR: 2	WMD: 18614 OR: 20,833	+29.89 mg/dL(95% CI 6.01 to 53.78)	3.10 (95% CI 1.18 to 8.16)	WMD: *I²* = 99.7% OR: *I²* = 80.1%	Doi plot: Minor asymmetry LFK Index: −1.34	Very Low ⊕○○○

## 4. Discussion

This systematic review and meta-analysis identified varying associations between e-cigarette use and serum lipid profiles. Unadjusted analyses suggested higher mean levels of LDL, VLDL, and triglycerides and lower HDL among vapers than in non-vapers; TC showed a non-significant mean difference, and the certainty of evidence was very low. Adjusted analyses, available only for HDL and triglycerides, indicated a stronger independent association with dyslipidemia patterns – specifically lower HDL and higher triglycerides- although with low- certainty evidence.

The findings of this meta-analysis showed a point estimate in the direction of higher TC among e-cigarette users compared with non-users (uWMD + 3.66 mg/dL), but the confidence interval was wide and crossed the null, indicating substantial uncertainty. This finding should therefore be interpreted cautiously, particularly because the TC analysis was based on unadjusted mean differences and heterogeneity was substantial across studies. To our knowledge, there are no currently published meta-analyses which examined the association between vaping and TC. However, studies examining other forms of smoking provide relevant context. For instance, a comprehensive meta-analysis from 1989 found that adult cigarette smokers had significantly higher TC levels compared to non-smokers, with an average increase of 3%, although updated analyses are needed [38]. In one meta-analysis, waterpipe smoking was associated with a + 5.22 mg/dL increase in TC levels [39]. While e-cigarettes share the delivery of nicotine with these traditional products, it is important to note that the exposure profiles differ significantly; specifically, e-cigarettes lack the combustion-generated carbon monoxide and tar found in conventional cigarettes [40]. Nevertheless, the underlying biological mechanisms through which vaping may influence lipid profiles likely overlap regarding nicotine exposure. These include nicotine-induced catecholamine release, which promotes lipolysis and raises blood levels of free fatty acids (FFAs). These FFAs are converted by the liver into triglycerides and VLDL, which, as a result, raise total cholesterol levels [41,42].

For HDL, our meta-analysis of the two studies which reported adjusted ORs showed that vapers had three-fold higher odds of low HDL levels, when compared to non-vapers. In the context of the current literature, the effect of other types of smoking on HDL levels has been explored. For instance, the literature consistently shows that active and passive cigarette smoking lowers HDL levels [43,44], and several meta-analyses report that HDL levels significantly increase following smoking cessation [45,46]. Similarly, waterpipe smoking has been linked to reduced HDL, with one meta-analysis showing a 3.39 mg/dL decrease and another reporting 75% increase in the odds of low HDL among male users compared to non-smokers [39,47]. However, in interpreting these findings for e-cigarettes, the potential for residual confounding by past smoking history is substantial. Many e-cigarette users are former combustible cigarette smokers, and the observed lower HDL may partially reflect the lingering metabolic effects of past smoking rather than vaping alone [48]. Without adjustment for smoking history, it is difficult to disentangle the direct pharmacological effects of e-cigarette aerosols from the residual confounding of prior toxicant exposure in former smokers. Furthermore, “health selection” bias cannot be ruled out, wherein individuals with pre-existing health concerns or dyslipidemia switch from cigarettes to vaping to mitigate harm, thereby clustering higher-risk lipid profiles within the vaping group [49]. Mechanistically, vaping may lower HDL through pathways similar to combustible smoking, such as oxidative stress from reactive oxygen species (ROS), systemic inflammation associated with reduced HDL synthesis, and nicotine-induced endothelial dysfunction [41,42,50].

For LDL, our analysis was based entirely on unadjusted mean differences and showed higher LDL levels among e-cigarette users compared to non-users, with a WMD of +12.40 mg/dL and moderate heterogeneity. Our results correspond with those reported in observational studies exploring cigarette smoking and LDL. For instance, an observational study showed a measurable difference in lipid parameters between cigarette smokers and non-smokers, indicating markedly higher levels of LDL cholesterol in smokers compared to non-smokers [51]. The observed LDL increases are likely linked to nicotine-induced lipolysis and FFA release [19,41]. Because these estimates were not adjusted for smoking history or other cardiovascular risk factors, they should be interpreted cautiously. Without adjustment for smoking history, it is difficult to disentangle the direct pharmacological effects of e-cigarette aerosols from the residual confounding of prior toxicant exposure in former smokers. Overall, further research is warranted to investigate the association between various types of smoking and LDL.

In relation to VLDL, two studies reporting unadjusted means showed higher VLDL levels among e-cigarette users than non-users, with a pooled uWMD of +3.23 mg/dL. Our findings align with limited but growing evidence that suggests vaping may influence VLDL levels. One meta-analysis reported that young smokers aged between 8–19 showed substantially higher serum VLDL cholesterol levels compared to non-smokers. This increase was also associated with increased LDL cholesterol and triglycerides, along with reduced HDL cholesterol, suggesting that cigarette smoking may lead to early lipid profile disturbances [51]. Because only two studies contributed to this analysis and neither provided adjusted estimates, the certainty of evidence remains very low. These increases in VLDL align with the previously described biological mechanisms for other lipoproteins. Nonetheless, more research is required to investigate how e-cigarettes and other forms of smoking may affect VLDL cholesterol levels.

For TG levels, our meta-analysis of the two studies that reported adjusted ORs suggested a potential 2–3 times greater odds of hypertriglyceridemia, while the unadjusted analysis suggested an average increase in TG of around 30 mg/dL in vapers. Overall, our findings align with the available literature discussing the effects of other types of smoking on TG levels. Cigarette smokers were found to have significantly higher TG levels compared to non-smokers, with a mean difference of 0.50 mmol/L (approximately 44 mg/dL) [52]. Waterpipe users were also found to have around 30 mg/dL higher levels of TG compared to non-users [39]. Mechanistically, the nicotine in e-cigarettes can disrupt TG levels through ROS production alongside the stimulation of catecholamine secretion, which promotes the release of TGs from the liver into the bloodstream [19,41].

This study has several key strengths. To the best of our knowledge, this is the first systematic review and meta-analysis to explore the association between e-cigarette use and changes in lipid profiles. The study addresses an important gap in the literature as e-cigarette use is becoming increasingly popular, especially among young adults. Moreover, we examined all major lipid components to provide a comprehensive clinical picture. Rigorous methodologies were employed throughout, including a thorough and up-to-date literature search, exhaustive eligibility criteria, and meticulous data extraction and analysis. We also used the MASTER scale as a comprehensive and validated quality assessment tool to evaluate the methodological quality of the included studies. To ensure reliable estimates, we applied two statistical models: a Quality Effects model, which accounts for study quality, and a Random Effects model as a sensitivity analysis and for comparability with existing and future literature. Additionally, we quantitatively assessed for the presence of publication bias and conducted separate analyses using adjusted odds ratios for several lipid components where available.

Nonetheless, our meta-analysis possesses several limitations. Most of the included studies were cross-sectional, which limits our ability to establish causal relationships between vaping and dyslipidaemia**.** A major limitation is residual confounding by combustible tobacco history. Although the review focused on exclusive current e-cigarette use and excluded dual use where possible, many exclusive e-cigarette users may be former smokers. Lipid abnormalities caused by previous combustible tobacco exposure may persist after switching to e-cigarettes, making it difficult to isolate the metabolic effect of vaping itself. Reverse causality is also possible, because individuals with cardiovascular risk factors may switch from cigarettes to e-cigarettes as a perceived harm-reduction strategy. Furthermore, most studies did not report key details regarding vaping behavior, such as the type of device used or duration of use, leaving important potential confounders unaccounted for. Small sample sizes were common, and some studies exhibited a risk of bias. Because most continuous lipid analyses were based on unadjusted mean differences, these findings should be interpreted as descriptive pooled differences rather than adjusted estimates of the independent association between vaping and lipid levels. Finally, we observed some heterogeneity among the included studies as well as evidence of publication bias, which may influence the robustness of the overall results. Overall, there is a compelling need for prospective and retrospective studies reporting dyslipidaemia as binary outcomes with adjusted effect sizes. Future cohort studies must account for confounders and factors such as vaping behaviour (including device type and duration), history of other forms of tobacco use or dual use, lifestyle habits such as diet and physical activity, and comorbidities that may influence lipid profiles.

In light of the above, establishing a link between vaping and dyslipidemia is important given the known association between abnormal lipid profiles and atherosclerotic cardiovascular disease (ASCVD). All components of serum lipids contribute to the pathogenesis of atherosclerosis, with LDL cholesterol being the primary culprit. LDL cholesterol is central to atherogenesis, as excess LDL infiltrates the vessel wall, becomes oxidized to OxLDL, and is engulfed by macrophages to form foam cells and plaques [53,54]. Hypertriglyceridemia also contributes via triglyceride-rich lipoproteins (TGRLs) that penetrate and are retained in the arterial wall [55]. Low HDL levels exacerbate this process by failing to prevent LDL oxidation and inflammation [56]. The clinical implications of such links are highlighted in landmark studies, including the Lipid Research Clinics Coronary Primary Prevention Trial and the Scandinavian Simvastatin Survival Study (4S), which have demonstrated the effectiveness of lipid-lowering therapies in reducing cardiovascular events [57,58].

Though marketed as safer alternatives, observational evidence suggests e-cigarettes may negatively impact lipid metabolism and cardiovascular health. Therefore, smoking cessation programs that do not promote switching to vaping should remain the primary strategy for reducing cardiovascular risk. Public health efforts must focus on young adults, who are particularly vulnerable to vaping-related lipid changes. Long-term robust population studies are needed to clarify these risks. Clinicians may consider cardiovascular risk assessment, including lipid evaluation, in chronic e-cigarette users who have additional risk factors, while continuing to emphasize evidence-based smoking and nicotine cessation strategies.

## 5. Conclusion

This systematic review and meta-analysis suggest possible differences in lipid profiles between exclusive e-cigarette users and non-users, including higher LDL, VLDL, triglycerides, and higher odds of low HDL or hypertriglyceridemia in some analyses. However, the evidence is derived mainly from observational studies; several continuous outcomes were based on unadjusted mean differences, heterogeneity was substantial for some outcomes; and GRADE certainty ranged from very low to low. Therefore, the current evidence does not support firm conclusions about causality or the direction and magnitude of association.

## Supporting information

S1 File PRISMA 2020 checklist. (DOCX)

S2 File Search strategy, supplementary tables, and figures. Includes Table S1: Values of Different Lipid Profile Outcomes, Table S2: MASTER scale, Figure S1: Quality assessment of included studies using the MASTER scale, Table S3: Certainty assessment of evidence for each outcome, Table S4: Summary of Findings (SoF), Figure S2: Doi plot assessing publication bias in the meta-analysis of total cholesterol (TC), Figure S3: Forest plot showing a comparison of mean TC levels between vapers and non-vapers in leave-one-out analysis, Figure S4: Doi plot assessing publication bias in the meta-analysis of HDL, Figure S5: Doi plot assessing publication bias in the meta-analysis of LDL, Figure S6: Forest plot showing a comparison of mean LDL levels between vapers and non-vapers in leave-one-out analysis, Figure S7: Doi plot assessing publication bias in the meta-analysis of TG.
(DOCX)
