# Supplementary material for: The association between e-cigarette use and dyslipidemia: A systematic review and meta-analysis of observational studies
Source: PLoS One. 2026 Oct 26;21(10):e0361200. doi: 10.1371/journal.pone.0361200 (PMC13704818; doi:10.1371/journal.pone.0361200)
Supplement: Supplementary file 1 [file pone.0361200.s004.pdf]

Peer review comments for “The Association Between E-cigarette and Dyslipidemia: A Systematic Review and Meta-analysis” (PONE-D-25-42648)

1) Overall / general impressions

- This is a timely question and the manuscript is clearly structured (PRISMA, PROSPERO registration, GRADE). The narrative is generally easy to follow, and the authors are transparent that most evidence is low certainty and largely observational.
- That said, the current evidence base is thin (7 studies; mostly cross-sectional), and the manuscript would benefit from tightening claims and strengthening how you handle confounding, “exclusive vaping” definitions, and the mixing of *adjusted binary outcomes* with *unadjusted continuous outcomes*. This is the biggest interpretability issue for readers.

2) Title / Abstract

- **Title:** Clear and appropriate. Consider clarifying in the title (or subtitle) that the included evidence is observational (since causality cannot be inferred).
- **Abstract (Methods):** You mention subgroup analyses (age/sex) but note they weren’t possible. That’s fine, but I’d recommend explicitly stating *why* (e.g., “insufficient stratified reporting”) to pre-empt reviewer concerns.
- **Abstract (Results):**
    - The reporting mixes adjusted odds ratios (aORs) for dyslipidemia outcomes with weighted mean differences from unadjusted means. I’d strongly suggest explicitly labeling the WMD results as “unadjusted” in the abstract to avoid readers interpreting them as causal/adjusted effects.
    - The TC result is reported as borderline (CI crosses 0). You might soften wording (“trend toward higher TC”) and keep emphasis proportional to certainty.
- **Abstract (Conclusion):** The phrase “vaping is likely associated” is a bit strong given “very low–low” certainty; consider “may be associated” or “is associated in limited observational data.”

3) Introduction

- The rationale is strong: you connect ENDS uptake, cardiovascular risk, and dyslipidemia as a plausible mechanism.
- Suggested improvements:
    - Clarify *why* dyslipidemia matters clinically in this context—e.g., whether small shifts in LDL/HDL in young adults could translate into long-term ASCVD risk (even if speculative). Keep it balanced and not alarmist.
    - You may want to pre-define what you mean by “exclusive vaping” in the intro (because readers will immediately worry about former smoking/dual use).

4) Methods

4.1 Study design / protocol

- Good: PRISMA 2020 + PROSPERO registration are clear strengths.

- Improvement: Briefly state any deviations from the registered protocol (if none, explicitly say “no deviations”). Reviewers often look for this.

4.2 Search strategy

- You searched major databases plus Google Scholar and updated to May 31, 2025—excellent coverage.
- Improvement suggestions:
  - Please report whether you applied any Google Scholar screening limits (e.g., first N pages/results). Without this, reproducibility is hard.
  - Consider adding the full search strings (not just “keywords”) in the supplement if not already there.

4.3 Eligibility criteria (big one)

- You exclude dual users, but you also allow participants “regardless of past vaping or smoking history.” That opens a major confounding pathway: many “exclusive vapers” are former smokers, and prior smoking could drive lipid differences. I’d recommend:
  - Either restricting to never-smoker vapers (if data allow), or
  - Pre-specifying a subgroup/sensitivity analysis: “exclusive vapers who never smoked” vs “exclusive vapers who are former smokers,” or
  - At minimum, clearly describing this as a key limitation up front and in GRADE downgrading decisions.

4.4 Outcomes

- The dyslipidemia cutoffs are provided—helpful.
- Improvement: specify whether you accepted each study’s *own* dyslipidemia definitions if different, and how you harmonized them (or whether you didn’t). Outcome definition variability can create heterogeneity.

4.5 Data extraction

- The paired extraction process is reassuring.
- Improvement: add a short list of “core confounders” you attempted to extract (age, sex, BMI, smoking history, alcohol, diet, activity, socioeconomic status). This helps readers assess residual confounding.

4.6 Risk of bias / quality (MASTER)

- Using MASTER is interesting and defensible, but many readers are more familiar with NOS/ROBINS-I. Consider adding 1–2 sentences justifying why MASTER was chosen and how to interpret its scores.

4.7 Synthesis / statistics

- You use a quality-effects (inverse heterogeneity) model and also random effects for comparison. That’s thoughtful.
- Major improvement opportunity:
  - Mixing adjusted ORs (binary outcomes) with unadjusted mean differences (continuous outcomes) across outcomes creates an “apples vs oranges” feel. Consider reorganizing Results so it is crystal-clear which outcomes are adjusted vs unadjusted, and avoid implying they carry similar evidential weight.
  - For very high heterogeneity (e.g., TG I^2 ~99%), consider whether pooling is meaningful; at minimum, add interpretive caution (“summary effect may not represent a common effect”).

5) Results

5.1 Study selection / PRISMA

- Flow is clearly reported (4,160 identified; 7 included).
- Suggestion: include the full exclusion table (with reasons per full-text) in supplement if not already.

5.2 Study characteristics (Table 1)

- Helpful table, but I'd encourage you to add columns for:
  - how "exclusive vaping" was defined (self-report? timeframe?)
  - smoking history (never/former/unknown)
  - lipid measurement method (fasting vs non-fasting; lab vs self-report)
  These features are likely drivers of heterogeneity and bias.

5.3 Lipid-specific findings

- **TC:** high heterogeneity and publication bias signals; your leave-one-out sensitivity suggests one study drives uncertainty. Consider highlighting *which clinical or design features* of that study differ (population? fasting status? control definition?).
- **HDL & TG (adjusted ORs):** the adjusted analyses are the most convincing pieces here—worth emphasizing, but also clarify which confounders were adjusted for in each study (in text or supplement).
- **LDL/VLDL:** results appear more consistent but still largely unadjusted. Please keep wording cautious and avoid clinical recommendations based solely on unadjusted differences.

5.4 GRADE

- You state evidence ranges very low–low and explain downgrades (heterogeneity, bias, publication bias concerns). That's aligned with the dataset.
- Suggestion: present a compact "Summary of Findings" table in the main text (even if detailed tables remain supplementary). For readers, this is often the single most useful output of a review.

---

6) Discussion

- Strengths section is fair and transparent; you explain plausible mechanisms (nicotine → catecholamines → lipolysis → FFAs → TG/VLDL).
- Improvements to consider:
  - Tighten causal language: cross-sectional designs + residual confounding should push you toward "association," not "effect."
  - Expand the smoking-history issue: former smoking is likely the biggest alternative explanation, and it deserves more prominence (not just "limitations" but interpretation).
  - Consider discussing reverse causality / health selection: people with dyslipidemia or health concerns may switch from cigarettes to vaping (or vice versa), which can bias associations.
  - When comparing to cigarette or waterpipe studies, keep it explicit that the mechanisms might overlap (nicotine/inflammation), but exposure profiles differ (device types, aerosols, etc.). This will make the discussion feel more nuanced and less "cigarette-analogy dependent."

7) Conclusion

- The conclusion is aligned with the findings, but I recommend making the "low certainty" message even more prominent, since this is what readers and journalists will quote.
- Consider a final sentence that specifies what "robust longitudinal studies" should measure: device type, nicotine concentration, duration/intensity, fasting lipids, validated smoking history, and ideally repeated measures.

Overall recommendation
Major revision.
The topic is important and the review is promising, but the manuscript needs clearer handling and communication of confounding (especially smoking history), clearer separation of adjusted vs unadjusted evidence streams, and more cautious interpretation where heterogeneity is extreme. With these improvements, it could become a solid and useful synthesis.

Zypher Jude G. Regencia, Ph.D.
Associate Professor, Department of Sociology and Behavioral Sciences,
College of Liberal Arts, De La Salle University
Senior Consultant, Data and Implementation Sciences for Health
