# Supplementary material for: The association between e-cigarette use and dyslipidemia: A systematic review and meta-analysis of observational studies
Source: PLoS One. 2026 Oct 26;21(10):e0361200. doi: 10.1371/journal.pone.0361200 (PMC13704818; doi:10.1371/journal.pone.0361200)
Supplement: Supplementary file 3 [file pone.0361200.s002.docx]

**Search Strategy**

**Included Datasets**: Pubmed, Cochrane CENTRAL, Scopus CINAHL, EMBASE, Google Scholar

**Pubmed**: 748

**Dyslipidemia** **Terms**: [2,263,231](https://pubmed.ncbi.nlm.nih.gov/?term=%28%28%28%28%28%28%28%28%28%28%28%28%28%28%28%28%28%28Lipids%5BMeSH+Terms%5D%29+OR+%28Triglycerides%5BMeSH+Terms%5D%29%29+OR+%28Cholesterol%5BMeSH+Terms%5D%29%29+OR+%28Hyperlipidemias%5BMeSH+Terms%5D%29%29+OR+%28Dyslipidemias%5BMeSH+Terms%5D%29%29+OR+%28Hypercholesterolemia%5BMeSH+Terms%5D%29%29+OR+%28lipid%2A%5BTitle%2FAbstract%5D%29%29+OR+%28hyperlipid%2A%5BTitle%2FAbstract%5D%29%29+OR+%28%28%28%28%28fats%5BTitle%2FAbstract%5D%29+OR+%28fat%5BTitle%2FAbstract%5D%29%29+OR+%28%22fatty+acids%22%5BTitle%2FAbstract%5D%29%29+OR+%28fatty%5BTitle%2FAbstract%5D%29%29+OR+%28fatness%5BTitle%2FAbstract%5D%29%29%29+OR+%28lipoprotein%2A%5BTitle%2FAbstract%5D%29%29+OR+%28hyperlipoprotein%2A%5BTitle%2FAbstract%5D%29%29+OR+%28triglycerid%2A%5BTitle%2FAbstract%5D%29%29+OR+%28hypertriglycerid%2A%5BTitle%2FAbstract%5D%29%29+OR+%28cholesterol%2A%5BTitle%2FAbstract%5D%29%29+OR+%28chol%5BTitle%2FAbstract%5D%29%29+OR+%28hypercholesterol%2A%5BTitle%2FAbstract%5D%29%29+OR+%28dyslipid%2A%5BTitle%2FAbstract%5D%29%29+OR+%28hyperdyslipid%2A%5BTitle%2FAbstract%5D%29%29+OR+%28%28%28%28%28%28%28%28%28%28%28%28HDL%5BTitle%2FAbstract%5D%29+OR+%28HDLs%5BTitle%2FAbstract%5D%29%29+OR+%28LDL%5BTitle%2FAbstract%5D%29%29+OR+%28LDLs%5BTitle%2FAbstract%5D%29%29+OR+%28VLDL%5BTitle%2FAbstract%5D%29%29+OR+%28VLDLs%5BTitle%2FAbstract%5D%29%29+OR+%28IDL%5BTitle%2FAbstract%5D%29%29+OR+%28IDLs%5BTitle%2FAbstract%5D%29%29+OR+%28Lp%5BTitle%2FAbstract%5D%29%29+OR+%28Lps%5BTitle%2FAbstract%5D%29%29+OR+%28TG%5BTitle%2FAbstract%5D%29%29+OR+%28TGs%5BTitle%2FAbstract%5D%29%29&sort=relevance)

((((((((((((((((((Lipids[MeSH Terms]) OR (Triglycerides[MeSH Terms])) OR (Cholesterol[MeSH Terms])) OR (Hyperlipidemias[MeSH Terms])) OR (Dyslipidemias[MeSH Terms])) OR (Hypercholesterolemia[MeSH Terms])) OR (lipid*[Title/Abstract])) OR (hyperlipid*[Title/Abstract])) OR (((((fats[Title/Abstract]) OR (fat[Title/Abstract])) OR ("fatty acids"[Title/Abstract])) OR (fatty[Title/Abstract])) OR (fatness[Title/Abstract]))) OR (lipoprotein*[Title/Abstract])) OR (hyperlipoprotein*[Title/Abstract])) OR (triglycerid*[Title/Abstract])) OR (hypertriglycerid*[Title/Abstract])) OR (cholesterol*[Title/Abstract])) OR (chol[Title/Abstract])) OR (hypercholesterol*[Title/Abstract])) OR (dyslipid*[Title/Abstract])) OR (hyperdyslipid*[Title/Abstract])) OR ((((((((((((HDL[Title/Abstract]) OR (HDLs[Title/Abstract])) OR (LDL[Title/Abstract])) OR (LDLs[Title/Abstract])) OR (VLDL[Title/Abstract])) OR (VLDLs[Title/Abstract])) OR (IDL[Title/Abstract])) OR (IDLs[Title/Abstract])) OR (Lp[Title/Abstract])) OR (Lps[Title/Abstract])) OR (TG[Title/Abstract])) OR (TGs[Title/Abstract]))

**Vaping Terms**: [23,203](https://pubmed.ncbi.nlm.nih.gov/?term=%28%28%28%28%28%28%28%28%28%28%28%28Tobacco%2C+Waterpipe%5BMeSH+Terms%5D%29+OR+%28Smoking+Water+Pipes%5BMeSH+Terms%5D%29%29+OR+%28E-Cigarette+Vapor%5BMeSH+Terms%5D%29%29+OR+%28Electronic+Nicotine+Delivery+Systems%5BMeSH+Terms%5D%29%29+OR+%28Vaping%5BMeSH+Terms%5D%29%29+OR+%28Smoking+Pipes%5BMeSH+Terms%5D%29%29+OR+%28Pipe+Smoking%5BMeSH+Terms%5D%29%29+OR+%28electronic+nicotine%5BTitle%2FAbstract%5D%29%29+OR+%28e-nicotine%5BTitle%2FAbstract%5D%29%29+OR+%28%22e+nicotine%22%5BTitle%2FAbstract%5D%29%29+OR+%28%28vape%5BTitle%2FAbstract%5D%29+OR+%28vaping%5BTitle%2FAbstract%5D%29%29%29+OR+%28%28%28%28%28%28%28%28%28%28%22electronic+smoking%22%5BTitle%2FAbstract%5D%29+OR+%28%22electronic+cigar%2A%22%5BTitle%2FAbstract%5D%29%29+OR+%28%22E-cig%22%5BTitle%2FAbstract%5D%29%29+OR+%28%22E-cigs%22%5BTitle%2FAbstract%5D%29%29+OR+%28%22E+cig%22%5BTitle%2FAbstract%5D%29%29+OR+%28%22E+cigs%22%5BTitle%2FAbstract%5D%29%29+OR+%28%22E-cigar%22%5BTitle%2FAbstract%5D%29%29+OR+%28%22E-cigars%22%5BTitle%2FAbstract%5D%29%29+OR+%28%22E+cigar%22%5BTitle%2FAbstract%5D%29%29+OR+%28%22E+cigars%22%5BTitle%2FAbstract%5D%29%29%29+OR+%28%28%28%28%28%28%28%28%28%28%28%28%28%22water+pipe%2A%22%5BTitle%2FAbstract%5D%29+OR+%28waterpipe%5BTitle%2FAbstract%5D%29%29+OR+%28pipe%5BTitle%2FAbstract%5D%29%29+OR+%28hookah%5BTitle%2FAbstract%5D%29%29+OR+%28shisha%5BTitle%2FAbstract%5D%29%29+OR+%28Arghile%5BTitle%2FAbstract%5D%29%29+OR+%28Chicha%5BTitle%2FAbstract%5D%29%29+OR+%28Nargile%5BTitle%2FAbstract%5D%29%29%29+OR+%28%22Hubble-bubble%22%5BTitle%2FAbstract%5D%29%29+OR+%28%22Hubble+bubble%22%5BTitle%2FAbstract%5D%29%29+OR+%28Ghalyan%5BTitle%2FAbstract%5D%29%29+OR+%28Qalyan%5BTitle%2FAbstract%5D%29%29&sort=relevance&ac=no)

((((((((((((Tobacco, Waterpipe[MeSH Terms]) OR (Smoking Water Pipes[MeSH Terms])) OR (E-Cigarette Vapor[MeSH Terms])) OR (Electronic Nicotine Delivery Systems[MeSH Terms])) OR (Vaping[MeSH Terms])) OR (Smoking Pipes[MeSH Terms])) OR (Pipe Smoking[MeSH Terms])) OR (electronic nicotine[Title/Abstract])) OR (e-nicotine[Title/Abstract])) OR ("e nicotine"[Title/Abstract])) OR ((vape[Title/Abstract]) OR (vaping[Title/Abstract]))) OR (((((((((("electronic smoking"[Title/Abstract]) OR ("electronic cigar*"[Title/Abstract])) OR ("E-cig"[Title/Abstract])) OR ("E-cigs"[Title/Abstract])) OR ("E cig"[Title/Abstract])) OR ("E cigs"[Title/Abstract])) OR ("E-cigar"[Title/Abstract])) OR ("E-cigars"[Title/Abstract])) OR ("E cigar"[Title/Abstract])) OR ("E cigars"[Title/Abstract]))) OR ((((((((((((("water pipe*"[Title/Abstract]) OR (waterpipe[Title/Abstract])) OR (pipe[Title/Abstract])) OR (hookah[Title/Abstract])) OR (shisha[Title/Abstract])) OR (Arghile[Title/Abstract])) OR (Chicha[Title/Abstract])) OR (Nargile[Title/Abstract]))) OR ("Hubble-bubble"[Title/Abstract])) OR ("Hubble bubble"[Title/Abstract])) OR (Ghalyan[Title/Abstract])) OR (Qalyan[Title/Abstract]))

**Together**: 748

(((((((((((((((((((Lipids[MeSH Terms]) OR (Triglycerides[MeSH Terms])) OR (Cholesterol[MeSH Terms])) OR (Hyperlipidemias[MeSH Terms])) OR (Dyslipidemias[MeSH Terms])) OR (Hypercholesterolemia[MeSH Terms])) OR (lipid*[Title/Abstract])) OR (hyperlipid*[Title/Abstract])) OR (((((fats[Title/Abstract]) OR (fat[Title/Abstract])) OR ("fatty acids"[Title/Abstract])) OR (fatty[Title/Abstract])) OR (fatness[Title/Abstract]))) OR (lipoprotein*[Title/Abstract])) OR (hyperlipoprotein*[Title/Abstract])) OR (triglycerid*[Title/Abstract])) OR (hypertriglycerid*[Title/Abstract])) OR (cholesterol*[Title/Abstract])) OR (chol[Title/Abstract])) OR (hypercholesterol*[Title/Abstract])) OR (dyslipid*[Title/Abstract])) OR (hyperdyslipid*[Title/Abstract])) OR ((((((((((((HDL[Title/Abstract]) OR (HDLs[Title/Abstract])) OR (LDL[Title/Abstract])) OR (LDLs[Title/Abstract])) OR (VLDL[Title/Abstract])) OR (VLDLs[Title/Abstract])) OR (IDL[Title/Abstract])) OR (IDLs[Title/Abstract])) OR (Lp[Title/Abstract])) OR (Lps[Title/Abstract])) OR (TG[Title/Abstract])) OR (TGs[Title/Abstract]))) AND (((((((((((((Tobacco, Waterpipe[MeSH Terms]) OR (Smoking Water Pipes[MeSH Terms])) OR (E-Cigarette Vapor[MeSH Terms])) OR (Electronic Nicotine Delivery Systems[MeSH Terms])) OR (Vaping[MeSH Terms])) OR (Smoking Pipes[MeSH Terms])) OR (Pipe Smoking[MeSH Terms])) OR (electronic nicotine[Title/Abstract])) OR (e-nicotine[Title/Abstract])) OR ("e nicotine"[Title/Abstract])) OR ((vape[Title/Abstract]) OR (vaping[Title/Abstract]))) OR (((((((((("electronic smoking"[Title/Abstract]) OR ("electronic cigar*"[Title/Abstract])) OR ("E-cig"[Title/Abstract])) OR ("E-cigs"[Title/Abstract])) OR ("E cig"[Title/Abstract])) OR ("E cigs"[Title/Abstract])) OR ("E-cigar"[Title/Abstract])) OR ("E-cigars"[Title/Abstract])) OR ("E cigar"[Title/Abstract])) OR ("E cigars"[Title/Abstract]))) OR ((((((((((((("water pipe*"[Title/Abstract]) OR (waterpipe[Title/Abstract])) OR (pipe[Title/Abstract])) OR (hookah[Title/Abstract])) OR (shisha[Title/Abstract])) OR (Arghile[Title/Abstract])) OR (Chicha[Title/Abstract])) OR (Nargile[Title/Abstract]))) OR ("Hubble-bubble"[Title/Abstract])) OR ("Hubble bubble"[Title/Abstract])) OR (Ghalyan[Title/Abstract])) OR (Qalyan[Title/Abstract])))

**Cochrane CENTRA**L: 141

**Dyslipidemia Terms**: 170867

#1	MeSH descriptor: [Lipids] explode all trees	64972

#2	MeSH descriptor: [Triglycerides] explode all trees	8073

#3	MeSH descriptor: [Cholesterol] explode all trees	13181

#4	MeSH descriptor: [Hyperlipidemias] explode all trees	8065

#5	MeSH descriptor: [Dyslipidemias] explode all trees	9695

#6	MeSH descriptor: [Hypercholesterolemia] explode all trees	4298

#7	(hyperlipid* or lipid*):ti,ab,kw (Word variations have been searched)	60195

#8	(fat or fats or "fatty acids" or fatty or fatness):ti,ab,kw (Word variations have been searched)	67590

#9	(lipoprotein* or hyperlipoprotein*):ti,ab,kw (Word variations have been searched)	27841

#10	(triglycerid* or hypertriglycerid*):ti,ab,kw (Word variations have been searched)	29739

#11	(cholesterol* or hypercholesterol*):ti,ab,kw (Word variations have been searched)	48403

#12	(dyslipid* or hyperdyslipid*):ti,ab,kw (Word variations have been searched)	8001

#13	(HDL or HDLs or LDL or LDLs or VLDL or VLDLs or Lp or Lps or IDL or IDLs or TG or TGs or Chol):ti,ab,kw (Word variations have been searched)	41771

#14	#1 OR #2 OR #3 OR #4 OR #5 OR #6 OR #7 OR #8 OR #9 OR #10 OR #11 OR #12 OR #13	170867

**Vaping Terms**: 2091

#15	MeSH descriptor: [Tobacco, Waterpipe] explode all trees	30

#16	MeSH descriptor: [Smoking Water Pipes] explode all trees	19

#17	MeSH descriptor: [E-Cigarette Vapor] explode all trees	5

#18	MeSH descriptor: [Electronic Nicotine Delivery Systems] explode all trees	517

#19	MeSH descriptor: [Vaping] explode all trees	213

#20	MeSH descriptor: [Smoking Pipes] explode all trees	19

#21	MeSH descriptor: [Pipe Smoking] explode all trees	41

#22	((Electronic NEXT nicotine) OR (e-nicotine) OR (e NEXT nicotine) OR (Vape) OR (Vaping) OR (Electronic NEXT smoking) OR (Electronic NEXT cigar*) OR (E-cig) OR (E-cigs) OR (E NEXT cig) OR (E NEXT cigs) OR (E-cigar) OR (E-cigars) OR (E NEXT cigar) OR (E NEXT cigars)):ti,ab,kw (Word variations have been searched)	1206

#23	((Water pipe*) OR (waterpipe) OR (pipe) OR (hookah) OR (shisha) OR (Arghile) OR (Chicha) OR (Nargile) OR (Hubble-bubble) OR (Hubble bubble) OR (Ghalyan) OR (Qalyan)):ti,ab,kw (Word variations have been searched)	914

#24	#15 OR #16 OR #17 OR #18 OR #19 OR #20 OR #21 OR #22 OR #23	2091

**Together**: [139](https://www-cochranelibrary-com.qulib.idm.oclc.org/en/advanced-search/search-manager?searchBy=-1&searchText=&isWordVariations=&resultPerPage=25&searchType=searchManager&forceTypeSelection=true&selectedType=central&displayText=&orderBy=relevancy&p_p_id=scolarissearchresultsportlet_WAR_scolarissearchresults&p_p_lifecycle=0&p_p_state=normal&p_p_mode=view&p_p_col_id=column-1&p_p_col_pos=1&p_p_col_count=2)

#25	#14 AND #24

**Scopus**: 1,947

**Dyslipidemia Terms**: 2,872,019

TITLE-ABS-KEY((Lipid*) OR (lipid) OR (lipids) OR (lipidemia) OR (lipidaemia) OR (lipiduria) OR (lipidopathy) OR (lipidic) OR (lipidemic) OR (Hyperlipid*) OR (Hyperlipidemia) OR (Hyperlipidaemia) OR (Hyperlipidemic) OR (Hyperlipidaemic) OR (Hyperlipiduric) OR (Fat) OR (fats) OR ("fatty acids") OR (fatty) OR (fatness) OR (Lipoprotein*) OR (lipoprotein) OR (lipoproteinemia) OR (lipoproteinemic) OR (lipoproteinase) OR (lipoproteinuria) OR (HDL) OR (HDLs) OR (LDL) OR (LDLs) OR (VLDL) OR (VLDLs) OR (Lp) OR (Lps) OR (IDL) OR (IDLs) OR (Hyperlipoprotein*) OR (Hyperlipoproteinemia) OR (hyperlipoproteinaemia) OR (Hyperlipoproteinemic) OR (hyperlipoproteinaemic) OR (Hyperlipoproteinuria) OR (Triglycerid*) OR (Triglyceride) OR (Triglyceridemia) OR (Triglyceridaemia) OR (Triglyceridemic) OR (TG) OR (TGs) OR (Hypertriglycerid*) OR (Hypertriglyceridaemic) OR (Hypertriglyceridemia) OR (Hypertriglyceridemic) OR (Hypertriglyceridaemia) OR (Cholesterol*) OR (cholesterolemia) OR (cholesterolaemia) OR (Chol) OR (Hypercholesterol*) OR (Hypercholesterolemia) OR (Hypercholesterolaemia) OR (Dyslipid*) OR (Dyslipidemia) OR (dyslipidaemia) OR (Dyslipidemic) OR (dyslipidaemic) OR (Hyperdyslipid*) OR (Hyperdyslipidemia) OR (hyperdyslipidaemia) OR (hyperdyslipidemic) OR (hyperdyslipidaemic))

**Vaping Terms**: 306,875

TITLE-ABS-KEY(("Electronic nicotine") OR ("e-nicotine") OR ("e nicotine") OR (Vape) OR (Vaping) OR ("Electronic smoking") OR ("Electronic cigar*") OR ("E-cig") OR ("E-cigs") OR ("E cig") OR ("E cigs") OR ("E-cigar") OR ("E-cigars") OR ("E cigar") OR ("E cigars") OR ("Water pipe*") OR (waterpipe) OR (pipe) OR (hookah) OR (shisha) OR (Arghile) OR (Chicha) OR (Nargile) OR ("Hubble-bubble") OR ("Hubble bubble") OR (Ghalyan) OR (Qalyan))

**Together**: 1,947

TITLE-ABS-KEY(((Lipid*) OR (lipid) OR (lipids) OR (lipidemia) OR (lipidaemia) OR (lipiduria) OR (lipidopathy) OR (lipidic) OR (lipidemic) OR (Hyperlipid*) OR (Hyperlipidemia) OR (Hyperlipidaemia) OR (Hyperlipidemic) OR (Hyperlipidaemic) OR (Hyperlipiduric) OR (Fat) OR (fats) OR ("fatty acids") OR (fatty) OR (fatness) OR (Lipoprotein*) OR (lipoprotein) OR (lipoproteinemia) OR (lipoproteinemic) OR (lipoproteinase) OR (lipoproteinuria) OR (HDL) OR (HDLs) OR (LDL) OR (LDLs) OR (VLDL) OR (VLDLs) OR (Lp) OR (Lps) OR (IDL) OR (IDLs) OR (Hyperlipoprotein*) OR (Hyperlipoproteinemia) OR (hyperlipoproteinaemia) OR (Hyperlipoproteinemic) OR (hyperlipoproteinaemic) OR (Hyperlipoproteinuria) OR (Triglycerid*) OR (Triglyceride) OR (Triglyceridemia) OR (Triglyceridaemia) OR (Triglyceridemic) OR (TG) OR (TGs) OR (Hypertriglycerid*) OR (Hypertriglyceridaemic) OR (Hypertriglyceridemia) OR (Hypertriglyceridemic) OR (Hypertriglyceridaemia) OR (Cholesterol*) OR (cholesterolemia) OR (cholesterolaemia) OR (Chol) OR (Hypercholesterol*) OR (Hypercholesterolemia) OR (Hypercholesterolaemia) OR (Dyslipid*) OR (Dyslipidemia) OR (dyslipidaemia) OR (Dyslipidemic) OR (dyslipidaemic) OR (Hyperdyslipid*) OR (Hyperdyslipidemia) OR (hyperdyslipidaemia) OR (hyperdyslipidemic) OR (hyperdyslipidaemic)) AND (("Electronic nicotine") OR ("e-nicotine") OR ("e nicotine") OR (Vape) OR (Vaping) OR ("Electronic smoking") OR ("Electronic cigar*") OR ("E-cig") OR ("E-cigs") OR ("E cig") OR ("E cigs") OR ("E-cigar") OR ("E-cigars") OR ("E cigar") OR ("E cigars") OR ("Water pipe*") OR (waterpipe) OR (pipe) OR (hookah) OR (shisha) OR (Arghile) OR (Chicha) OR (Nargile) OR ("Hubble-bubble") OR ("Hubble bubble") OR (Ghalyan) OR (Qalyan)))

**CINAHL**: 120

**Dyslipidemia Terms**: 269,732

S1: (MH "Lipids+") | Search modes - Find any of my search terms | Interface - EBSCOhost Research Databases | Search Screen - Advanced Search | Database - CINAHL Ultimate | 135,247

S2: (MH "Triglycerides") | Expanders - Apply equivalent subjects | Search modes - Find any of my search terms | Interface - EBSCOhost Research Databases | Search Screen - Advanced Search | Database - CINAHL Ultimate | 17,347

S3: (MH "Lipoproteins+") | Expanders - Apply equivalent subjects | Search modes - Find any of my search terms | Interface - EBSCOhost Research Databases | Search Screen - Advanced Search | Database - CINAHL Ultimate | 29,027

S4: (MH "Cholesterol+") | Expanders - Apply equivalent subjects | Search modes - Find any of my search terms | Interface - EBSCOhost Research Databases | Search Screen - Advanced Search | Database - CINAHL Ultimate | 31,918

S5: (MH "Hyperlipidemia+") | Expanders - Apply equivalent subjects | Search modes - Find any of my search terms | Interface - EBSCOhost Research Databases | Search Screen - Advanced Search | Database - CINAHL Ultimate | 23,892

S6: (MH "Hypercholesterolemia+") | Expanders - Apply equivalent subjects | Search modes - Find any of my search terms | Interface - EBSCOhost Research Databases | Search Screen - Advanced Search | Database - CINAHL Ultimate | 9,580

S7: TI lipid* OR AB lipid* | Expanders - Apply equivalent subjects | Search modes - Find any of my search terms | Interface - EBSCOhost Research Databases | Search Screen - Advanced Search | Database - CINAHL Ultimate | 57,794

S8: TI hyperlipid* OR AB hyperlipid* | Expanders - Apply equivalent subjects | Search modes - Find any of my search terms | Interface - EBSCOhost Research Databases | Search Screen - Advanced Search | Database - CINAHL Ultimate | 7,267

S9: TI lipoprotein* OR AB lipoprotein* OR TI hyperlipoprotein* OR AB hyperlipoprotein* | Expanders - Apply equivalent subjects | Search modes - Find any of my search terms | Interface - EBSCOhost Research Databases | Search Screen - Advanced Search | Database - CINAHL Ultimate | 27,191

S10: TI triglycerid* OR AB triglycerid* OR TI hypertriglycerid* OR AB hypertriglycerid* | Expanders - Apply equivalent subjects | Search modes - Find any of my search terms | Interface - EBSCOhost Research Databases | Search Screen - Advanced Search | Database - CINAHL Ultimate | 25,606

S11: TI cholesterol* OR AB cholesterol* OR TI hypercholesterol* OR AB hypercholesterol* | Expanders - Apply equivalent subjects | Search modes - Find any of my search terms | Interface - EBSCOhost Research Databases | Search Screen - Advanced Search | Database - CINAHL Ultimate | 51,051

S12: TI dyslipid* OR AB dyslipid* OR TI hyperdyslipid* OR AB hyperdyslipid* | Expanders - Apply equivalent subjects | Search modes - Find any of my search terms | Interface - EBSCOhost Research Databases | Search Screen - Advanced Search | Database - CINAHL Ultimate | 11,131

S13: TI fat OR fats OR "fatty acids" OR fatty OR fatness OR AB fat OR fats OR "fatty acids" OR fatty OR fatness | Expanders - Apply equivalent subjects | Search modes - Find any of my search terms | Interface - EBSCOhost Research Databases | Search Screen - Advanced Search | Database - CINAHL Ultimate | 99,799

S14: TI (HDL OR HDLs OR LDL OR LDLs OR VLDL OR VLDLs OR Lp OR Lps OR IDL OR IDLs OR TG OR TGs OR Chol) OR AB (HDL OR HDLs OR LDL OR LDLs OR VLDL OR VLDLs OR Lp OR Lps OR IDL OR IDLs OR TG OR TGs OR Chol) | Search modes - Find any of my search terms | Interface - EBSCOhost Research Databases | Search Screen - Advanced Search | Database - CINAHL Ultimate | 40,136

S15: S1 OR S2 OR S3 OR S4 OR S5 OR S6 OR S7 OR S8 OR S9 OR S10 OR S11 OR S12 OR S13 OR S14 | Expanders - Apply equivalent subjects | Search modes - Find any of my search terms | Interface - EBSCOhost Research Databases | Search Screen - Advanced Search | Database - CINAHL Ultimate | 269,732

**Vaping Terms**: 8,567

S16: (MH "Vaping") | Expanders - Apply equivalent subjects | Search modes - Find any of my search terms | Interface - EBSCOhost Research Databases | Search Screen - Advanced Search | Database - CINAHL Ultimate | 1,018

S17: (MH "Electronic Cigarettes") | Expanders - Apply equivalent subjects | Search modes - Find any of my search terms | Interface - EBSCOhost Research Databases | Search Screen - Advanced Search | Database - CINAHL Ultimate | 3,713

S18: TI ("Electronic nicotine" OR "e-nicotine" OR "e nicotine" OR Vape OR Vaping OR "Electronic smoking" OR "Electronic cigar*" OR "E-cig" OR "E-cigs" OR "E cig" OR "E cigs" OR "E-cigar" OR "E-cigars" OR "E cigar" OR "E cigars") OR AB ("Electronic nicotine" OR "e-nicotine" OR "e nicotine" OR Vape OR Vaping OR "Electronic smoking" OR "Electronic cigar*" OR "E-cig" OR "E-cigs" OR "E cig" OR "E cigs" OR "E-cigar" OR "E-cigars" OR "E cigar" OR "E cigars") | Expanders - Apply equivalent subjects | Search modes - Find any of my search terms | Interface - EBSCOhost Research Databases | Search Screen - Advanced Search | Database - CINAHL Ultimate | 4,805

S19: TI ("Water pipe*" OR "waterpipe" OR "pipe" OR "hookah" OR "shisha" OR "Arghile" OR "Chicha" OR "Nargile" OR "Hubble-bubble" OR "Hubble bubble" OR "Ghalyan" OR "Qalyan") OR AB ("Water pipe*" OR "waterpipe" OR "pipe" OR "hookah" OR "shisha" OR "Arghile" OR "Chicha" OR "Nargile" OR "Hubble-bubble" OR "Hubble bubble" OR "Ghalyan" OR "Qalyan") | Expanders - Apply equivalent subjects | Search modes - Find any of my search terms | Interface - EBSCOhost Research Databases | Search Screen - Advanced Search | Database - CINAHL Ultimate | 2,061

S20: S16 OR S17 OR S18 OR S19 | Expanders - Apply equivalent subjects | Search modes - Proximity | Interface - EBSCOhost Research Databases | Search Screen - Advanced Search | Database - CINAHL Ultimate | 8,567

**Together**: 120

S21: (S15 AND S20) | Limiters - Research Article | Expanders - Apply equivalent subjects | Search modes - Find all my search terms | Interface - EBSCOhost Research Databases | Search Screen - Advanced Search | Database - CINAHL Ultimate | 63

**EMBASE**: 1,204

**Dyslipidemia Terms**: 3,285,336

#1 'lipid'/exp - 2,166,473

#2 'triacylglycerol'/exp - 283,530

#3 'cholesterol'/exp - 421,203

#4 'hyperlipidemia'/exp - 216,044

#5 'dyslipidemia'/exp - 114,498

#6 'hypercholesterolemia'/exp - 92,042

#7 'hyperlipid*':ab,ti OR 'lipid*':ab,ti OR 'fat':ab,ti OR 'fats':ab,ti OR 'fatty acids':ab,ti OR 'fatty':ab,ti OR 'fatness':ab,ti - 1,487,231

#8 lipoprotein*:ab,ti OR hyperlipoprotein*:ab,ti OR hdl:ab,ti OR hdls:ab,ti OR ldl:ab,ti OR ldls:ab,ti OR vldl:ab,ti OR vldls:ab,ti OR lp:ab,ti OR lps:ab,ti OR idl:ab,ti OR idls:ab,ti - 501,370

#9 triglycerid*:ab,ti OR hypertriglycerid*:ab,ti OR tg:ab,ti OR tgs:ab,ti OR cholesterol*:ab,ti OR chol:ab,ti - 557,616

#10 hypercholesterol*:ab,ti OR dyslipid*:ab,ti OR hyperdyslipid*:ab,ti - 141,574

#11 #1 OR #2 OR #3 OR #4 OR #5 OR #6 OR #7 OR #8 OR #9 OR #10 - 3,285,336

**Vaping Terms**: 30,318

#12 'hookah'/exp - 884

#13 'electronic cigarette'/exp - 13,904

#14 'electronic cigarette vapor'/exp - 345

#15 'vaping'/exp - 7,134

#16 'pipe smoking'/exp - 995

#17 'pipe smoking'/exp - 995

#18 'waterpipe tobacco'/exp - 485

#19 'electronic nicotine':ab,ti OR 'e-nicotine':ab,ti OR 'e nicotine':ab,ti OR vape:ab,ti OR vaping:ab,ti OR 'electronic smoking':ab,ti OR 'electronic cigar*':ab,ti OR 'e-cig':ab,ti OR 'e-cigs':ab,ti OR 'e cig':ab,ti OR 'e cigs':ab,ti OR 'e-cigar':ab,ti OR 'e-cigars':ab,ti OR 'e cigar':ab,ti OR 'e cigars':ab,ti - 11,106

#20 'water pipe*':ab,ti OR waterpipe:ab,ti OR pipe:ab,ti OR hookah:ab,ti OR shisha:ab,ti OR arghile:ab,ti OR chicha:ab,ti OR nargile:ab,ti OR 'hubble-bubble':ab,ti OR 'hubble bubble':ab,ti OR ghalyan:ab,ti OR qalyan:ab,ti - 12,927

#21 #12 OR #13 OR #14 OR #15 OR #16 OR #17 OR #18 OR #19 OR #20 - 30,318

**Together**: 1,204

#11 AND #23

**Google Scholar**

To ensure comprehensive identification of relevant studies, we conducted a supplementary search of Google Scholar on May 31^st^ 2025 using the same search terms applied in our primary database searches. To mitigate algorithmic personalization bias, searches were performed in an incognito/private browsing window with location settings standardized to a neutral region. We screened the first 30 pages of results (300 records total, based on Google Scholar's default 10 results per page), which aligns with methodological guidance for systematic reviews using Google Scholar (1, 2). This threshold is widely accepted as sufficient to capture >90% of relevant literature while balancing feasibility, as retrieval yield diminishes substantially beyond this point (1, 2). All retrieved records were imported into our reference management software, deduplicated against our existing database results, and screened against our eligibility criteria using the same dual-reviewer process applied to primary database searches. No additional eligible studies were identified beyond those already captured through our primary electronic database searches above.

**Search string**: (lipids OR triglycerides OR cholesterol OR hyperlipidemia OR dyslipidemia OR HDL OR LDL OR VLDL) AND (waterpipe OR hookah OR shisha OR "e-cigarette" OR vaping OR "electronic nicotine" OR "pipe smoking" OR arghile)

**Supplementary Table S1: Values of Different Lipid Profile outcomes.**

| **Author, Year** | **Vaping TC, mean(SD)** | **Control TC, mean(SD)** | **Vaping HDL, mean(SD)** | **Control HDL, mean(SD)** | **Vaping HDL, OR(LCI-UCI)** | **Vaping LDL, mean(SD)** | **Control LDL, mean(SD)** | **Vaping VLDL, mean(SD)** | **Control VLDL, mean(SD)** | **Vaping TG, mean(SD)** | **Control TG, mean(SD)** | **Vaping TG, OR(LCI-UCI)** |
| --- | --- | --- | --- | --- | --- | --- | --- | --- | --- | --- | --- | --- |
| **Hasan, 2024 (3)** | 246.4 (39.13) | 216.93 (24.58) | 33.99 (3.57) | 34.09 (2.22) | - | 129.88 (8.71) | 117.15 (2.74) | - | - | 174.43 (32.87) | 130.77 (25.61) | - |
| **Lim, 2024 (4)** | 195.3 (2.41) | 193.7 (0.6) | 52.2 (0.69) | 53.49 (0.26) | - | - | - | - | - | 143.9 (5.9) | 120.7 (1.71) | - |
| **Matheson, 2024 (5)** | 136 (73) | 121 (79) | 54 (14) | 40 (25) | - | 92 (32) | 64 (51) | - | - | 104 (61) | 88 (92) | - |
| **Okafor, 2022 (6)** | - | - | - | - | 1.82 (0.95-3.49) | - | - | - | - | - | - | 0.42 (0.12-1.51) |
| **Majid, 2021 (7)** | 152 (26) | 147 (26) | 45 (13) | 51 (11) | - | 89 (20) | 82 (22) | 17 (7) | 14 (5) | 91 (80) | 70 (26) | - |
| **Kim, 2020 (8)** | - | - | 47.1 (0.7) | 48.2 (0.2) | 1.14 (0.86-1.52) | - | - | - | - | 185.3 (10.4) | 136.4 (1.5) | 1.32 (1-1.74) |
| **Badea, 2019 (9)** | 173.24 (27.19) | 164.29 (33.68) | 55.29 (13.66) | 58.72 (12.28) | - | 112.03 (26.67) | 100.16 (32.1) | 20.15 (12.4) | 15.59 (6.84) | 100.91 (61.95) | 78.22 (34.07) | - |

*TC; Total Cholesterol. HDL; High Density Lipoprotein. LDL; Low Density Lipoprotein. VLDL; Very Low-Density Lipoprotein. TG; Triglycerides.*

**Supplementary Table S2: MASTER scale**

| **Study** | **Q1** | **Q2** | **Q3** | **Q4** | **Q5** | **Q6** | **Q7** | **Q8** | **Q9** | **Q10** | **Q11** | **Q12** | **Q13** | **Q14** | **Q15** | **Q16** | **Q17** | **Q18** | **Q19** | **Q20** | **Q21** | **Q22** | **Q23** | **Q24** | **Q25** | **Q26** | **Q27** | **Q28** | **Q29** | **Q30** | **Q31** | **Q32** | **Q33** | **Q34** | **Q35** | **Q36** | **MASTER Scale** |
| --- | --- | --- | --- | --- | --- | --- | --- | --- | --- | --- | --- | --- | --- | --- | --- | --- | --- | --- | --- | --- | --- | --- | --- | --- | --- | --- | --- | --- | --- | --- | --- | --- | --- | --- | --- | --- | --- |
| **Hasan, 2024 (3)** | 1 | 1 | 1 | 1 | 1 | 1 | 1 | 1 | 0 | 1 | 1 | 1 | 0 | 0 | 0 | 0 | 1 | 1 | 1 | 1 | 1 | 1 | 1 | 1 | 1 | 0 | 0 | 1 | 1 | 1 | 1 | 0 | 1 | 1 | 1 | 0 | 27 |
| **Lim, 2024 (4)** | 1 | 1 | 0 | 1 | 1 | 1 | 0 | 1 | 0 | 1 | 1 | 1 | 0 | 0 | 0 | 0 | 1 | 1 | 1 | 1 | 1 | 0 | 1 | 1 | 1 | 0 | 0 | 1 | 1 | 1 | 1 | 0 | 0 | 0 | 1 | 0 | 22 |
| **Matheson, 2024 (5)** | 1 | 1 | 0 | 1 | 1 | 1 | 1 | 1 | 0 | 1 | 1 | 1 | 0 | 0 | 0 | 0 | 1 | 1 | 1 | 1 | 1 | 0 | 1 | 1 | 1 | 0 | 0 | 1 | 1 | 1 | 1 | 0 | 0 | 1 | 0 | 0 | 23 |
| **Okafor, 2022 (6)** | 1 | 1 | 0 | 0 | 1 | 1 | 1 | 1 | 0 | 1 | 1 | 1 | 0 | 0 | 0 | 0 | 0 | 1 | 1 | 1 | 1 | 0 | 1 | 1 | 1 | 0 | 0 | 1 | 1 | 1 | 1 | 0 | 0 | 0 | 0 | 0 | 20 |
| **Majid, 2021 (7)** | 1 | 1 | 0 | 1 | 1 | 1 | 1 | 1 | 0 | 1 | 1 | 1 | 0 | 0 | 0 | 0 | 1 | 1 | 1 | 1 | 1 | 1 | 1 | 1 | 1 | 0 | 0 | 1 | 1 | 1 | 1 | 0 | 0 | 1 | 1 | 0 | 25 |
| **Kim, 2020 (8)** | 1 | 1 | 0 | 0 | 1 | 1 | 0 | 1 | 0 | 1 | 1 | 1 | 0 | 0 | 0 | 0 | 1 | 1 | 1 | 1 | 1 | 1 | 1 | 1 | 1 | 0 | 0 | 1 | 1 | 1 | 1 | 0 | 0 | 0 | 1 | 0 | 22 |
| **Badea, 2019 (9)** | 1 | 1 | 0 | 1 | 1 | 1 | 1 | 1 | 0 | 1 | 1 | 1 | 0 | 0 | 0 | 0 | 1 | 1 | 1 | 1 | 1 | 0 | 0 | 0 | 1 | 0 | 0 | 1 | 1 | 1 | 1 | 0 | 1 | 1 | 0 | 0 | 22 |

**Table S3: Certainty assessment of evidence for each outcome**

| **No of studies** | **Design** | **Risk of bias** | **Inconsistency** | **Indirectness^[1]^** | **Imprecision** | **Other^[2]^** | **Certainty**  **(overall score)^[3]^** |
| --- | --- | --- | --- | --- | --- | --- | --- |
| **Outcome:** Change in total cholesterol (TC) | | | | | | | |
| 4 | Observational (2) | No serious risk of bias | No serious inconsistency | No serious indirectness | No serious imprecision | Publication bias likely (-0.5) | Very Low  ⊕○○○ |
| **Outcome:** Change in serum high-density lipoprotein (HDL) | | | | | | | |
| 6 | Observational (2) | No serious risk of bias | Important Inconsistency (-0.5) | No serious indirectness | No serious imprecision | Adjusted estimate for confounders in two studies but remaining five studies did not | Very Low  ⊕○○○ |
| **Outcome:** Change In serum low-density lipoprotein (LDL) | | | | | | | |
| 4 | Observational (2) | No serious risk of bias | No serious inconsistency | No serious indirectness | No serious imprecision | Publication bias likely (-0.5) | Very Low  ⊕○○○ |
| **Outcome:** Change In serum very low-density lipoprotein (VLDL) | | | | | | | |
| 2 | Observational (2) | No serious risk of bias | No serious inconsistency | No serious indirectness | No serious imprecision | None | Very Low  ⊕○○○ |
| **Outcome:** Change in serum triglycerides (TG) | | | | | | | |
| 6 | Observational (2) | No serious risk of bias | No serious inconsistency | No serious indirectness | No serious imprecision | Adjusted estimate for confounders in two studies but remaining studies did not | Very Low  ⊕○○○ |

**Table S4: Summary of Findings (SoF)**

| **The Association Between E-cigarette use (Vaping) and Dyslipidemia** | | | |
| --- | --- | --- | --- |
| **People:** Individuals Aged above 18 years  **Settings:** Multiple Countries  **Intervention:** E-Ciggaretes (Vaping)  **Comparison:** Non-Vape Users | | | |
| **Outcomes** | **Findings** | **Number of studies** | **Certainty**  **of the evidence (GRADE) ^†^** |
| Change in total cholesterol (TC) | The overall weighted mean difference in serum total cholesterol showed an increase 3.66 mg/dL (95% CI: -13.89 to 21.20) that is attributed to vaping. | 4 | ⊕⊖⊖⊖ Very Low |
| **Change in serum high-density lipoprotein (HDL)** | Usage of e-ciggarets was associated with higher odds of having reduced serum HDL by 3.26 (95% CI: 2.28 to 4.65) compared to the non-vaping group. | 6 | ⊕⊖⊖⊖ Very Low |
| Change In serum low-density lipoprotein (LDL) | Serum LDL increased in users of vaping devices by 12.40 mg/dL (95% CI: 7.91 to 16.88) higher than non-users. | 4 | ⊕⊖⊖⊖ Very Low |
| **Change In serum very low-density lipoprotein (VLDL)** | Vaping was associated with a slightly increased serum VLDL by 3.23 mg/dL (95% CI: 1.43 to 5.02) comapred to non-users. | 2 | ⊕⊖⊖⊖ Very Low |
| **Change in serum triglycerides (TG)** | E-ciggarets significantly increased the odds of having an elevated serum triglycerides by 3.10 (95% CI: 1.18 to 8.16) when comapred to non-users. | 6 | ⊕⊖⊖⊖ Very Low |
| **^†^** GRADE Working Group grades of evidence  **High** = This research provides a very good indication of the likely effect. The likelihood that the effect will be substantially different^‡^ is low.  **Moderate** = This research provides a good indication of the likely effect. The likelihood that the effect will be substantially different^‡^ is moderate.  **Low** = This research provides some indication of the likely effect. However, the likelihood that it will be substantially different^‡^ is high.  **Very low** = This research does not provide a reliable indication of the likely effect. The likelihood that the effect will be substantially different^‡^ is very high.  ^‡^ Substantially different = a large enough difference that it might affect a decision | | | |

**Supplementary Figure S1: Quality assessment of included studies using the MASTER scale**

|  |  |  |  |  |  |  |  |
| --- | --- | --- | --- | --- | --- | --- | --- |
| Hasan,2024 | 4 | 4 | 3 | 6 | 4 | 3 | 3 |
| Lim,2024 | 3 | 3 | 3 | 5 | 4 | 3 | 1 |
| Matheson,2024 | 3 | 4 | 3 | 5 | 4 | 3 | 1 |
| Okafor,2022 | 2 | 4 | 3 | 4 | 4 | 3 | 0 |
| Majid,2021 | 3 | 4 | 3 | 6 | 4 | 3 | 2 |
| Kim,2020 | 2 | 3 | 3 | 6 | 4 | 3 | 1 |
| Badea,2019 | 3 | 4 | 3 | 5 | 2 | 3 | 2 |

*Equal Recruitment (pink) has 4 safeguards, Equal Retention (red) has 5 safeguards, Equal Ascertainment (orange) has 7 safeguards, Equal Implantation (yellow) has 6 safeguards, Equal Prognosis (blue) has 6 safeguards, Sufficient Analysis (green) has 3 safeguards, Temporal Precedence has 5 safeguards (grey).*

**Supplementary Figure S2: DOI plot assessing publication bias in the meta-analysis total cholesterol (TC) weighted mean differences (WMD).**

*The plot shows asymmetry suggestive of major publication bias (LFK index = +9.44).*

**Supplementary Figure S3: Forest plot showing a comparison of mean TC levels between vapers and non-vapers in leave one out analysis, leaving out Hasan, 2024.**

*Effect sizes are presented as weighted mean differences (WMD) in mg/dL with 95% confidence intervals (CI) using the Doi’s quality effects model (QE) and the random effects model (DL). Squares represent individual study estimates, with size proportional to study weight. The diamond indicates the overall estimate.*

**Supplementary Figure S4: DOI plot assessing publication bias in the meta-analysis of HDL weighted mean differences (WMD).**

*The plot shows asymmetry suggesting minor publication bias (LFK index = 1.41).*

**Supplementary Figure S5: DOI plot assessing publication bias in the meta-analysis of LDL weighted mean differences (WMD).**

*The plot shows asymmetry suggesting minor publication bias (LFK index = 1.05).*

**Supplementary Figure S6: Forest plot showing a comparison of mean LDL levels between vapers and non-vapers in leave one out analysis, leaving out Hasan, 2024.**

*Effect sizes are presented as weighted mean differences (WMD) with 95% confidence intervals (CI) using the Doi’s quality effects model and the random effects model. Squares represent individual study estimates, with size proportional to study weight. The diamond indicates the overall estimate.*

**Supplementary Figure S7: DOI plot assessing publication bias in the meta-analysis of TG weighted mean differences (WMD).**

*The plot shows asymmetry suggesting minor publication bias (LFK index = -1.34).*

1.	Haddaway NR, Collins AM, Coughlin D, Kirk S. The Role of Google Scholar in Evidence Reviews and Its Applicability to Grey Literature Searching. PLOS ONE. 2015;10(9):e0138237.

2.	Bramer WM, Rethlefsen ML, Kleijnen J, Franco OH. Optimal database combinations for literature searches in systematic reviews: a prospective exploratory study. Syst Rev. 2017;6(1):245.

3.	Hasan N, Nasser NA, Hussein AD, Mohsein OA. Effect of electronic smoking (Vaping) on thyroid hormones lEVEl and lipid profile in men. Ukrainian Biochemical Journal. 2024;96(5):55-64.

4.	Lim D, Choi JI, Kwon RJ, Lee SY, Cho YH, Park EJ, et al. Association between E-Cigarette Smoking and Insulin Resistance Using the Triglyceride-Glucose Index in Korean Adults: Korea National Health and Nutrition Examination Survey. Korean J Fam Med. 2024.

5.	Matheson C, Simovic T, Heefner A, Colon M, Tunon E, Cobb K, et al. Evidence of premature vascular dysfunction in young adults who regularly use e-cigarettes and the impact of usage length. Angiogenesis. 2024;27(2):229-43.

6.	Okafor CN, Okafor N, Kaliszewski C, Wang L. Association between electronic cigarette and combustible cigarette use with cardiometabolic risk biomarkers among U.S. adults. Ann Epidemiol. 2022;71:44-50.

7.	Majid S, Keith RJ, Fetterman JL, Weisbrod RM, Nystoriak J, Wilson T, et al. Lipid profiles in users of combustible and electronic cigarettes. Vasc Med. 2021;26(5):483-8.

8.	Kim T, Choi H, Kang J, Kim J. Association between electronic cigarette use and metabolic syndrome in the Korean general population: A nationwide population-based study. PLoS One. 2020;15(8):e0237983.

9.	Badea M, Gaman L, Delia C, Ilea A, Leașu F, Henríquez-Hernández LA, et al. Trends of Lipophilic, Antioxidant and Hematological Parameters Associated with Conventional and Electronic Smoking Habits in Middle-Age Romanians. J Clin Med. 2019;8(5).

1. Indirectness includes consideration of

   - Indirect (between study) comparisons
   - Indirect (surrogate) outcomes
   - Applicability (study populations, interventions or comparisons that are different than those of interest)
2. Other considerations for downgrading include publication bias. Other considerations for upgrading include a strong association with no plausible confounders, a dose response relationship, and if all plausible confounders or biases would decrease the size of the effect (if there is evidence of an effect), or increase it if there is evidence of no harmful effect (safety)
3. 4**High** = This research provides a very good indication of the likely effect. The likelihood that the effect will be substantially different** is low.

   3**Moderate** = This research provides a good indication of the likely effect. The likelihood that the effect will be substantially different** is moderate.

   2**Low** = This research provides some indication of the likely effect. However, the likelihood that it will be substantially different** is high.

   1**Very low** = This research does not provide a reliable indication of the likely effect. The likelihood that the effect will be substantially different** is very high.

   ** Substantially different = a large enough difference that it might affect a decision
