# Supplementary material for: The association between e-cigarette use and dyslipidemia: A systematic review and meta-analysis of observational studies
Source: PLoS One. 2026 Oct 26;21(10):e0361200. doi: 10.1371/journal.pone.0361200 (PMC13704818; doi:10.1371/journal.pone.0361200)
Supplement: Supplementary file 4 [file pone.0361200.s003.docx]

**The Association Between E-cigarette and Dyslipidemia: A Systematic Review and Meta-analysis**

Abstract

The abstract would benefit from greater clarity and completeness. Please report the total number of participants included across all studies, in addition to the number of studies. In addition, the reported *p*-value is potentially confusing, as it appears to refer to the heterogeneity test rather than the effect estimate. I suggest either explicitly labeling it as the *p*-value for heterogeneity or removing it from the abstract altogether, as heterogeneity *p*-values are uncommon in abstracts and may be misinterpreted by readers.

Data Sources and Search Terms

- Please write the search full string including Boolean operators, MeSH terms, truncation, and filters. Were they applied in all the databases. I recommend to show how the actual search done per database in the supplementary.

Screening of studies

- Screening of studies section appears to be redundant and confusing. Please clarify if a preliminary screening was done prior to full text screening. It is unclear and confusing because of the statement “Preliminarily included study records were retrieved in full text” and was followed by “Full texts of potentially eligible studies were then retrieved, and six reviewers, working in random three random pairs, independently assessed them.” Please clarify how many reviewers per stage of screening were involved (title and abstract screening / full text screening).
- I believe that all studies excluded after full screening should have a reason for exclusion and I would suggest removing the term if applicable.
- What is a random three random pairs mean? Please clarify.
- Please detail the software used as well as include in the citation. For example, EndNote (Version 21, Clarivate Analytics).

Eligibility Criteria:

- Please include the type of observational studies i.e. longitudinal (cohort), case-control, cross-sectional studies) were considered as this would affect the results on association.
- Please define what exclusive e-cigarette use. It may sound contradictory with the statement “who currently use e-cigarettes, regardless of their past vaping or smoking history.”
- Please clarify that self-reported dyslipidemia was excluded. Suggest to add “Only” in the statement “ Biochemically validated measures of dyslipidemia were accepted.” If that is the purpose of the statement.
- I noticed that the eligibility and outcomes sections do not specify the comparator group. Since the review aims to assess the association between exclusive e-cigarette use and lipid levels/dyslipidaemia, it is important to clarify what population the e-cigarette users were compared against (e.g., never-smokers, non-e-cigarette users, combustible cigarette smokers, or mixed controls). Without a clearly defined comparator, the direction and interpretation of the association may be ambiguous. Please specify the comparator criteria in the methods, and ensure consistency across eligibility criteria, data extraction, and statistical analyses. This clarification will strengthen the internal validity and reproducibility of the review.

Outcomes:

- Please explicitly state whether dyslipidaemia was defined by meeting any single lipid threshold or a combination of thresholds. Please mention source of your definition of dyslipidemia. In addition, present the primary (binary dyslipidaemia) and secondary (continuous lipid levels) outcomes more distinctly to avoid conceptual overlap. Ensure units and symbols are consistent (e.g., ≤, ≥) and specify whether thresholds were based on guideline sources. Overall, clearer wording will greatly improve interpretability.

Data Extraction:

- I have a concern about the data extraction approach. From the description “Mean levels of lipids and their standards deviations were extracted from studies which did not report adjusted effect sizes”, it appears that mean lipid levels and standard deviations were only extracted from studies that did not provide adjusted effect estimates. If this interpretation is correct, it implies that studies reporting effect measures (e.g., adjusted mean differences, ORs, β-coefficients) may not have contributed raw lipid levels to the secondary outcome analyses. This raises two issues that need clarification:
  - Were mean lipid levels and SDs extracted from studies that reported adjusted effect sizes whenever those data were available (either in the main text, tables, or supplementary files)? If not, this could lead to unnecessary exclusion of eligible data.
  - Would excluding such data influence the validity of the secondary meta-analysis? Selectively extracting continuous data only from a subset of studies may bias the pooled mean estimates or reduce statistical power.
- I recommend clarifying the extraction rule and, if applicable, justifying why studies that report effect measures could not contribute to the continuous outcome analysis, even when raw values were present. A sentence explaining how overlap between primary and secondary outcomes was handled would strengthen transparency.
- It would be useful to specify whether a standardized and piloted data extraction form was used in Excel, and whether inter-extractor agreement was assessed before full extraction began.
- I find the extraction section confusing and should be clarified. As written, “six authors independently extracted data in pairs” is contradictory, because extraction cannot be both independent and paired at the same time. Please specify whether each reviewer extracted data independently and was then paired for comparison, or whether each pair extracted jointly. It may be clearer to state: “data were extracted independently by pairs of reviewers, with discrepancies resolved through discussion or by a third reviewer.”
- Please clarify whether extraction was done independently and in duplicate for all data fields (study characteristics, outcomes, demographics) or only for effect estimates.
- I suggest to start of the with the study characteristics and followed by the outcomes.

Quality Assessment and Certainty

- It would help to briefly explain why the MASTER scale was selected rather than more commonly used tools for observational studies (e.g., Newcastle–Ottawa Scale or ROBINS-I). A short justification will assist readers who may be unfamiliar with MASTER.
- The sentence structure suggests that seven domains were assessed, but the manuscript lists only six types of bias. Please align terminology and ensure all domains are clearly named.
- Please clarify whether assessment was performed independently by multiple reviewers, how disagreements were resolved, and whether results were incorporated into the interpretation (e.g., sensitivity analysis, subgrouping by quality, or exclusion based on threshold scores).

Data Synthesis

- It is unclear why both the quality-effects and random-effects models were applied and which is considered the primary analysis—please clarify the rationale and interpretive hierarchy.
- It is not entirely clear whether studies that reported unadjusted odds ratios were excluded from the pooled OR analysis. The methods imply that only adjusted ORs were extracted and synthesized; if so, this could lead to the exclusion of eligible effect sizes and potential bias. Please clarify whether unadjusted ORs were pooled when adjusted estimates were unavailable, and explain the rationale either way.
- Please clarify the rationale for using several publication bias tools, specify which method provides the primary inference, and explain how discrepancies between methods were handled.

Results

To improve clarity and readability, I suggest adding a summary table in the main text that presents, for each lipid outcome, the pooled weighted mean difference (WMD) (with 95% CI), the pooled adjusted odds ratio where available, number of studies, total participants, heterogeneity (I²), model used (Quality adjusted/Random effects) and GRADE certainty. The detailed forest plots could then be moved to the Supplementary Materials.

“The seven included studies were published between 2019 and 2024, six were cross-sectional studies and one was a case-control study.” Please specify which studies were cross-sectional and which was case-control by citing the relevant references immediately after each study design.

“After meta-analysis, the overall weighted mean difference (WMD) showed a higher mean TC level in vapers, compared to non-vapers, of 3.66 mg/dL (95%CI -13.89 to 21.20) with a confidence interval still suggesting uncertainty.” - The interpretation of this result needs refinement. Although the point estimate suggests a higher mean TC level among vapers, the 95% CI crosses the null and is wide, indicating no statistically significant difference and substantial imprecision. The statement “with a confidence interval still suggesting uncertainty” is vague; please explicitly state that the result is not statistically significant and interpret it cautiously. Additionally, consider commenting on the clinical relevance of a 3.66 mg/dL difference, even if statistically significant, and ensure consistency between statistical inference and narrative conclusions.

“Given the small number of included studies (n = 5), estimates of heterogeneity (I²) and assessments of publication bias using Doi plots and the LFK index are likely unstable, and conclusions regarding unexplained heterogeneity or publication bias should therefore be interpreted with caution and more tentative language.

“Sensitivity analyses showed that the uncertainty significantly declined in the overall estimates, in leave-one out analyses upon leaving out Hasan, 2024 “ – The description of the sensitivity analysis requires clarification. Please specify the rationale for excluding Hasan (2024) in the leave-one-out analysis—was this study an outlier, at high risk of bias, methodologically different, or disproportionately influential? In addition, clarify the purpose of the sensitivity analysis (e.g., to assess robustness of pooled estimates, influence of individual studies, or impact of study quality). Without this justification, it is difficult to interpret the meaning of the reported reduction in uncertainty.

The term “strength of recommendation” is not appropriate in this context. GRADE is used here to assess the certainty (quality) of evidence, not the strength of a recommendation, which requires consideration of values, preferences, balance of benefits and harms, and resource use. Please revise the wording throughout to “certainty of evidence” (or “quality of evidence”) to accurately reflect the GRADE framework.

The statement on GRADE assessment needs clarification. Please specify the reasons for downgrading the certainty of evidence to *very low* (e.g., risk of bias, inconsistency, imprecision, indirectness, or publication bias), rather than only reporting the overall rating. Briefly summarizing the key domains driving the downgrading in the main text would improve transparency and align with GRADE reporting standards.

Figure 3 requires clearer outcome definition and terminology. The forest plot presents odds ratios, but it is unclear whether the outcome is low HDL, HDL dyslipidaemia (defined by a specific threshold), or another binary construct. Terms such as “high HDL” or “hyper-HDL” are not standard and should be avoided. Please specify the exact HDL cut-off used (e.g., HDL <1.0 mmol/L for men, <1.3 mmol/L for women), indicate the direction of effect (OR >1 reflects higher odds of low HDL), and revise the figure title and legend accordingly to ensure correct interpretation.

The handling of outlier studies and sensitivity analyses requires clarification. In the earlier analysis, Hasan (2024) was excluded in a leave-one-out sensitivity analysis, presumably because it was influential or an outlier; however, in the HDL analysis, Matheson appears to be an outlier but was not subjected to similar sensitivity testing. Please clarify the criteria used to identify influential or outlier studies and whether these criteria were applied consistently across outcomes. In addition, please state whether sensitivity analyses were pre-specified (a priori) in the protocol or conducted post-hoc in response to observed heterogeneity. Clear justification is necessary to avoid concerns about selective analysis.

“Since this study included observational studies, the GRADE certainty level starts as low and the GRADE was unchanged (low certainty) for the outcomes of TC, LDL and VLDL, but was upgraded to moderate in HDL and TG due to adjustment for estimated confounders. GRADE was then downgraded to low and very low due to high heterogeneity across the studies, possible risk of bias (low-quality cross-sectional studies), and uncertainty about publication bias.” - The description of the GRADE assessment is confusing and appears internally inconsistent. Earlier sections report very low certainty for HDL, yet this paragraph states that HDL (and TG) were upgraded to moderate certainty due to adjustment for confounders, before being downgraded again. Please reconcile these statements to ensure consistency across the manuscript. In addition, the rationale for upgrading is not sufficiently justified: while adjustment for confounders may support upgrading for residual confounding that would bias results toward the null, this is not appropriate when the underlying studies are at high risk of bias, particularly low-quality cross-sectional designs. GRADE upgrading criteria should be explicitly stated and applied cautiously; upgrading evidence that is already seriously limited by risk of bias is generally not recommended. I suggest clearly listing the specific upgrading and downgrading decisions for each outcome and revising the narrative to align with GRADE guidance.

**Discussion**

“This systematic review and meta-analysis revealed varying associations between e-cigarette use and serum lipid profiles. Overall, vaping was linked to higher total cholesterol (TC), LDL, VLDL, and triglyceride (TG) levels, and lower HDL levels, although the certainty of currently available evidence is very low. Adjusted odds ratios were only available for two lipids, and from only a few studies. For the rests of the lipids, only unadjusted mean differences were available for analysis.”- This paragraph appear overstated relative to the strength and consistency of the evidence. While the text states that vaping was “linked” to higher TC, LDL, VLDL, and TG and lower HDL levels, several of these associations—particularly TC—were not statistically significant, were based on very few studies (often n≈3–5), and relied largely on unadjusted mean differences. Only TG showed a statistically significant association based on adjusted effect estimates, whereas conclusions for other lipids are driven primarily by unadjusted analyses that are vulnerable to confounding. I recommend revising the language to clearly distinguish between adjusted and unadjusted findings, avoid causal or definitive phrasing (“linked”), and explicitly acknowledge that the observed mean differences are small, imprecise, and of uncertain clinical significance. These limitations should also be reflected consistently in the GRADE assessments and overall conclusions.

**Conclusion**

The conclusion should be revised to more accurately reflect the substantial limitations of the evidence and avoid overstating the findings. Phrases such as “vaping is likely associated with deranged lipid levels” imply a degree of certainty that is not supported by the data, given that most analyses were based on unadjusted observational studies, several associations were not statistically significant, heterogeneity was high, and overall GRADE certainty was low or very low. I recommend using more cautious, descriptive language (e.g., “limited and low-certainty evidence suggests possible differences in lipid levels, particularly triglycerides”) and explicitly noting the uncertainty and lack of causal inference. Aligning the conclusion more closely with the stated limitations will improve internal consistency and methodological rigor.
