# Supplementary material for: The association between e-cigarette use and dyslipidemia: A systematic review and meta-analysis of observational studies
Source: PLoS One. 2026 Oct 26;21(10):e0361200. doi: 10.1371/journal.pone.0361200 (PMC13704818; doi:10.1371/journal.pone.0361200)
Supplement: Supplementary file 5 [file pone.0361200.s006.docx]

**Response to Reviewer Comments**

We sincerely thank the reviewer for their thoughtful, detailed, and constructive feedback. Their comments have substantially strengthened the methodological transparency, accuracy, and interpretive balance of our manuscript. Below, we provide a point-by-point response to each comment, with all changes incorporated into the revised manuscript.

**Abstract**

***Comment****:* The abstract would benefit from greater clarity and completeness. Please report the total number of participants included across all studies, in addition to the number of studies. In addition, the reported *p*-value is potentially confusing, as it appears to refer to the heterogeneity test rather than the effect estimate. I suggest either explicitly labeling it as the *p*-value for heterogeneity or removing it from the abstract altogether, as heterogeneity *p*-values are uncommon in abstracts and may be misinterpreted by readers.

***Response****:* We thank the reviewer for this suggestion. The abstract now includes the total number of participants across all included studies (n = 25,690). The p-value for heterogeneity has been removed to avoid potential misinterpretation, as it referred to statistical heterogeneity rather than the effect estimate.

***Additional changes as suggested by other reviewers:***

1. **Title:** Added explicit designation of study design. Title now reads: *"Association Between E‑Cigarette Use and Dyslipidemia: A Systematic Review and Meta‑Analysis of Observational Studies."*
2. **Abstract Methods:** Clarified why subgroup analyses (age/sex) were not feasible and explicitly stated *"due to insufficient stratified reporting in included studies."*. Labeled as "unadjusted" weighted mean differences to prevent misinterpretation as causal/adjusted effects.
3. **Abstract Results:**
   - All continuous lipid outcomes (TC, LDL, HDL, TG) now explicitly labeled as **"unadjusted"** weighted mean differences to prevent misinterpretation as causal/adjusted effects.
   - Non‑significant TC finding softened to *"non‑significant trend toward higher total cholesterol"* to reflect the confidence interval crossing the null value.
4. **Abstract Conclusion:** Tempered causal language to match GRADE certainty ratings. Revised to *"* *This synthesis suggests that vaping may be associated with dyslipidemia. However, the certainty of evidence is low to very low quality, precluding causal inferences. Further robust longitudinal studies are needed to explore these associations."*

**Introduction**

***Additional changes as suggested by other reviewers:***

The Introduction has been revised as follows:

• Text has been added to explain the clinical relevance of dyslipidemia in this context, noting that sustained lipid perturbations can accelerate atherosclerotic cardiovascular disease (ASCVD) pathogenesis: "potentially translating into long-term cardiovascular risk even among younger cohorts."

• A definition of "exclusive vaping" has been inserted to clarify that the review minimizes confounding from dual use: "focus on 'exclusive vaping'—defined here as the use of e-cigarettes without concurrent use of combustible tobacco products."

**Data Sources and Search Terms**

***Comment****:* Please write the search full string including Boolean operators, MeSH terms, truncation, and filters. Were they applied in all the databases. I recommend to show how the actual search done per database in the supplementary.

***Response****:* We have added the complete search strategy for each database (PubMed, Cochrane CENTRAL, CINAHL, Embase, Scopus) as under the search strategy in the supplementary file. We included all MeSH/Emtree terms, free-text keywords, Boolean operators, truncation symbols, and applied filters.

***Additional changes as suggested by other reviewers:***

- We also included the search strings and the methods used to search Google Scholar, abiding by the best recommended practices in research methodology.

**Screening of Studies**

***Comment****:* Screening of studies section appears to be redundant and confusing. Please clarify if a preliminary screening was done prior to full text screening. It is unclear and confusing because of the statement “Preliminarily included study records were retrieved in full text” and was followed by “Full texts of potentially eligible studies were then retrieved, and six reviewers, working in random three random pairs, independently assessed them.” Please clarify how many reviewers per stage of screening were involved (title and abstract screening / full text screening).
***Comment****:* I believe that all studies excluded after full screening should have a reason for exclusion and I would suggest removing the term if applicable.
***Comment****:* What is a random three random pairs mean? Please clarify.
***Comment****:* Please detail the software used as well as include in the citation. For example, EndNote (Version 21, Clarivate Analytics).

***Response****:* We appreciate this helpful clarification request. The Methods section has been revised to state explicitly that:

- Title/abstract screening was performed independently by six reviewers working in three pairs (two reviewers per record) using Rayyan (Rayyan.ai, 2023). Full-text assessment was also conducted independently by the same six reviewers in three pairs that are different from original three pairs matches as an attempt to minimize reviewer bias. Discrepancies at either stage were resolved by discussion or adjudication by a senior reviewer. This section was indeed redundant and was revised well to for better readability and clarity.
- The phrase "where applicable" regarding exclusion reasons has been removed; all exclusions after full-text screening did indeed include explicit reasons mentioned in the PRISMA diagram.
- EndNote (Version 21, Clarivate Analytics) is now cited as the reference management software.

**Eligibility Criteria**

***Comment****:* Please include the type of observational studies i.e. longitudinal (cohort), case-control, cross-sectional studies) were considered as this would affect the results on association.
***Comment****:* Please define what exclusive e-cigarette use. It may sound contradictory with the statement “who currently use e-cigarettes, regardless of their past vaping or smoking history.”
***Comment****:* clarify that self-reported dyslipidemia was excluded. Suggest to add “Only” in the statement “ Biochemically validated measures of dyslipidemia were accepted.” If that is the purpose of the statement.
***Comment****:* I noticed that the eligibility and outcomes sections do not specify the comparator group. Since the review aims to assess the association between exclusive e-cigarette use and lipid levels/dyslipidaemia, it is important to clarify what population the e-cigarette users were compared against (e.g., never-smokers, non-e-cigarette users, combustible cigarette smokers, or mixed controls). Without a clearly defined comparator, the direction and interpretation of the association may be ambiguous. Please specify the comparator criteria in the methods, and ensure consistency across eligibility criteria, data extraction, and statistical analyses. This clarification will strengthen the internal validity and reproducibility of the review.

***Response****:* Thank you for highlighting these important points. We have revised the eligibility criteria to:

- Explicitly state inclusion of any observational study design (cross-sectional, case–control, and cohort studies).
- Define "exclusive e-cigarette users" as individuals reporting current e-cigarette use regardless of past smoking history, with no concurrent combustible tobacco use at the time of assessment.
- Clarify that *only* biochemically measured lipid levels were accepted; self-reported dyslipidemia diagnoses were excluded.
- Specify the comparator as "non-smokers" (individuals reporting no current use of e-cigarettes or combustible tobacco), as consistently defined across included studies.

**Outcomes**

***Comment****:* Please explicitly state whether dyslipidaemia was defined by meeting any single lipid threshold or a combination of thresholds. Please mention source of your definition of dyslipidemia. In addition, present the primary (binary dyslipidaemia) and secondary (continuous lipid levels) outcomes more distinctly to avoid conceptual overlap. Ensure units and symbols are consistent (e.g., ≤, ≥) and specify whether thresholds were based on guideline sources. Overall, clearer wording will greatly improve interpretability.

***Response****:* We have revised the Outcomes section to state that primary outcome was dyslipidemia, and defined as meeting *any* of the following thresholds based on the National Cholesterol Education Program Adult Treatment Panel III (NCEP ATP III) : TC ≥200 mg/dL (≥5.2 mmol/L), LDL C ≥130 mg/dL (≥3.4 mmol/L), HDL C <40 mg/dL (<1.0 mmol/L) in men or <50 mg/dL (<1.3 mmol/L) in women, and TG ≥150 mg/dL (≥1.7 mmol/L). Threshold sources are now cited in text. Secondary outcomes included a comparison of continuous lipid levels (mg/dL).

**Data Extraction**

***Comment****:* I have a concern about the data extraction approach. From the description “Mean levels of lipids and their standards deviations were extracted from studies which did not report adjusted effect sizes”, it appears that mean lipid levels and standard deviations were only extracted from studies that did not provide adjusted effect estimates. If this interpretation is correct, it implies that studies reporting effect measures (e.g., adjusted mean differences, ORs, β-coefficients) may not have contributed raw lipid levels to the secondary outcome analyses. This raises two issues that need clarification:

- Were mean lipid levels and SDs extracted from studies that reported adjusted effect sizes whenever those data were available (either in the main text, tables, or supplementary files)? If not, this could lead to unnecessary exclusion of eligible data.
- Would excluding such data influence the validity of the secondary meta-analysis? Selectively extracting continuous data only from a subset of studies may bias the pooled mean estimates or reduce statistical power.

***Comment****:* I recommend clarifying the extraction rule and, if applicable, justifying why studies that report effect measures could not contribute to the continuous outcome analysis, even when raw values were present. A sentence explaining how overlap between primary and secondary outcomes was handled would strengthen transparency.
***Comment****:* It would be useful to specify whether a standardized and piloted data extraction form was used in Excel, and whether inter-extractor agreement was assessed before full extraction began.
***Comment****:* I find the extraction section confusing and should be clarified. As written, “six authors independently extracted data in pairs” is contradictory, because extraction cannot be both independent and paired at the same time. Please specify whether each reviewer extracted data independently and was then paired for comparison, or whether each pair extracted jointly. It may be clearer to state: “data were extracted independently by pairs of reviewers, with discrepancies resolved through discussion or by a third reviewer.”
***Comment****:* Please clarify whether extraction was done independently and in duplicate for all data fields (study characteristics, outcomes, demographics) or only for effect estimates.
***Comment****:* I suggest to start of the with the study characteristics and followed by the outcomes.

***Response****:* We thank the reviewer for identifying these ambiguities. The Data Extraction section has been comprehensively revised and reworked to state that:

- Mean lipid levels and SDs were extracted from *all* studies whenever available, regardless of whether adjusted effect estimates were reported, to enable secondary analyses. Studies contributed to both primary (adjusted effects) and secondary (raw means) analyses when both data types were present.
- Six reviewers worked in three pairs; within each pair, reviewers independently extracted *all* data fields (study characteristics, demographics, outcomes) using a standardized Excel form. Discrepancies were resolved by discussion or third-reviewer adjudication. This was similar to the distribution in the screening processes mentioned above.
- The narrative now presents study characteristics before outcomes for improved logical flow.

**Quality Assessment and Certainty**

***Comment:*** It would help to briefly explain why the MASTER scale was selected rather than more commonly used tools for observational studies (e.g., Newcastle–Ottawa Scale or ROBINS-I). A short justification will assist readers who may be unfamiliar with MASTER.
***Comment:*** The sentence structure suggests that seven domains were assessed, but the manuscript lists only six types of bias. Please align terminology and ensure all domains are clearly named.
***Comment:*** Please clarify whether assessment was performed independently by multiple reviewers, how disagreements were resolved, and whether results were incorporated into the interpretation (e.g., sensitivity analysis, subgrouping by quality, or exclusion based on threshold scores).

***Response****:* This section also needed a comprehensive revision to allow better readability and for a more clear and transparent approach. We have added a brief justification for selecting the MASTER scale: it provides a comprehensive, numerical assessment of methodological rigor across observational epidemiological studies, evaluating not only bias but also design, analysis, and interpretation quality, making it well-suited for this review. All seven MASTER domains are now explicitly named (Equal Recruitment, Retention, Ascertainment, Implementation, Prognosis, Sufficient Analysis, Temporal Precedence). We clarify that the same approach discussed in screening and data extraction was applied to quality assessment. Studies were divided into 3 thirds with and each third of studies was assessed independently by a pair of reviewers, with each reviewer assessing all studies then comparing with their pair. Disagreements were resolved by a senior reviewer. Quality scores were incorporated into the quality-effects model weighting but did not trigger study exclusion.

**Data Synthesis**

***Comment****:* It is unclear why both the quality-effects and random-effects models were applied and which is considered the primary analysis—please clarify the rationale and interpretive hierarchy.
***Comment****:* It is not entirely clear whether studies that reported unadjusted odds ratios were excluded from the pooled OR analysis. The methods imply that only adjusted ORs were extracted and synthesized; if so, this could lead to the exclusion of eligible effect sizes and potential bias. Please clarify whether unadjusted ORs were pooled when adjusted estimates were unavailable, and explain the rationale either way.
***Comment****:* Please clarify the rationale for using several publication bias tools, specify which method provides the primary inference, and explain how discrepancies between methods were handled.

***Response****:* Thank you so much for bringing our attention to the original Data synthesis section’s need for clarity. We have comprehensively revised this section and added the needed details for the section to state that:

- The quality-effects model was the *primary* analysis; the random-effects model served as a *sensitivity analysis* to assess robustness and enable comparison with other meta-analyses, as the random effects meta-analysis is the most commonly used method.
- A three-tier hierarchy guided effect estimate selection: (1) adjusted estimates prioritized; (2) unadjusted estimates pooled when adjusted data were unavailable; (3) raw means/SDs used only when neither adjusted nor unadjusted effects were reported.
- Leave-one-out sensitivity analyses were performed *post-hoc* for all outcomes; a study was deemed influential if its removal reduced *I*² by ≥20 percentage points or narrowed the 95% CI width by ≥30%.
- Publication bias was assessed using Doi plots (visual) and the LFK index (quantitative); the Doi plots provided the primary inference, with LFK index (>2 or <−2 indicating major asymmetry) as complementary assessment.

**Results**

***Comment:*** To improve clarity and readability, I suggest adding a summary table in the main text that presents, for each lipid outcome, the pooled weighted mean difference (WMD) (with 95% CI), the pooled adjusted odds ratio where available, number of studies, total participants, heterogeneity (I²), model used (Quality adjusted/Random effects) and GRADE certainty. The detailed forest plots could then be moved to the Supplementary Materials.
***Comment:*** “The seven included studies were published between 2019 and 2024, six were cross-sectional studies and one was a case-control study.” Please specify which studies were cross-sectional and which was case-control by citing the relevant references immediately after each study design.
***Comment:*** “After meta-analysis, the overall weighted mean difference (WMD) showed a higher mean TC level in vapers, compared to non-vapers, of 3.66 mg/dL (95%CI -13.89 to 21.20) with a confidence interval still suggesting uncertainty.” - The interpretation of this result needs refinement. Although the point estimate suggests a higher mean TC level among vapers, the 95% CI crosses the null and is wide, indicating no statistically significant difference and substantial imprecision. The statement “with a confidence interval still suggesting uncertainty” is vague; please explicitly state that the result is not statistically significant and interpret it cautiously. Additionally, consider commenting on the clinical relevance of a 3.66 mg/dL difference, even if statistically significant, and ensure consistency between statistical inference and narrative conclusions.
***Comment:*** “Given the small number of included studies (n = 5), estimates of heterogeneity (I²) and assessments of publication bias using Doi plots and the LFK index are likely unstable, and conclusions regarding unexplained heterogeneity or publication bias should therefore be interpreted with caution and more tentative language.
***Comment:*** “Sensitivity analyses showed that the uncertainty significantly declined in the overall estimates, in leave-one out analyses upon leaving out Hasan, 2024 “ – The description of the sensitivity analysis requires clarification. Please specify the rationale for excluding Hasan (2024) in the leave-one-out analysis—was this study an outlier, at high risk of bias, methodologically different, or disproportionately influential? In addition, clarify the purpose of the sensitivity analysis (e.g., to assess robustness of pooled estimates, influence of individual studies, or impact of study quality). Without this justification, it is difficult to interpret the meaning of the reported reduction in uncertainty.
***Comment:*** The term “strength of recommendation” is not appropriate in this context. GRADE is used here to assess the certainty (quality) of evidence, not the strength of a recommendation, which requires consideration of values, preferences, balance of benefits and harms, and resource use. Please revise the wording throughout to “certainty of evidence” (or “quality of evidence”) to accurately reflect the GRADE framework.
***Comment:*** The statement on GRADE assessment needs clarification. Please specify the reasons for downgrading the certainty of evidence to *very low* (e.g., risk of bias, inconsistency, imprecision, indirectness, or publication bias), rather than only reporting the overall rating. Briefly summarizing the key domains driving the downgrading in the main text would improve transparency and align with GRADE reporting standards.
***Comment:*** Figure 3 requires clearer outcome definition and terminology. The forest plot presents odds ratios, but it is unclear whether the outcome is low HDL, HDL dyslipidaemia (defined by a specific threshold), or another binary construct. Terms such as “high HDL” or “hyper-HDL” are not standard and should be avoided. Please specify the exact HDL cut-off used (e.g., HDL <1.0 mmol/L for men, <1.3 mmol/L for women), indicate the direction of effect (OR >1 reflects higher odds of low HDL), and revise the figure title and legend accordingly to ensure correct interpretation.
***Comment:*** The handling of outlier studies and sensitivity analyses requires clarification. In the earlier analysis, Hasan (2024) was excluded in a leave-one-out sensitivity analysis, presumably because it was influential or an outlier; however, in the HDL analysis, Matheson appears to be an outlier but was not subjected to similar sensitivity testing. Please clarify the criteria used to identify influential or outlier studies and whether these criteria were applied consistently across outcomes. In addition, please state whether sensitivity analyses were pre-specified (a priori) in the protocol or conducted post-hoc in response to observed heterogeneity. Clear justification is necessary to avoid concerns about selective analysis.
***Comment:*** “Since this study included observational studies, the GRADE certainty level starts as low and the GRADE was unchanged (low certainty) for the outcomes of TC, LDL and VLDL, but was upgraded to moderate in HDL and TG due to adjustment for estimated confounders. GRADE was then downgraded to low and very low due to high heterogeneity across the studies, possible risk of bias (low-quality cross-sectional studies), and uncertainty about publication bias.” - The description of the GRADE assessment is confusing and appears internally inconsistent. Earlier sections report very low certainty for HDL, yet this paragraph states that HDL (and TG) were upgraded to moderate certainty due to adjustment for confounders, before being downgraded again. Please reconcile these statements to ensure consistency across the manuscript. In addition, the rationale for upgrading is not sufficiently justified: while adjustment for confounders may support upgrading for residual confounding that would bias results toward the null, this is not appropriate when the underlying studies are at high risk of bias, particularly low-quality cross-sectional designs. GRADE upgrading criteria should be explicitly stated and applied cautiously; upgrading evidence that is already seriously limited by risk of bias is generally not recommended. I suggest clearly listing the specific upgrading and downgrading decisions for each outcome and revising the narrative to align with GRADE guidance.

***Response****:* We have implemented all these improvements:

- Added Table 2 summarizing pooled estimates, participants, heterogeneity, models, and GRADE certainty for each lipid outcome.
- Specified and cited the six studies were cross-sectional (Lim 2024, Matheson 2024, Okafor 2022, Majid 2021, Kim 2020, Badea 2019) and one was case–control (Hasan 2024).
- Changes made to section 3.4 “Association between vaping and levels of TC”:
  - Clarified statistical interpretation and added clinical context directly within the Results section:
    - "The overall weighted mean difference (WMD) was 3.66 mg/dL higher in vapers compared to non-vapers (95% CI −13.89 to 21.20). However, the confidence interval crossed the null value (zero), indicating no statistically significant difference. Even if statistically significant, a mean difference of 3.66 mg/dL would likely be of minimal clinical relevance for cardiovascular risk assessment."
  - Added methodological caveats regarding heterogeneity and bias metrics with sparse data:
    - "However, with only five studies included, both the magnitude of heterogeneity and the bias assessment should be interpreted cautiously, and potential sources of heterogeneity could not be explored due to insufficient data for meaningful subgroup or meta-regression analyses."
  - Explicitly justified the sensitivity analysis for Hasan 2024:
    - "Leave-one-out sensitivity analyses showed that heterogeneity substantially decreased (I² reduced from 86.9% to 0%) and the confidence interval narrowed upon exclusion of Hasan 2024, the only case-control study, conducted exclusively in Iraqi men only, indicating disproportionate influence on the pooled estimate."
- Replaced all instances of "strength of recommendation" with "certainty of evidence" or “quality of evidence” throughout the manuscript.
- Revised Section 3.9 to transparently report GRADE ratings per outcome with explicit reasons for downgrading (e.g., TC and LDL downgraded to very low due to suspected publication bias; VLDL rated very low due to only two studies).
- Updated Figures 3 and 6 to label outcomes as "HDL dyslipidemia" and "TG dyslipidemia," with NCEP ATP III threshold definitions provided in figure footnotes.

***Additional changes as suggested by other reviewers:***

- To improve transparency regarding the adjusted analyses for HDL and TG, we have added the specific confounders adjusted for in each study directly into the **Figure Legends**. We have carefully revised the wording for LDL and VLDL findings to ensure clinical recommendations are cautious and reflect the limitations of unadjusted data.

**Discussion**

***Comment****:* “This systematic review and meta-analysis revealed varying associations between e-cigarette use and serum lipid profiles. Overall, vaping was linked to higher total cholesterol (TC), LDL, VLDL, and triglyceride (TG) levels, and lower HDL levels, although the certainty of currently available evidence is very low. Adjusted odds ratios were only available for two lipids, and from only a few studies. For the rests of the lipids, only unadjusted mean differences were available for analysis.”- This paragraph appear overstated relative to the strength and consistency of the evidence. While the text states that vaping was “linked” to higher TC, LDL, VLDL, and TG and lower HDL levels, several of these associations—particularly TC—were not statistically significant, were based on very few studies (often n≈3–5), and relied largely on unadjusted mean differences. Only TG showed a statistically significant association based on adjusted effect estimates, whereas conclusions for other lipids are driven primarily by unadjusted analyses that are vulnerable to confounding. I recommend revising the language to clearly distinguish between adjusted and unadjusted findings, avoid causal or definitive phrasing (“linked”), and explicitly acknowledge that the observed mean differences are small, imprecise, and of uncertain clinical significance. These limitations should also be reflected consistently in the GRADE assessments and overall conclusions.

***Response****:* We have revised the opening paragraph of the Discussion to accurately reflects statistical significance, adjustment status, and GRADE ratings. It now reads “This systematic review and meta-analysis revealed varying associations between e-cigarette use and serum lipid profiles. Overall, unadjusted analysis suggested higher mean levels of TC, LDL, VLDL, and triglycerides and lower HDL in vapers compared with non-vapers; total cholesterol showed a non-significant mean difference and the certainty of evidence was very low. Adjusted analyses, available only for HDL and triglycerides, indicated a stronger association with dyslipidemia patterns - specifically lower HDL and higher triglycerides - and the certainty of evidence was low.”.

***Additional changes as suggested by other reviewers:***

We have revised the text to strictly adhere to observational language, distinguish between mechanism and exposure, and explicitly address the "former smoker" and "health selection" phenomena as major interpretative factors. The following changes have been made:

- **Causal Language**: We have systematically replaced causal terms (e.g., "effect," "impact," "leads to") with observational terminology throughout the Discussion and Conclusion.
  - *Revised Text:* "This systematic review and meta-analysis **identified** varying associations..." and "Adjusted analyses... indicated a stronger **independent** association..."
  - *Revised Text:* "Though marketed as safer alternatives, **observational evidence suggests** e-cigarettes may negatively impact lipid metabolism..."
- **Exposure Profiles vs. Mechanisms:** We added a specific distinction between the shared mechanism (nicotine) and the differing exposure profiles (combustion products) to avoid over-reliance on the cigarette analogy.
  - *Inserted Text:* "While e-cigarettes share the delivery of nicotine with these traditional products, it is important to note that the exposure profiles differ significantly; specifically, e-cigarettes lack the combustion-generated carbon monoxide and tar found in conventional cigarettes ..."
- **Smoking History & Residual Confounding:** We expanded the interpretation of the HDL and LDL findings to explicitly cite the lingering metabolic effects of past smoking as a primary alternative explanation, rather than relegating this solely to the limitations section.
  - *Inserted Text:* "However, in interpreting these findings for e-cigarettes, the potential for residual confounding by past smoking history is substantial. Many e-cigarette users are former combustible cigarette smokers, and the observed lower HDL may partially reflect the lingering metabolic effects of past smoking rather than vaping alone ..."
- **Reverse Causality / Health Selection:** We incorporated the concept of "sick switchers" to address the potential for reverse causality.
  - *Inserted Text:* "Furthermore, 'health selection' bias cannot be ruled out, wherein individuals with pre-existing health concerns or dyslipidemia switch from cigarettes to vaping to mitigate harm, thereby clustering higher-risk lipid profiles within the vaping group ..."

**Conclusion**

***Comment****:* The conclusion should be revised to more accurately reflect the substantial limitations of the evidence and avoid overstating the findings. Phrases such as “vaping is likely associated with deranged lipid levels” imply a degree of certainty that is not supported by the data, given that most analyses were based on unadjusted observational studies, several associations were not statistically significant, heterogeneity was high, and overall GRADE certainty was low or very low. I recommend using more cautious, descriptive language (e.g., “limited and low-certainty evidence suggests possible differences in lipid levels, particularly triglycerides”) and explicitly noting the uncertainty and lack of causal inference. Aligning the conclusion more closely with the stated limitations will improve internal consistency and methodological rigor.

***Response****:* The Conclusion has been revised to provide a transparent reflection of the study’s findings and their limitations without providing a false overstating of the conclusions. It now reads “This synthesis suggests possible differences in lipid levels among vapers compared with non-vapers. However, these findings are based on findings from a combination of adjusted and unadjusted analyses from observational studies. Several associations had substantial heterogeneity, and GRADE certainty ranged from very low to low. Consequently, the evidence does not support firm conclusions about an association - or its direction - between vaping and lipid profiles. Further robust longitudinal studies with adjustment for key confounders are needed to clarify these associations with greater certainty.”.

We are so grateful for the opportunity to improve our manuscript through this rigorous review. All changes have been implemented carefully, and we believe the revised version is substantially strengthened in transparency, accuracy, and interpretive balance of our manuscript. Thank you again for your valuable contribution to our work.

**Response to Reviewer Comments**

Dear Dr Regencia,

We sincerely thank you so much for your thoughtful, detailed, and constructive feedback. Your comments have substantially strengthened the methodological transparency, accuracy, and interpretive balance of our manuscript. Below we provide a point-by-point response to each comment, with all changes implemented in the revised manuscript.

**Abstract**

**1) Overall / general impressions**

***Comment****:* This is a timely question and the manuscript is clearly structured (PRISMA, PROSPERO registration, GRADE). The narrative is generally easy to follow, and the authors are transparent that most evidence is low certainty and largely observational.

***Comment****:* That said, the current evidence base is thin (7 studies; mostly cross-sectional), and the manuscript would benefit from tightening claims and strengthening how you handle confounding, “exclusive vaping” definitions, and the mixing of *adjusted binary outcomes* with *unadjusted continuous outcomes*. This is the biggest interpretability issue for readers.

***Response****:* Thank you for your comment. We have revised the manuscript to now have softer language, better readability, and better flow of ideas.

**2) Title / Abstract**

***Comment*:** **Title**: Clear and appropriate. Consider clarifying in the title (or subtitle) that the included evidence is observational (since causality cannot be inferred).

***Comment***: **Abstract (Methods**): You mention subgroup analyses (age/sex) but note they weren't possible. That's fine, but I'd recommend explicitly stating why (e.g., "insufficient stratified reporting") to pre-empt reviewer concerns.

***Comment***: **Abstract (Results**):

- The reporting mixes adjusted odds ratios (aORs) for dyslipidemia outcomes with weighted mean differences from unadjusted means. I’d strongly suggest explicitly labeling the WMD results as “unadjusted” in the abstract to avoid readers interpreting them as causal/adjusted effects.
- The TC result is reported as borderline (CI crosses 0). You might soften wording (“trend toward higher TC”) and keep emphasis proportional to certainty.

***Comment***: **Abstract (Conclusion**): The phrase "vaping is likely associated" is a bit strong given "very low–low" certainty; consider "may be associated" or "is associated in limited observational data."

***Response****:* We appreciate these valuable suggestions to enhance precision and align language with the observational nature and low certainty of the evidence base. The Title and Abstract have been revised as follows:

• The title has been updated to explicitly state the observational design: "Association Between E‑Cigarette Use and Dyslipidemia: A Systematic Review and Meta‑Analysis of Observational Studies."

• The Abstract Methods section now explicitly states why subgroup analyses were not feasible: "Subgroup analyses by age and sex were planned a priori but could not be performed due to insufficient stratified reporting in the included studies."

• All continuous lipid outcomes in the Abstract Methods and Results are now explicitly labeled as unadjusted.

• The interpretation of the non‑significant TC finding has been softened to reflect the confidence interval crossing the null value: "showed a non‑significant trend toward higher total cholesterol"

• The Abstract Conclusion has been tempered to align with GRADE certainty ratings: "Current observational evidence suggests e‑cigarette use may be associated with dyslipidemia; however, the certainty of evidence is very low to low, precluding causal inference."

***Additional changes as suggested by other reviewers:***

• The abstract now includes the total number of participants across all included studies (n = 25,690).

• The p-value for heterogeneity has been removed to avoid potential misinterpretation, as it referred to statistical heterogeneity rather than the effect estimate.

**3) Introduction**
***Comment***: The rationale is strong: you connect ENDS uptake, cardiovascular risk, and dyslipidemia as a plausible mechanism.
***Comment***: Clarify why dyslipidemia matters clinically in this context—e.g., whether small shifts in LDL/HDL in young adults could translate into long-term ASCVD risk (even if speculative). Keep it balanced and not alarmist.
***Comment***: Pre-define what you mean by "exclusive vaping" in the introduction (because readers will immediately worry about former smoking/dual use).

***Response****:* We appreciate the positive assessment of our rationale and the helpful suggestions to clarify clinical significance and definitions. The Introduction has been revised as follows:

• Text has been added to explain the clinical relevance of dyslipidemia in this context, noting that sustained lipid perturbations can accelerate atherosclerotic cardiovascular disease (ASCVD) pathogenesis: "potentially translating into long-term cardiovascular risk even among younger cohorts."

• A definition of "exclusive vaping" has been inserted to clarify that the review minimizes confounding from dual use: "focus on 'exclusive vaping'—defined here as the use of e-cigarettes without concurrent use of combustible tobacco products."

**4) Methods**
***Comment***: **4.1 Study design / protocol**: PRISMA 2020 + PROSPERO registration are clear strengths.

- Briefly state any deviations from the registered protocol (if none, explicitly say "no deviations"). Reviewers often look for this.

***Comment*: 4.2 Search strategy**: You searched major databases plus Google Scholar and updated to May 31, 2025—excellent coverage.

- Please report whether you applied any Google Scholar screening limits (e.g., first N pages/results) to ensure reproducibility.
- Consider adding the full search strings (not just "keywords") in the supplement if not already there.

***Comment*: 4.3 Eligibility criteria:** You exclude dual users but allow participants "regardless of past vaping or smoking history." This opens a major confounding pathway: many "exclusive vapers" are former smokers, and prior smoking could drive lipid differences.

- Either restrict to never-smoker vapers (if data allow),
- or pre-specify a subgroup/sensitivity analysis comparing "exclusive vapers who never smoked" vs "exclusive vapers who are former smokers,"
- or at minimum clearly describe this as a key limitation and reflect it in GRADE downgrading decisions.

***Comment*: 4.4 Outcomes**: The dyslipidemia cutoffs are provided—helpful.

- Specify whether you accepted each study's own dyslipidemia definitions if different, and how you harmonized them (or whether you did not). Outcome definition variability can create heterogeneity.

***Comment*: 4.5 Data extraction**: The paired extraction process is reassuring.

- Add a short list of "core confounders" you attempted to extract (age, sex, BMI, smoking history, alcohol, diet, activity, socioeconomic status) to help readers assess residual confounding.

***Comment*: 4.6 Risk of bias / quality (MASTER**): Using MASTER is interesting and defensible, but many readers are more familiar with NOS/ROBINS-I. Add 1–2 sentences justifying why MASTER was chosen and how to interpret its scores.
***Comment*: 4.7 Synthesis / statistics**: You use a quality-effects (inverse heterogeneity) model and also random effects for comparison—that's thoughtful.

- Mixing adjusted ORs (binary outcomes) with unadjusted mean differences (continuous outcomes) creates an "apples vs oranges" feel. Consider reorganizing Results so it is crystal-clear which outcomes are adjusted vs unadjusted, and avoid implying they carry similar evidential weight.
- For very high heterogeneity (e.g., TG I² ~99%), consider whether pooling is meaningful; at minimum, add interpretive caution ("summary effect may not represent a common effect").

***Response****:* We acknowledge that the methods section required some changes, and we appreciate your assistance to make this section comprehensive, detailed, organized, and transparent. We have carefully revised the methods section to state the following:

- Added a statement about deviation from PROSPERO protocol and mention explicitly that there were no major deviations from protocol. Subgroup analyses could not be conducted because of the insufficient reporting of stratified data in included studies but this was an additional or supplementary analysis in the protocol, not the main (overall) analysis as conducted in the study.
- We have added the complete search strategy for each database (PubMed, Cochrane CENTRAL, CINAHL, Embase, Scopus) as under the search strategy in supplementary file. We included all MeSH/Emtree terms, free-text keywords, Boolean operators, truncation symbols, and applied filters. We also included the search strings and the methods used to search google scholar, abiding by the best recommended practices in research methodology.
- We appreciate the reviewer highlighting the critical potential for confounding by past smoking history among exclusive vapers. The reported adjusted associations included prior tobacco smoking as a covariate to mitigate this specific bias brought by history of prior tobacco smoking. However, we have added a specific statement to the Limitations section acknowledging that, despite these statistical adjustments, residual confounding from past smoking history cannot be definitively ruled out: "residual confounding from prior combustible tobacco use remains a potential limitation.”
- We have reworked the outcomes section in the methods to specify that dyslipidemia was defined according to the The National Cholesterol Education Program (NCEP) Adult Treatment Panel III (ATP III) criteria. Studies reporting dyslipidemia mostly had this criteria as part of their definition for dyslipidemia, authors of included studies were also contacted to clarify the dyslipidemia definitions in their studies.
- The quality assessment section also needed a comprehensive revision to allow better readability and for a more clear and transparent approach. We have added a brief justification for selecting the MASTER scale: it provides a comprehensive, numerical assessment of methodological rigor across observational epidemiological studies, evaluating not only bias but also design, analysis, and interpretation quality, making it well-suited for this review. All seven MASTER domains are now explicitly named (Equal Recruitment, Retention, Ascertainment, Implementation, Prognosis, Sufficient Analysis, Temporal Precedence). We clarify that the same approach discussed in screening and data extraction was applied to quality assessment. Studies were divided into 3 thirds with and each third of studies was assessed independently by a pair of reviewers, with each reviewer assessing all studies then comparing with their pair. Disagreements were resolved by a senior reviewer. Quality scores were incorporated into the quality-effects model weighting but did not trigger study exclusion.
- We have now explicitly stated in the Results that all studies included in the odds ratio analysis reported adjusted estimates; no unadjusted ORs were used. We have clarified in the Methods that the analysis employed a Quality Effects model, which adjusts weights based on both the inverse variance and the quality of the evidence, thereby appropriately distinguishing the evidential weight of the findings. Regarding high heterogeneity (e.g., TG), we retained the pooled synthesis for transparency and to quantitatively assess whether a common effect exists, an insight not achievable through a purely descriptive approach. Furthermore, high heterogeneity likely comes from varying effect sizes in one direction, rather than evidence going in opposite direction. These outcomes are interpreted in text with appropriate caution to avoid overstating the results.

***Additional changes as suggested by other reviewers:***

- **Screening of studies:**
  - Title/abstract screening was performed independently by six reviewers working in three pairs (two reviewers per record) using Rayyan (Rayyan.ai, 2023). Full-text assessment was also conducted independently by the same six reviewers in three pairs that are different from original three pairs matches as an attempt to minimize reviewer bias. Discrepancies at either stage were resolved by discussion or adjudication by a senior reviewer. This section was indeed redundant and was revised well to for better readability and clarity.
  - The phrase "where applicable" regarding exclusion reasons has been removed; all exclusions after full-text screening did indeed include explicit reasons mentioned in the PRISMA diagram.
  - EndNote (Version 21, Clarivate Analytics) is now cited as the reference management software.
- **Eligibility Criteria**
  - Explicitly stated inclusion of any observational study design (cross-sectional, case–control, and cohort studies).
  - Defined "exclusive e-cigarette users" as individuals reporting current e-cigarette use regardless of past smoking history, with no concurrent combustible tobacco use at the time of assessment.
  - Specified the comparator as "non-smokers" (individuals reporting no current use of e-cigarettes or combustible tobacco), as consistently defined across included studies.
- **Outcomes**
  - We have revised the Outcomes section to state that primary outcome was dyslipidemia, and defined as meeting *any* of the following thresholds based on the National Cholesterol Education Program Adult Treatment Panel III (NCEP ATP III) : TC ≥200 mg/dL (≥5.2 mmol/L), LDL C ≥130 mg/dL (≥3.4 mmol/L), HDL C <40 mg/dL (<1.0 mmol/L) in men or <50 mg/dL (<1.3 mmol/L) in women, and TG ≥150 mg/dL (≥1.7 mmol/L). Threshold sources are now cited in text. Secondary outcomes included a comparison of continuous lipid levels (mg/dL).
- **Data Extraction**: The Data Extraction section has been comprehensively revised and reworked to state that:
  - Mean lipid levels and SDs were extracted from *all* studies whenever available, regardless of whether adjusted effect estimates were reported, to enable secondary analyses. Studies contributed to both primary (adjusted effects) and secondary (raw means) analyses when both data types were present.
  - Six reviewers worked in three pairs; within each pair, reviewers independently extracted *all* data fields (study characteristics, demographics, outcomes) using a standardized Excel form. Discrepancies were resolved by discussion or third-reviewer adjudication. This was similar to the distribution in the screening processes mentioned above.
  - The narrative now presents study characteristics before outcomes for improved logical flow.
- **Data Synthesis**: We have comprehensively revised this section and added needed details for the section to state that:
  - The quality-effects model was the *primary* analysis; the random-effects model served as a *sensitivity analysis* to assess robustness and enable comparison with conventional meta-analyses.
  - A three-tier hierarchy guided effect estimate selection: (1) adjusted estimates prioritized; (2) unadjusted estimates pooled when adjusted data unavailable; (3) raw means/SDs used only when neither adjusted nor unadjusted effects were reported.
  - Leave-one-out sensitivity analyses were performed *post-hoc* for all outcomes; a study was deemed influential if its removal reduced *I*² by ≥20 percentage points or narrowed the 95% CI width by ≥30%.
  - Publication bias was assessed using Doi plots (visual) and the LFK index (quantitative); the LFK index (>2 or <−2 indicating major asymmetry) provided the primary inference, with Doi plots as complementary visual assessment.

**5) Results**
***Comment*: 5.1 Study selection / PRISMA**: Flow is clearly reported (4,160 identified; 7 included).

- Include the full exclusion table (with reasons per full-text) in the supplement if not already provided.

***Comment*: 5.2 Study characteristics (Table 1**): The table is helpful but would benefit from additional columns for:

(a) how "exclusive vaping" was defined (self-report? timeframe?);

(b) smoking history (never/former/unknown); and

(c) lipid measurement method (fasting vs non-fasting; lab vs self-report).

These features are likely drivers of heterogeneity and bias.
**Comment: 5.3 Lipid-specific findings**

- **(TC**): High heterogeneity and publication bias signals are present; the leave-one-out sensitivity analysis suggests one study drives uncertainty. Highlight which clinical or design features of that study differ (e.g., population, fasting status, control definition).
- **( HDL & TG**): The adjusted analyses (aORs) are the most convincing pieces—worth emphasizing—but also clarify which confounders were adjusted for in each study (in text or supplement).
- **(LDL/VLDL**): Results appear more consistent but remain largely unadjusted. Maintain cautious wording and avoid clinical recommendations based solely on unadjusted differences.

**Comment: 5.4 GRADE**: Evidence certainty ranging from very low to low is appropriately justified (downgrades for heterogeneity, bias, publication bias concerns). Consider presenting a compact "Summary of Findings" table in the main text (with detailed tables in supplement) as this is often the single most useful output for readers.

***Response****:* We thank the reviewer for this detailed assessment of our findings. We have revised the Results and Discussion to address these points:

- The reasons for exclusion of full text screening are summarized in the PRISMA diagram under the “reports excluded” section.
- We appreciate the reviewer's suggestion to include these critical study features. We agree that these details are important for interpreting heterogeneity. However, adding these columns directly to Table 1 would compromise readability due to space constraints. Instead, we have moved the detailed lipid measurement methods to **Supplementary Studies methods section**. The definition of "exclusive vaping" was applied strictly according to the criteria outlined in the Methods section for all included studies. Regarding smoking history, we noted that reporting was very limited across the primary studies.
- We have now explicitly justified the sensitivity analysis for Hasan 2024: "Leave-one-out sensitivity analyses showed that heterogeneity substantially decreased (I² reduced from 86.9% to 0%) and the confidence interval narrowed upon exclusion of Hasan 2024, the only case-control study, conducted exclusively in Iraqi men with an imbalanced design (100 vapers vs. 50 controls), indicating disproportionate influence on the pooled estimate."
- To improve transparency regarding the adjusted analyses for HDL and TG, we have added the specific confounders adjusted for in each study directly into the **Figure Legends**. We have carefully revised the wording for LDL and VLDL findings to ensure clinical recommendations are cautious and reflect the limitations of unadjusted data.
- Added Table 2 summarizing pooled estimates, participants, heterogeneity, models, and GRADE certainty for each lipid outcome.

***Additional changes as suggested by other reviewers:***

- Specified and cited the six studies were cross-sectional (Lim 2024, Matheson 2024, Okafor 2022, Majid 2021, Kim 2020, Badea 2019) and one was case–control (Hasan 2024).
- Replaced all instances of "strength of recommendation" with "certainty of evidence" or “quality of evidence” throughout the manuscript.
- Revised Section 3.9 to transparently report GRADE ratings per outcome with explicit reasons for downgrading (e.g., TC and LDL downgraded to very low due to suspected publication bias; VLDL rated very low due to only two studies).
- Updated Figures 3 and 6 to label outcomes as "HDL dyslipidemia" and "TG dyslipidemia," with NCEP ATP III threshold definitions provided in figure footnotes.
- Additional changes made to section 3.4 “Association between vaping and levels of TC”:
  - Clarified statistical interpretation and added clinical context directly within the Results section:
    - "The overall weighted mean difference (WMD) was 3.66 mg/dL higher in vapers compared to non-vapers (95% CI −13.89 to 21.20). However, the confidence interval crossed the null value (zero), indicating no statistically significant difference. Even if statistically significant, a mean difference of 3.66 mg/dL would likely be of minimal clinical relevance for cardiovascular risk assessment."
  - Added methodological caveats regarding heterogeneity and bias metrics with sparse data:
    - "However, with only five studies included, both the magnitude of heterogeneity and the bias assessment should be interpreted cautiously, and potential sources of heterogeneity could not be explored due to insufficient data for meaningful subgroup or meta-regression analyses."

**6) Discussion**
***Comment***: Strengths section is fair and transparent, including plausible biological mechanisms (nicotine → catecholamines → lipolysis → FFAs → TG/VLDL).
***Comment***: Tighten causal language throughout: cross-sectional designs and residual confounding warrant "association" rather than "effect" terminology.
***Comment***: Expand discussion of smoking-history confounding: former smoking is likely the biggest alternative explanation for observed associations and deserves prominence in interpretation (not only in limitations).
***Comment***: Consider discussing reverse causality/health selection bias: individuals with dyslipidemia or health concerns may switch from cigarettes to vaping (or vice versa), which can bias observed associations.
***Comment***: When comparing to cigarette or waterpipe studies, explicitly acknowledge that while mechanisms may overlap (nicotine/inflammation), exposure profiles differ substantially (device types, aerosol constituents, puffing topography). This will make the discussion more nuanced and less dependent on cigarette analogies.

***Response****:* We are grateful for these insightful comments, which have significantly improved the nuance and scientific rigor of our Discussion. We have revised the text to strictly adhere to observational language, distinguish between mechanism and exposure, and explicitly address the "former smoker" and "health selection" phenomena as major interpretative factors. The following changes have been made:

- **Causal Language**: We have systematically replaced causal terms (e.g., "effect," "impact," "leads to") with observational terminology throughout the Discussion and Conclusion.
  - *Revised Text:* "This systematic review and meta-analysis **identified** varying associations..." and "Adjusted analyses... indicated a stronger **independent** association..."
  - *Revised Text:* "Though marketed as safer alternatives, **observational evidence suggests** e-cigarettes may negatively impact lipid metabolism..."
- **Exposure Profiles vs. Mechanisms:** We added a specific distinction between the shared mechanism (nicotine) and the differing exposure profiles (combustion products) to avoid over-reliance on the cigarette analogy.
  - *Inserted Text:* "While e-cigarettes share the delivery of nicotine with these traditional products, it is important to note that the exposure profiles differ significantly; specifically, e-cigarettes lack the combustion-generated carbon monoxide and tar found in conventional cigarettes ..."
- **Smoking History & Residual Confounding:** We expanded the interpretation of the HDL and LDL findings to explicitly cite the lingering metabolic effects of past smoking as a primary alternative explanation, rather than relegating this solely to the limitations section.
  - *Inserted Text:* "However, in interpreting these findings for e-cigarettes, the potential for residual confounding by past smoking history is substantial. Many e-cigarette users are former combustible cigarette smokers, and the observed lower HDL may partially reflect the lingering metabolic effects of past smoking rather than vaping alone ..."
- **Reverse Causality / Health Selection:** We incorporated the concept of "sick switchers" to address the potential for reverse causality.
  - *Inserted Text:* "Furthermore, 'health selection' bias cannot be ruled out, wherein individuals with pre-existing health concerns or dyslipidemia switch from cigarettes to vaping to mitigate harm, thereby clustering higher-risk lipid profiles within the vaping group ..."

***Additional changes as suggested by other reviewers:***

We have revised the opening paragraph of the Discussion to accurately reflects statistical significance, adjustment status, and GRADE ratings. It now reads “This systematic review and meta-analysis revealed varying associations between e-cigarette use and serum lipid profiles. Overall, unadjusted analysis suggested higher mean levels of TC, LDL, VLDL, and triglycerides and lower HDL in vapers compared with non-vapers; total cholesterol showed a non-significant mean difference and the certainty of evidence was very low. Adjusted analyses, available only for HDL and triglycerides, indicated a stronger association with dyslipidemia patterns - specifically lower HDL and higher triglycerides - and the certainty of evidence was low.”.

**7) Conclusion**
***Comment***: The conclusion aligns with findings but should make the "low certainty" message even more prominent, as this is what readers and journalists will most likely quote.
***Comment***: Consider adding a final sentence specifying what "robust longitudinal studies" should measure: device type, nicotine concentration, duration/intensity of use, fasting lipid panels, validated smoking history (never/former/never smoker), and ideally repeated measures to assess temporal relationships.

***Response****:* The Conclusion has been revised to provide a transparent reflection of the study’s findings and their limitations without providing a false overstating of the conclusions. It now reads “This synthesis suggests possible differences in lipid levels among vapers compared with non-vapers. However, these findings are based on findings from a combination of adjusted and unadjusted analyses from observational studies. Several associations had substantial heterogeneity, and GRADE certainty ranged from very low to low. Consequently, the evidence does not support firm conclusions about an association - or its direction - between vaping and lipid profiles. Further robust longitudinal studies with adjustment for key confounders are needed to clarify these associations with greater certainty.”

We are grateful for the opportunity to improve our manuscript through this rigorous review. All changes have been implemented carefully, and we believe the revised version is substantially strengthened in transparency, accuracy, and interpretive balance of our manuscript. Thank you again for your valuable contribution to our work.
