# Supplementary material for: The association between e-cigarette use and dyslipidemia: A systematic review and meta-analysis of observational studies
Source: PLoS One. 2026 Oct 26;21(10):e0361200. doi: 10.1371/journal.pone.0361200 (PMC13704818; doi:10.1371/journal.pone.0361200)
Supplement: Supplementary file 6 [file pone.0361200.s007.docx]

# Response to Reviewer Comments

Manuscript title: The Association Between E-cigarette Use and Dyslipidemia: A Systematic Review and Meta-analysis of Observational Studies

Dear Editors and Reviewers,

We sincerely thank you for your continued engagement with our manuscript and for the detailed, constructive feedback provided in this second round of review. We are grateful that you found the revised manuscript substantially improved in clarity, balance, and readability. Your remaining comments helped us identify areas where our earlier responses were incomplete or inconsistent, and we have now addressed each point with targeted revisions. Below, we provide a point-by-point response.

# Reviewer Comment 1

*Thank you for the revised manuscript and accompanying response letter. The manuscript is certainly better than the original version. I am thankful for the revisions to temper the language, clarify the observational design, better define exclusive vaping and the comparison group, separate primary and secondary outcomes, and clarify the results and conclusion. Overall, these changes help the manuscript, and it is now better, fairer, and easier to read.*

**Response:**

We appreciate your generous feedback. We are pleased that the revisions improved the manuscript’s clarity, balance, and readability. Your thoughtful comments throughout the review process have strengthened the work substantially, and we are grateful for the time and expertise you have invested.

# Reviewer Comment 2

*However, I do not think it is entirely responsive yet. I have a few concerns that were not fully addressed, or not addressed in a fully consistent way in the response letter and the manuscript. First, the distinction between adjusted binary lipid outcomes and unadjusted continuous lipid outcomes is now much clearer in the manuscript, and I appreciate that the abstract, discussion, and conclusion now clearly state this as an issue. However, this is still the major interpretability issue with the paper, and I think it should be kept front and center throughout the manuscript. I appreciate the improvements to the discussion, where you now clearly state that only adjusted data were available for HDL and triglycerides, while the rest of the lipid analyses were mostly unadjusted. That is a major improvement. However, I would encourage one more pass to ensure every sentence in the results and discussion is equally clear about this issue, particularly when discussing the LDL and VLDL outcomes.*

**Response:**

Thank you for this important comment. We agree that the distinction between adjusted binary outcomes and unadjusted continuous lipid outcomes is central to interpreting this meta-analysis. We therefore made another pass through the Abstract, Results, Discussion, and Conclusion to ensure that this distinction remains explicit throughout the manuscript.

Specifically, we revised the Abstract to distinguish adjusted odds ratios for dyslipidemia from unadjusted weighted mean differences for continuous lipid levels. We also revised each lipid-specific Results section to state whether adjusted or unadjusted estimates were available:

- Section 3.4, TC: none of the studies reported adjusted effect sizes for the association between vaping and total cholesterol; the analysis was based on unadjusted mean differences.
- Section 3.5, HDL: adjusted odds ratios were available from two studies, while continuous HDL analyses were based on unadjusted mean HDL levels.
- Section 3.6, LDL: no adjusted effect sizes were available; the LDL meta-analysis was based on unadjusted mean differences.
- Section 3.7, VLDL: neither study reported adjusted effect sizes; the VLDL meta-analysis was based on two studies reporting unadjusted means.
- Section 3.8, TG: adjusted odds ratios were available from two studies, while the continuous TG analysis was based on unadjusted mean TG levels.

We also revised the Discussion to keep this issue central to interpretation. The LDL paragraph now states, in part:

*“For LDL, our analysis was based entirely on unadjusted mean differences and showed higher LDL levels among e-cigarette users compared to non-users, with a WMD of 12.40 mg/dL and moderate heterogeneity. Our results correspond with those reported in observational studies exploring cigarette smoking and LDL. For instance, an observational study showed a measurable difference in lipid parameters between cigarette smokers and non-smokers, indicating markedly higher levels of LDL cholesterol in smokers compared to non-smokers (53). The observed LDL increases are likely linked to nicotine-induced lipolysis and FFA release (19, 43). Because these estimates were not adjusted for smoking history or other cardiovascular risk factors, they should be interpreted cautiously.”*

The VLDL paragraph now states, in part:

*“In relation to VLDL, two studies reporting unadjusted means showed higher VLDL levels among e-cigarette users than non-users, with a pooled uWMD of +3.23mg/dL. Our findings align with limited but growing evidence that suggests vaping may influence VLDL levels. One meta-analysis reported that young smokers aged between 8-19 showed substantially higher serum VLDL cholesterol levels compared to non-smokers. This increase was also associated with increased LDL cholesterol and triglycerides, along with reduced HDL cholesterol, suggesting that cigarette smoking may lead to early lipid profile disturbances (53). Because only two studies contributed to this analysis and neither provided adjusted estimates, the certainty of evidence remains very low.”*

# Reviewer Comment 3

*Second, I do not think the data extraction section fully addresses the original comment. You now clarify that 6 reviewers working in 3 pairs independently extracted all data fields using a standard Excel form. However, the original comment also asked if the Excel form was pilot-tested and if inter-extractor agreement was assessed before proceeding with full extraction. That part of the original comment appears to have gone unanswered. Please either fill this in in the manuscript, if this was done, or clearly state that this was not formally assessed. As it stands, I feel the response is incomplete.*

**Response:**

We appreciate the reviewer’s request for greater methodological transparency. We revised the Data Extraction section, Section 2.6, to explicitly address both pilot-testing and inter-extractor agreement.

The Excel extraction form was not formally pilot-tested on a subset of studies. Because only seven studies met the inclusion criteria, we developed a standardized form to capture all prespecified study, participant, exposure, comparator, and lipid-outcome variables and applied it uniformly to all included studies.

We also clarified that formal inter-rater reliability or inter-extractor agreement statistics were not calculated before full extraction. Six authors worked in three independent pairs, and within each pair, reviewers extracted all data fields independently. Discrepancies were resolved through discussion or adjudication by a third reviewer.

The revised manuscript text now states:

*“Data were extracted using a standardized Microsoft Excel spreadsheet. The extraction form was not formally pilot-tested on a subset of studies because only seven studies met the inclusion criteria; instead, the form was developed to capture all prespecified study, participant, exposure, comparator, and lipid-outcome variables and was applied uniformly to all included studies. Six authors (HR, SM, AH, HA, WA, JN) worked in three pairs, and within each pair, reviewers extracted all data fields independently. Formal inter-rater reliability or inter-extractor agreement statistics were not calculated before full extraction. Discrepancies were resolved through discussion or adjudication by a third reviewer. Extracted data included study characteristics, participant demographics, exposure definitions, comparator definitions, covariates, and lipid outcome data.”*

We hope this fully addresses the concern.

# Reviewer Comment 4

*Third, there appears to be some inconsistency regarding the publication bias analysis that should be rectified. In the response letter, you state that the Doi plots provided the primary inference and the LFK index was supplementary. However, in the text itself, the LFK index is stated to provide the primary inference and the Doi plots were used as a supplementary visual analysis. The two statements cannot both be true. Please pick one and apply it consistently in the manuscript and the response letter, and clarify how discrepancies between the visual and quantitative analyses were managed.*

**Response:**

Thank you for identifying this inconsistency. We apologize for the confusion in our previous response letter. Our intended approach was to use the LFK index as the primary quantitative criterion for assessing asymmetry, with Doi plots used as complementary visual tools.

We have now aligned the response letter and manuscript accordingly. The Abstract Methods section now states that publication bias was assessed using Doi plots and the LFK index, with the LFK index used as the primary quantitative criterion. The Methods section further clarifies how discrepancies between the visual and quantitative assessments were managed.

The revised Methods text states:

*“Publication bias was assessed using complementary visual and quantitative methods: (1) visual inspection of Doi plots, and (2) quantitative assessment via the LFK index (asymmetry >2 or <-2 interpreted as major bias) (32). The LFK index served as the primary basis for inference regarding bias presence; when visual and quantitative assessments diverged, the quantitative LFK result was prioritized to minimize subjectivity. Findings from both tools are reported to enhance transparency.”*

# Reviewer Comment 5

*Fourth, some of the responses to big picture conceptual points are too vague. For example, one of the major comments highlighted the issues of confounding, the definition of exclusive vaping, and the problem of interpreting the mixture of adjusted and unadjusted outcomes. The response to that broad concern was essentially that the language is softer and the flow is better. While the flow was indeed improved in some of the later sections, the response would be better if it pointed to the exact changes made in the methods, results, and discussion. This would make the response letter more convincing and easier for editors to evaluate.*

**Response:**

We agree and have revised this response to map each conceptual concern to specific manuscript changes.

First, regarding confounding by combustible tobacco smoking, we revised the Introduction to clarify that the review focused on exclusive vaping to reduce confounding from concurrent combustible tobacco use, while acknowledging that past combustible tobacco use may still confound observed lipid associations. The revised Introduction states:

*“To reduce confounding from concurrent combustible tobacco use, this review focused on “exclusive vaping,” defined here as e-cigarette use without concurrent use of combustible tobacco products. However, past combustible tobacco use may still confound observed lipid associations and was therefore considered in the interpretation of the findings.”*

Second, regarding the comparator group, we revised Section 2.4 to avoid overgeneralizing the comparator definition. The revised Eligibility Criteria state:

*“Studies were eligible if they compared exclusive e-cigarette users, defined as current e-cigarette users without concurrent tobacco smoking, regardless of their past smoking history, with non-current e-cigarette users or non-smokers.”*

Third, regarding adjusted and unadjusted outcomes, we revised the Abstract, Methods, Results, Discussion, and Conclusion to distinguish adjusted odds ratios for binary dyslipidemia outcomes from unadjusted weighted mean differences for continuous lipid outcomes. In the Methods, we describe the hierarchy of effect estimates, prioritizing adjusted estimates when available and clearly stating when unadjusted estimates or raw means were used. The revised Data Synthesis section states:

*“For each lipid parameter (TC, HDL, LDL, VLDL, and TG), we conducted meta-analyses using a hierarchical approach to effect estimates: adjusted effect measures (aOR, and their 95% CIs) were prioritized when available; if adjusted estimates were absent, unadjusted effect measures were extracted and pooled; when neither adjusted nor unadjusted effect estimates were reported, raw continuous data (mean lipid levels and standard deviations) were used to compute weighted mean differences (WMDs). This hierarchy ensured maximal inclusion of eligible effect sizes while prioritizing estimates least susceptible to confounding.”*

Fourth, we expanded the Discussion and Limitations to emphasize residual confounding and possible health-selection bias. The Limitations section now states:

*“A major limitation is residual confounding by combustible tobacco history. Although the review focused on exclusive current e-cigarette use and excluded dual use where possible, many exclusive e-cigarette users may be former smokers. Lipid abnormalities caused by previous combustible tobacco exposure may persist after switching to e-cigarettes, making it difficult to isolate the metabolic effect of vaping itself. Reverse causality is also possible, because individuals with cardiovascular risk factors may switch from cigarettes to e-cigarettes as a perceived harm-reduction strategy.”*

# Reviewer Comment 6

*Fifth, I appreciate the conclusion is better aligned with the low certainty evidence, and I appreciate you now avoid the stronger causal language. However, I think one of the earlier suggestions is only partially fulfilled. You were asked to clarify what future robust longitudinal studies should actually measure, such as device type, nicotine concentration, duration and intensity of use, fasting lipid level assessment, validated smoking status, and repeated measurements over time. You now mention adjustment for key confounders in the conclusion, but do not provide the specific list of suggestions as requested. I think you could strengthen the conclusion by incorporating one additional sentence with concrete suggestions for future study designs.*

**Response:**

Thank you for noting this. We agree that the conclusion should provide more concrete guidance for future studies. We revised both the Abstract Conclusion and the main manuscript Conclusion to specify the key design elements that future longitudinal studies should include.

The revised Abstract Conclusion now calls for well-designed longitudinal studies using validated smoking-status assessment, detailed vaping exposure measures, fasting lipid panels, repeated lipid measurements, and adjustment for key cardiometabolic confounders.

The revised main Conclusion now has this added statement:

*“Further well-designed longitudinal studies should prospectively collect detailed vaping exposure data, including device type, nicotine concentration, and duration and intensity of use; use validated assessments of smoking status to distinguish never smokers, former smokers, current smokers, and dual users; obtain fasting lipid panels; and adjust for key cardiometabolic and behavioral confounders to better assess temporality and clarify these associations.”*

# Reviewer Comment 7

*Overall, I think the paper has improved a great deal, and many of the major concerns were addressed meaningfully. My suggestion is for a targeted revision, not a full redo. The bulk of the remaining work is not to reframe the paper, but to tighten up a few incomplete responses, resolve some internal inconsistencies, and ensure the response letter and manuscript match perfectly. Once that is done, the paper will be in a much stronger place.*

**Response:**

We thank the reviewer for this constructive summary and agree that the remaining issues required targeted correction rather than a full reframing of the manuscript. We have carefully reviewed the manuscript and response letter together, corrected the remaining internal inconsistencies, clarified the methodological points identified above, and aligned the response letter with the revised manuscript. We believe these targeted revisions have strengthened the manuscript and improved the transparency and interpretability of the findings.

Sincerely,

Tawanda Chivese
