# Supplementary material for: The association between e-cigarette use and dyslipidemia: A systematic review and meta-analysis of observational studies
Source: PLoS One. 2026 Oct 26;21(10):e0361200. doi: 10.1371/journal.pone.0361200 (PMC13704818; doi:10.1371/journal.pone.0361200)
Supplement: Supplementary file 7 [file pone.0361200.s008.docx]

Response to reviewers - [PONE-D-25-42648R2]

Sept 7, 2026

Dear Editor, many thanks to the reviewers for their comments. Please find before the response to the reviewers’ comments

1. The authors have substantially addressed the previous comments; however, several minor revisions remain necessary before the manuscript can be accepted. Please ensure that the response letter accurately reflects the changes made in the manuscript, particularly regarding the Abstract and Introduction.

Response – we have done this

2. The Abstract should explicitly identify the LFK index as the primary quantitative criterion for assessing publication bias, consistent with the Methods, and the corresponding LFK values should be reported consistently for all outcomes where available.

Response – this has now been implemented

3. The Introduction should clearly acknowledge that previous combustible tobacco exposure may confound the observed associations, while the comparator definition should be harmonized across the manuscript and response letter.

Response – we have implemented this

4. In the Discussion, please revise the statement that “the majority” of the LDL findings were unadjusted, as all LDL estimates were unadjusted, and clarify that neither VLDL study provided adjusted estimates and that the certainty of evidence was very low.

Response – we have implemented this suggestion.

5. Please also consolidate the repetitive recommendations in the Conclusion and correct the remaining duplicated, incomplete, or grammatically incorrect sentences, spacing problems, and formatting inconsistencies throughout the manuscript.

Response – we have implemented this now.
