# Supplementary material for: The association between e-cigarette use and dyslipidemia: A systematic review and meta-analysis of observational studies
Source: PLoS One. 2026 Oct 26;21(10):e0361200. doi: 10.1371/journal.pone.0361200 (PMC13704818; doi:10.1371/journal.pone.0361200)
Supplement: Peer review history

# Peer review: Decision Letter 0

**DOI:** 10.1371/journal.pone.0361200.r001

**Authors:** Zypher Jude Regencia

21 Jan 2026

Dear Dr.  Chivese,

Thank you for submitting your manuscript to PLOS ONE. After careful consideration, we feel that it has merit but does not fully meet PLOS ONE’s publication criteria as it currently stands. Therefore, we invite you to submit a revised version of the manuscript that addresses the points raised during the review process.

Please submit your revised manuscript by Mar 07 2026 11:59PM. If you will need more time than this to complete your revisions, please reply to this message or contact the journal office at plosone@plos.org. When you're ready to submit your revision, log on to https://www.editorialmanager.com/pone/ and select the 'Submissions Needing Revision' folder to locate your manuscript file.

- A letter that responds to each point raised by the academic editor and reviewer(s). You should upload this letter as a separate file labeled 'Response to Reviewers'.
- A marked-up copy of your manuscript that highlights changes made to the original version. You should upload this as a separate file labeled 'Revised Manuscript with Track Changes'.
- An unmarked version of your revised paper without tracked changes. You should upload this as a separate file labeled 'Manuscript'.

If you would like to make changes to your financial disclosure, please include your updated statement in your cover letter. Guidelines for resubmitting your figure files are available below the reviewer comments at the end of this letter.

We look forward to receiving your revised manuscript.

Kind regards,

Zypher Jude G. Regencia, Ph.D.

Academic Editor

PLOS One

Journal Requirements:

When submitting your revision, we need you to address these additional requirements.

1.Please ensure that your manuscript meets PLOS ONE's style requirements, including those for file naming. The PLOS ONE style templates can be found at

https://journals.plos.org/plosone/s/file?id=wjVg/PLOSOne_formatting_sample_main_body.pdf and

https://journals.plos.org/plosone/s/file?id=ba62/PLOSOne_formatting_sample_title_authors_affiliations.pdf

2. Please include captions for your Supporting Information files at the end of your manuscript, and update any in-text citations to match accordingly. Please see our Supporting Information guidelines for more information: http://journals.plos.org/plosone/s/supporting-information.

3. If the reviewer comments include a recommendation to cite specific previously published works, please review and evaluate these publications to determine whether they are relevant and should be cited. There is no requirement to cite these works unless the editor has indicated otherwise.

Additional Editor Comments:

Because there was difficulty in securing a second reviewer, I acted as the second reviewer, as well as the Academic Editor of this paper.

Reviewer's Responses to Questions

**Comments to the Author**

1. Is the manuscript technically sound, and do the data support the conclusions?

Reviewer #1: Yes

Reviewer #2: Partly

**********

2. Has the statistical analysis been performed appropriately and rigorously?

Reviewer #1: Yes

Reviewer #2: No

**********

3. Have the authors made all data underlying the findings in their manuscript fully available?

The PLOS Data policy

Reviewer #1: Yes

Reviewer #2: No

**********

4. Is the manuscript presented in an intelligible fashion and written in standard English?

Reviewer #1: Yes

Reviewer #2: Yes

**********

Reviewer #1: Abstract

The abstract would benefit from greater clarity and completeness. Please report the total number of participants included across all studies, in addition to the number of studies. In addition, the reported p-value is potentially confusing, as it appears to refer to the heterogeneity test rather than the effect estimate. I suggest either explicitly labeling it as the p-value for heterogeneity or removing it from the abstract altogether, as heterogeneity p-values are uncommon in abstracts and may be misinterpreted by readers.

Data Sources and Search Terms

• Please write the search full string including Boolean operators, MeSH terms, truncation, and filters. Were they applied in all the databases. I recommend to show how the actual search done per database in the supplementary.

Screening of studies

• Screening of studies section appears to be redundant and confusing. Please clarify if a preliminary screening was done prior to full text screening. It is unclear and confusing because of the statement “Preliminarily included study records were retrieved in full text” and was followed by “Full texts of potentially eligible studies were then retrieved, and six reviewers, working in random three random pairs, independently assessed them.” Please clarify how many reviewers per stage of screening were involved (title and abstract screening / full text screening).

• I believe that all studies excluded after full screening should have a reason for exclusion and I would suggest removing the term if applicable.

• What is a random three random pairs mean? Please clarify.

• Please detail the software used as well as include in the citation. For example, EndNote (Version 21, Clarivate Analytics).

Eligibility Criteria:

• Please include the type of observational studies i.e. longitudinal (cohort), case-control, cross-sectional studies) were considered as this would affect the results on association.

• Please define what exclusive e-cigarette use. It may sound contradictory with the statement “who currently use e-cigarettes, regardless of their past vaping or smoking history.”

• Please clarify that self-reported dyslipidemia was excluded. Suggest to add “Only” in the statement “ Biochemically validated measures of dyslipidemia were accepted.” If that is the purpose of the statement.

• I noticed that the eligibility and outcomes sections do not specify the comparator group. Since the review aims to assess the association between exclusive e-cigarette use and lipid levels/dyslipidaemia, it is important to clarify what population the e-cigarette users were compared against (e.g., never-smokers, non-e-cigarette users, combustible cigarette smokers, or mixed controls). Without a clearly defined comparator, the direction and interpretation of the association may be ambiguous. Please specify the comparator criteria in the methods, and ensure consistency across eligibility criteria, data extraction, and statistical analyses. This clarification will strengthen the internal validity and reproducibility of the review.

Outcomes:

• Please explicitly state whether dyslipidaemia was defined by meeting any single lipid threshold or a combination of thresholds. Please mention source of your definition of dyslipidemia. In addition, present the primary (binary dyslipidaemia) and secondary (continuous lipid levels) outcomes more distinctly to avoid conceptual overlap. Ensure units and symbols are consistent (e.g., ≤, ≥) and specify whether thresholds were based on guideline sources. Overall, clearer wording will greatly improve interpretability.

Data Extraction:

• I have a concern about the data extraction approach. From the description “Mean levels of lipids and their standards deviations were extracted from studies which did not report adjusted effect sizes”, it appears that mean lipid levels and standard deviations were only extracted from studies that did not provide adjusted effect estimates. If this interpretation is correct, it implies that studies reporting effect measures (e.g., adjusted mean differences, ORs, β-coefficients) may not have contributed raw lipid levels to the secondary outcome analyses. This raises two issues that need clarification:

o Were mean lipid levels and SDs extracted from studies that reported adjusted effect sizes whenever those data were available (either in the main text, tables, or supplementary files)? If not, this could lead to unnecessary exclusion of eligible data.

o Would excluding such data influence the validity of the secondary meta-analysis? Selectively extracting continuous data only from a subset of studies may bias the pooled mean estimates or reduce statistical power.

• I recommend clarifying the extraction rule and, if applicable, justifying why studies that report effect measures could not contribute to the continuous outcome analysis, even when raw values were present. A sentence explaining how overlap between primary and secondary outcomes was handled would strengthen transparency.

• It would be useful to specify whether a standardized and piloted data extraction form was used in Excel, and whether inter-extractor agreement was assessed before full extraction began.

• I find the extraction section confusing and should be clarified. As written, “six authors independently extracted data in pairs” is contradictory, because extraction cannot be both independent and paired at the same time. Please specify whether each reviewer extracted data independently and was then paired for comparison, or whether each pair extracted jointly. It may be clearer to state: “data were extracted independently by pairs of reviewers, with discrepancies resolved through discussion or by a third reviewer.”

• Please clarify whether extraction was done independently and in duplicate for all data fields (study characteristics, outcomes, demographics) or only for effect estimates.

• I suggest to start of the with the study characteristics and followed by the outcomes.

Quality Assessment and Certainty

• It would help to briefly explain why the MASTER scale was selected rather than more commonly used tools for observational studies (e.g., Newcastle–Ottawa Scale or ROBINS-I). A short justification will assist readers who may be unfamiliar with MASTER.

• The sentence structure suggests that seven domains were assessed, but the manuscript lists only six types of bias. Please align terminology and ensure all domains are clearly named.

• Please clarify whether assessment was performed independently by multiple reviewers, how disagreements were resolved, and whether results were incorporated into the interpretation (e.g., sensitivity analysis, subgrouping by quality, or exclusion based on threshold scores).

Data Synthesis

• It is unclear why both the quality-effects and random-effects models were applied and which is considered the primary analysis—please clarify the rationale and interpretive hierarchy.

• It is not entirely clear whether studies that reported unadjusted odds ratios were excluded from the pooled OR analysis. The methods imply that only adjusted ORs were extracted and synthesized; if so, this could lead to the exclusion of eligible effect sizes and potential bias. Please clarify whether unadjusted ORs were pooled when adjusted estimates were unavailable, and explain the rationale either way.

• Please clarify the rationale for using several publication bias tools, specify which method provides the primary inference, and explain how discrepancies between methods were handled.

Results

To improve clarity and readability, I suggest adding a summary table in the main text that presents, for each lipid outcome, the pooled weighted mean difference (WMD) (with 95% CI), the pooled adjusted odds ratio where available, number of studies, total participants, heterogeneity (I²), model used (Quality adjusted/Random effects) and GRADE certainty. The detailed forest plots could then be moved to the Supplementary Materials.

“The seven included studies were published between 2019 and 2024, six were cross-sectional studies and one was a case-control study.” Please specify which studies were cross-sectional and which was case-control by citing the relevant references immediately after each study design.

“After meta-analysis, the overall weighted mean difference (WMD) showed a higher mean TC level in vapers, compared to non-vapers, of 3.66 mg/dL (95%CI -13.89 to 21.20) with a confidence interval still suggesting uncertainty.” - The interpretation of this result needs refinement. Although the point estimate suggests a higher mean TC level among vapers, the 95% CI crosses the null and is wide, indicating no statistically significant difference and substantial imprecision. The statement “with a confidence interval still suggesting uncertainty” is vague; please explicitly state that the result is not statistically significant and interpret it cautiously. Additionally, consider commenting on the clinical relevance of a 3.66 mg/dL difference, even if statistically significant, and ensure consistency between statistical inference and narrative conclusions.

“Given the small number of included studies (n = 5), estimates of heterogeneity (I²) and assessments of publication bias using Doi plots and the LFK index are likely unstable, and conclusions regarding unexplained heterogeneity or publication bias should therefore be interpreted with caution and more tentative language.

“Sensitivity analyses showed that the uncertainty significantly declined in the overall estimates, in leave-one out analyses upon leaving out Hasan, 2024 “ – The description of the sensitivity analysis requires clarification. Please specify the rationale for excluding Hasan (2024) in the leave-one-out analysis—was this study an outlier, at high risk of bias, methodologically different, or disproportionately influential? In addition, clarify the purpose of the sensitivity analysis (e.g., to assess robustness of pooled estimates, influence of individual studies, or impact of study quality). Without this justification, it is difficult to interpret the meaning of the reported reduction in uncertainty.

The term “strength of recommendation” is not appropriate in this context. GRADE is used here to assess the certainty (quality) of evidence, not the strength of a recommendation, which requires consideration of values, preferences, balance of benefits and harms, and resource use. Please revise the wording throughout to “certainty of evidence” (or “quality of evidence”) to accurately reflect the GRADE framework.

The statement on GRADE assessment needs clarification. Please specify the reasons for downgrading the certainty of evidence to very low (e.g., risk of bias, inconsistency, imprecision, indirectness, or publication bias), rather than only reporting the overall rating. Briefly summarizing the key domains driving the downgrading in the main text would improve transparency and align with GRADE reporting standards.

Figure 3 requires clearer outcome definition and terminology. The forest plot presents odds ratios, but it is unclear whether the outcome is low HDL, HDL dyslipidaemia (defined by a specific threshold), or another binary construct. Terms such as “high HDL” or “hyper-HDL” are not standard and should be avoided. Please specify the exact HDL cut-off used (e.g., HDL <1.0 mmol/L for men, <1.3 mmol/L for women), indicate the direction of effect (OR >1 reflects higher odds of low HDL), and revise the figure title and legend accordingly to ensure correct interpretation.

The handling of outlier studies and sensitivity analyses requires clarification. In the earlier analysis, Hasan (2024) was excluded in a leave-one-out sensitivity analysis, presumably because it was influential or an outlier; however, in the HDL analysis, Matheson appears to be an outlier but was not subjected to similar sensitivity testing. Please clarify the criteria used to identify influential or outlier studies and whether these criteria were applied consistently across outcomes. In addition, please state whether sensitivity analyses were pre-specified (a priori) in the protocol or conducted post-hoc in response to observed heterogeneity. Clear justification is necessary to avoid concerns about selective analysis.

“Since this study included observational studies, the GRADE certainty level starts as low and the GRADE was unchanged (low certainty) for the outcomes of TC, LDL and VLDL, but was upgraded to moderate in HDL and TG due to adjustment for estimated confounders. GRADE was then downgraded to low and very low due to high heterogeneity across the studies, possible risk of bias (low-quality cross-sectional studies), and uncertainty about publication bias.” - The description of the GRADE assessment is confusing and appears internally inconsistent. Earlier sections report very low certainty for HDL, yet this paragraph states that HDL (and TG) were upgraded to moderate certainty due to adjustment for confounders, before being downgraded again. Please reconcile these statements to ensure consistency across the manuscript. In addition, the rationale for upgrading is not sufficiently justified: while adjustment for confounders may support upgrading for residual confounding that would bias results toward the null, this is not appropriate when the underlying studies are at high risk of bias, particularly low-quality cross-sectional designs. GRADE upgrading criteria should be explicitly stated and applied cautiously; upgrading evidence that is already seriously limited by risk of bias is generally not recommended. I suggest clearly listing the specific upgrading and downgrading decisions for each outcome and revising the narrative to align with GRADE guidance.

Discussion

“This systematic review and meta-analysis revealed varying associations between e-cigarette use and serum lipid profiles. Overall, vaping was linked to higher total cholesterol (TC), LDL, VLDL, and triglyceride (TG) levels, and lower HDL levels, although the certainty of currently available evidence is very low. Adjusted odds ratios were only available for two lipids, and from only a few studies. For the rests of the lipids, only unadjusted mean differences were available for analysis.”- This paragraph appear overstated relative to the strength and consistency of the evidence. While the text states that vaping was “linked” to higher TC, LDL, VLDL, and TG and lower HDL levels, several of these associations—particularly TC—were not statistically significant, were based on very few studies (often n≈3–5), and relied largely on unadjusted mean differences. Only TG showed a statistically significant association based on adjusted effect estimates, whereas conclusions for other lipids are driven primarily by unadjusted analyses that are vulnerable to confounding. I recommend revising the language to clearly distinguish between adjusted and unadjusted findings, avoid causal or definitive phrasing (“linked”), and explicitly acknowledge that the observed mean differences are small, imprecise, and of uncertain clinical significance. These limitations should also be reflected consistently in the GRADE assessments and overall conclusions.

Conclusion

The conclusion should be revised to more accurately reflect the substantial limitations of the evidence and avoid overstating the findings. Phrases such as “vaping is likely associated with deranged lipid levels” imply a degree of certainty that is not supported by the data, given that most analyses were based on unadjusted observational studies, several associations were not statistically significant, heterogeneity was high, and overall GRADE certainty was low or very low. I recommend using more cautious, descriptive language (e.g., “limited and low-certainty evidence suggests possible differences in lipid levels, particularly triglycerides”) and explicitly noting the uncertainty and lack of causal inference. Aligning the conclusion more closely with the stated limitations will improve internal consistency and methodological rigor.

Reviewer #2: Please see attached file.

Overall recommendation

Major revision. The topic is important and the review is promising, but the manuscript needs clearer

handling and communication of confounding (especially smoking history), clearer

separation of adjusted vs unadjusted evidence streams, and more cautious

interpretation where heterogeneity is extreme. With these improvements, it could

become a solid and useful synthesis.

**********

what does this mean?). If published, this will include your full peer review and any attached files.

If you choose “no”, your identity will remain anonymous but your review may still be made public.

**Do you want your identity to be public for this peer review?**  For information about this choice, including consent withdrawal, please see our Privacy Policy

Reviewer #1: **Yes:**  Ian Theodore Cabaluna

Reviewer #2: **Yes:**  Dr. Zypher Jude G. Regencia

**********

[NOTE: If reviewer comments were submitted as an attachment file, they will be attached to this email and accessible via the submission site. Please log into your account, locate the manuscript record, and check for the action link "View Attachments". If this link does not appear, there are no attachment files.]

To ensure your figures meet our technical requirements, please review our figure guidelines: https://journals.plos.org/plosone/s/figures

You may also use PLOS’s free figure tool, NAAS, to help you prepare publication quality figures: https://journals.plos.org/plosone/s/figures#loc-tools-for-figure-preparation.

NAAS will assess whether your figures meet our technical requirements by comparing each figure against our figure specifications.

Attachment Submitted filename: Plos ONE Review.docx

Attachment Submitted filename: renamed_f4e00.pdf

# Author comment: Author response to Decision Letter 1

**DOI:** 10.1371/journal.pone.0361200.r002

17 Mar 2026

Response to Reviewer Comments

We sincerely thank the reviewer for their thoughtful, detailed, and constructive feedback. Their comments have substantially strengthened the methodological transparency, accuracy, and interpretive balance of our manuscript. Below, we provide a point-by-point response to each comment, with all changes incorporated into the revised manuscript.

Abstract

Comment: The abstract would benefit from greater clarity and completeness. Please report the total number of participants included across all studies, in addition to the number of studies. In addition, the reported p-value is potentially confusing, as it appears to refer to the heterogeneity test rather than the effect estimate. I suggest either explicitly labeling it as the p-value for heterogeneity or removing it from the abstract altogether, as heterogeneity p-values are uncommon in abstracts and may be misinterpreted by readers.

Response: We thank the reviewer for this suggestion. The abstract now includes the total number of participants across all included studies (n = 25,690). The p-value for heterogeneity has been removed to avoid potential misinterpretation, as it referred to statistical heterogeneity rather than the effect estimate.

Additional changes as suggested by other reviewers:

1. Title: Added explicit designation of study design. Title now reads: "Association Between E Cigarette Use and Dyslipidemia: A Systematic Review and Meta Analysis of Observational Studies."

2. Abstract Methods: Clarified why subgroup analyses (age/sex) were not feasible and explicitly stated "due to insufficient stratified reporting in included studies.". Labeled as "unadjusted" weighted mean differences to prevent misinterpretation as causal/adjusted effects.

3. Abstract Results:

o All continuous lipid outcomes (TC, LDL, HDL, TG) now explicitly labeled as "unadjusted" weighted mean differences to prevent misinterpretation as causal/adjusted effects.

o Non significant TC finding softened to "non significant trend toward higher total cholesterol" to reflect the confidence interval crossing the null value.

4. Abstract Conclusion: Tempered causal language to match GRADE certainty ratings. Revised to " This synthesis suggests that vaping may be associated with dyslipidemia. However, the certainty of evidence is low to very low quality, precluding causal inferences. Further robust longitudinal studies are needed to explore these associations."

Introduction

Additional changes as suggested by other reviewers:

The Introduction has been revised as follows:

• Text has been added to explain the clinical relevance of dyslipidemia in this context, noting that sustained lipid perturbations can accelerate atherosclerotic cardiovascular disease (ASCVD) pathogenesis: "potentially translating into long-term cardiovascular risk even among younger cohorts."

• A definition of "exclusive vaping" has been inserted to clarify that the review minimizes confounding from dual use: "focus on 'exclusive vaping'—defined here as the use of e-cigarettes without concurrent use of combustible tobacco products."

Data Sources and Search Terms

Comment: Please write the search full string including Boolean operators, MeSH terms, truncation, and filters. Were they applied in all the databases. I recommend to show how the actual search done per database in the supplementary.

Response: We have added the complete search strategy for each database (PubMed, Cochrane CENTRAL, CINAHL, Embase, Scopus) as under the search strategy in the supplementary file. We included all MeSH/Emtree terms, free-text keywords, Boolean operators, truncation symbols, and applied filters.

Additional changes as suggested by other reviewers:

• We also included the search strings and the methods used to search Google Scholar, abiding by the best recommended practices in research methodology.

Screening of Studies

Comment: Screening of studies section appears to be redundant and confusing. Please clarify if a preliminary screening was done prior to full text screening. It is unclear and confusing because of the statement “Preliminarily included study records were retrieved in full text” and was followed by “Full texts of potentially eligible studies were then retrieved, and six reviewers, working in random three random pairs, independently assessed them.” Please clarify how many reviewers per stage of screening were involved (title and abstract screening / full text screening).

Comment: I believe that all studies excluded after full screening should have a reason for exclusion and I would suggest removing the term if applicable.

Comment: What is a random three random pairs mean? Please clarify.

Comment: Please detail the software used as well as include in the citation. For example, EndNote (Version 21, Clarivate Analytics).

Response: We appreciate this helpful clarification request. The Methods section has been revised to state explicitly that:

• Title/abstract screening was performed independently by six reviewers working in three pairs (two reviewers per record) using Rayyan (Rayyan.ai, 2023). Full-text assessment was also conducted independently by the same six reviewers in three pairs that are different from original three pairs matches as an attempt to minimize reviewer bias. Discrepancies at either stage were resolved by discussion or adjudication by a senior reviewer. This section was indeed redundant and was revised well to for better readability and clarity.

• The phrase "where applicable" regarding exclusion reasons has been removed; all exclusions after full-text screening did indeed include explicit reasons mentioned in the PRISMA diagram.

• EndNote (Version 21, Clarivate Analytics) is now cited as the reference management software.

Eligibility Criteria

Comment: Please include the type of observational studies i.e. longitudinal (cohort), case-control, cross-sectional studies) were considered as this would affect the results on association.

Comment: Please define what exclusive e-cigarette use. It may sound contradictory with the statement “who currently use e-cigarettes, regardless of their past vaping or smoking history.”

Comment: clarify that self-reported dyslipidemia was excluded. Suggest to add “Only” in the statement “ Biochemically validated measures of dyslipidemia were accepted.” If that is the purpose of the statement.

Comment: I noticed that the eligibility and outcomes sections do not specify the comparator group. Since the review aims to assess the association between exclusive e-cigarette use and lipid levels/dyslipidaemia, it is important to clarify what population the e-cigarette users were compared against (e.g., never-smokers, non-e-cigarette users, combustible cigarette smokers, or mixed controls). Without a clearly defined comparator, the direction and interpretation of the association may be ambiguous. Please specify the comparator criteria in the methods, and ensure consistency across eligibility criteria, data extraction, and statistical analyses. This clarification will strengthen the internal validity and reproducibility of the review.

Response: Thank you for highlighting these important points. We have revised the eligibility criteria to:

• Explicitly state inclusion of any observational study design (cross-sectional, case–control, and cohort studies).

• Define "exclusive e-cigarette users" as individuals reporting current e-cigarette use regardless of past smoking history, with no concurrent combustible tobacco use at the time of assessment.

• Clarify that only biochemically measured lipid levels were accepted; self-reported dyslipidemia diagnoses were excluded.

• Specify the comparator as "non-smokers" (individuals reporting no current use of e-cigarettes or combustible tobacco), as consistently defined across included studies.

Outcomes

Comment: Please explicitly state whether dyslipidaemia was defined by meeting any single lipid threshold or a combination of thresholds. Please mention source of your definition of dyslipidemia. In addition, present the primary (binary dyslipidaemia) and secondary (continuous lipid levels) outcomes more distinctly to avoid conceptual overlap. Ensure units and symbols are consistent (e.g., ≤, ≥) and specify whether thresholds were based on guideline sources. Overall, clearer wording will greatly improve interpretability.

Response: We have revised the Outcomes section to state that primary outcome was dyslipidemia, and defined as meeting any of the following thresholds based on the National Cholesterol Education Program Adult Treatment Panel III (NCEP ATP III) : TC ≥200 mg/dL (≥5.2 mmol/L), LDL C ≥130 mg/dL (≥3.4 mmol/L), HDL C <40 mg/dL (<1.0 mmol/L) in men or <50 mg/dL (<1.3 mmol/L) in women, and TG ≥150 mg/dL (≥1.7 mmol/L). Threshold sources are now cited in text. Secondary outcomes included a comparison of continuous lipid levels (mg/dL).

Data Extraction

Comment: I have a concern about the data extraction approach. From the description “Mean levels of lipids and their standards deviations were extracted from studies which did not report adjusted effect sizes”, it appears that mean lipid levels and standard deviations were only extracted from studies that did not provide adjusted effect estimates. If this interpretation is correct, it implies that studies reporting effect measures (e.g., adjusted mean differences, ORs, β-coefficients) may not have contributed raw lipid levels to the secondary outcome analyses. This raises two issues that need clarification:

• Were mean lipid levels and SDs extracted from studies that reported adjusted effect sizes whenever those data were available (either in the main text, tables, or supplementary files)? If not, this could lead to unnecessary exclusion of eligible data.

• Would excluding such data influence the validity of the secondary meta-analysis? Selectively extracting continuous data only from a subset of studies may bias the pooled mean estimates or reduce statistical power.

Comment: I recommend clarifying the extraction rule and, if applicable, justifying why studies that report effect measures could not contribute to the continuous outcome analysis, even when raw values were present. A sentence explaining how overlap between primary and secondary outcomes was handled would strengthen transparency.

Comment: It would be useful to specify whether a standardized and piloted data extraction form was used in Excel, and whether inter-extractor agreement was assessed before full extraction began.

Comment: I find the extraction section confusing and should be clarified. As written, “six authors independently extracted data in pairs” is contradictory, because extraction cannot be both independent and paired at the same time. Please specify whether each reviewer extracted data independently and was then paired for comparison, or whether each pair extracted jointly. It may be clearer to state: “data were extracted independently by pairs of reviewers, with discrepancies resolved through discussion or by a third reviewer.”

Comment: Please clarify whether extraction was done independently and in duplicate for all data fields (study characteristics, outcomes, demographics) or only for effect estimates.

Comment: I suggest to start of the with the study characteristics and followed by the outcomes.

Response: We thank the reviewer for identifying these ambiguities. The Data Extraction section has been comprehensively revised and reworked to state that:

• Mean lipid levels and SDs were extracted from all studies whenever available, regardless of whether adjusted effect estimates were reported, to enable secondary analyses. Studies contributed to both primary (adjusted effects) and secondary (raw means) analyses when both data types were present.

• Six reviewers worked in three pairs; within each pair, reviewers independently extracted all data fields (study characteristics, demographics, outcomes) using a standardized Excel form. Discrepancies were resolved by discussion or third-reviewer adjudication. This was similar to the distribution in the screening processes mentioned above.

• The narrative now presents study characteristics before outcomes for improved logical flow.

Quality Assessment and Certainty

Comment: It would help to briefly explain why the MASTER scale was selected rather than more commonly used tools for observational studies (e.g., Newcastle–Ottawa Scale or ROBINS-I). A short justification will assist readers who may be unfamiliar with MASTER.

Comment: The sentence structure suggests that seven domains were assessed, but the manuscript lists only six types of bias. Please align terminology and ensure all domains are clearly named.

Comment: Please clarify whether assessment was performed independently by multiple reviewers, how disagreements were resolved, and whether results were incorporated into the interpretation (e.g., sensitivity analysis, subgrouping by quality, or exclusion based on threshold scores).

Response: This section also needed a comprehensive revision to allow better readability and for a more clear and transparent approach. We have added a brief justification for selecting the MASTER scale: it provides a comprehensive, numerical assessment of methodological rigor across observational epidemiological studies, evaluating not only bias but also design, analysis, and interpretation quality, making it well-suited for this review. All seven MASTER domains are now explicitly named (Equal Recruitment, Retention, Ascertainment, Implementation, Prognosis, Sufficient Analysis, Temporal Precedence). We clarify that the same approach discussed in screening and data extraction was applied to quality assessment. Studies were divided into 3 thirds with and each third of studies was assessed independently by a pair of reviewers, with each reviewer assessing all studies then comparing with their pair. Disagreements were resolved by a senior reviewer. Quality scores were incorporated into the quality-effects model weighting but did not trigger study exclusion.

Data Synthesis

Comment: It is unclear why both the quality-effects and random-effects models were applied and which is considered the primary analysis—please clarify the rationale and interpretive hierarchy.

Comment: It is not entirely clear whether studies that reported unadjusted odds ratios were excluded from the pooled OR analysis. The methods imply that only adjusted ORs were extracted and synthesized; if so, this could lead to the exclusion of eligible effect sizes and potential bias. Please clarify whether unadjusted ORs were pooled when adjusted estimates were unavailable, and explain the rationale either way.

Comment: Please clarify the rationale for using several publication bias tools, specify which method provides the primary inference, and explain how discrepancies between methods were handled.

Response: Thank you so much for bringing our attention to the original Data synthesis section’s need for clarity. We have comprehensively revised this section and added the needed details for the section to state that:

• The quality-effects model was the primary analysis; the random-effects model served as a sensitivity analysis to assess robustness and enable comparison with other meta-analyses, as the random effects meta-analysis is the most commonly used method.

• A three-tier hierarchy guided effect estimate selection: (1) adjusted estimates prioritized; (2) unadjusted estimates pooled when adjusted data were unavailable; (3) raw means/SDs used only when neither adjusted nor unadjusted effects were reported.

• Leave-one-out sensitivity analyses were performed post-hoc for all outcomes; a study was deemed influential if its removal reduced I² by ≥20 percentage points or narrowed the 95% CI width by ≥30%.

• Publication bias was assessed using Doi plots (visual) and the LFK index (quantitative); the Doi plots provided the primary inference, with LFK index (>2 or <−2 indicating major asymmetry) as complementary assessment.

Results

Comment: To improve clarity and readability, I suggest adding a summary table in the main text that presents, for each lipid outcome, the pooled weighted mean difference (WMD) (with 95% CI), the pooled adjusted odds ratio where available, number of studies, total participants, heterogeneity (I²), model used (Quality adjusted/Random effects) and GRADE certainty. The detailed forest plots could then be moved to the Supplementary Materials.

Comment: “The seven included studies were published between 2019 and 2024, six were cross-sectional studies and one was a case-control study.” Please specify which studies were cross-sectional and which was case-control by citing the relevant references immediately after each study design.

Comment: “After meta-analysis, the overall weighted mean difference (WMD) showed a higher mean TC level in vapers, compared to non-vapers, of 3.66 mg/dL (95%CI -13.89 to 21.20) with a confidence interval still suggesting uncertainty.” - The interpretation of this result needs refinement. Although the point estimate suggests a higher mean TC level among vapers, the 95% CI crosses the null and is wide, indicating no statistically significant difference and substantial imprecision. The statement “with a confidence interval still suggesting uncertainty” is vague; please explicitly state that the result is not statistically significant and interpret it cautiously. Additionally, consider commenting on the clinical relevance of a 3.66 mg/dL difference, even if statistically significant, and ensure consistency between statistical inference and narrative conclusions.

Comment: “Given the small number of included studies (n = 5), estimates of heterogeneity (I²) and assessments of publication bias using Doi plots and the LFK index are likely unstable, and conclusions regarding unexplained heterogeneity or publication bias should therefore be interpreted with caution and more tentative language.

Comment: “Sensitivity analyses showed that the uncertainty significantly declined in the overall estimates, in leave-one out analyses upon leaving out Hasan, 2024 “ – The description of the sensitivity analysis requires clarification. Please specify the rationale for excluding Hasan (2024) in the leave-one-out analysis—was this study an outlier, at high risk of bias, methodologically different, or disproportionately influential? In addition, clarify the purpose of the sensitivity analysis (e.g., to assess robustness of pooled estimates, influence of individual studies, or impact of study quality). Without this justification, it is difficult to interpret the meaning of the reported reduction in uncertainty.

Comment: The term “strength of recommendation” is not appropriate in this context. GRADE is used here to assess the certainty (quality) of evidence, not the strength of a recommendation, which requires consideration of values, preferences, balance of benefits and harms, and resource use. Please revise the wording throughout to “certainty of evidence” (or “quality of evidence”) to accurately reflect the GRADE framework.

Comment: The statement on GRADE assessment needs clarification. Please specify the reasons for downgrading the certainty of evidence to very low (e.g., risk of bias, inconsistency, imprecision, indirectness, or publication bias), rather than only reporting the overall rating. Briefly summarizing the key domains driving the downgrading in the main text would improve transparency and align with GRADE reporting standards.

Comment: Figure 3 requires clearer outcome definition and terminology. The forest plot presents odds ratios, but it is unclear whether the outcome is low HDL, HDL dyslipidaemia (defined by a specific threshold), or another binary construct. Terms such as “high HDL” or “hyper-HDL” are not standard and should be avoided. Please specify the exact HDL cut-off used (e.g., HDL <1.0 mmol/L for men, <1.3 mmol/L for women), indicate the direction of effect (OR >1 reflects higher odds of low HDL), and revise the figure title and legend accordingly to ensure correct interpretation.

Comment: The handling of outlier studies and sensitivity analyses requires clarification. In the earlier analysis, Hasan (2024) was excluded in a leave-one-out sensitivity analysis, presumably because it was influential or an outlier; however, in the HDL analysis, Matheson appears to be an outlier but was not subjected to similar sensitivity testing. Please clarify the criteria used to identify influential or outlier studies and whether these criteria were applied consistently across outcomes. In addition, please state whether sensitivity analyses were pre-specified (a priori) in the protocol or conducted post-hoc in response to observed heterogeneity. Clear justification is necessary to avoid concerns about selective analysis.

Comment: “Since this study included observational studies, the GRADE certainty level starts as low and the GRADE was unchanged (low certainty) for the outcomes of TC, LDL and VLDL, but was upgraded to moderate in HDL and TG due to adjustment for estimated confounders. GRADE was then downgraded to low and very low due to high heterogeneity across the studies, possible risk of bias (low-quality cross-sectional studies), and uncertainty about publication bias.” - The description of the GRADE assessment is confusing and appears internally inconsistent. Earlier sections report very low certainty for HDL, yet this paragraph states that HDL (and TG) were upgraded to moderate certainty due to adjustment for confounders, before being downgraded again. Please reconcile these statements to ensure consistency across the manuscript. In addition, the rationale for upgrading is not sufficiently justified: while adjustment for confounders may support upgrading for residual confounding that would bias results toward the null, this is not appropriate when the underlying studies are at high risk of bias, particularly low-quality cross-sectional designs. GRADE upgrading criteria should be explicitly stated and applied cautiously; upgrading evidence that is already seriously limited by risk of bias is generally not recommended. I suggest clearly listing the specific upgrading and downgrading decisions for each outcome and revising the narrative to align with GRADE guidance.

Response: We have implemented all these improvements:

• Added Table 2 summarizing pooled estimates, participants, heterogeneity, models, and GRADE certainty for each lipid outcome.

• Specified and cited the six studies were cross-sectional (Lim 2024, Matheson 2024, Okafor 2022, Majid 2021, Kim 2020, Badea 2019) and one was case–control (Hasan 2024).

• Changes made to section 3.4 “Association between vaping and levels of TC”:

o Clarified statistical interpretation and added clinical context directly within the Results section:

� "The overall weighted mean difference (WMD) was 3.66 mg/dL higher in vapers compared to non-vapers (95% CI −13.89 to 21.20). However, the confidence interval crossed the null value (zero), indicating no statistically significant difference. Even if statistically significant, a mean difference of 3.66 mg/dL would likely be of minimal clinical relevance for cardiovascular risk assessment."

o Added methodological caveats regarding heterogeneity and bias metrics with sparse data:

� "However, with only five studies included, both the magnitude of heterogeneity and the bias assessment should be interpreted cautiously, and potential sources of heterogeneity could not be explored due to insufficient data for meaningful subgroup or meta-regression analyses."

o Explicitly justified the sensitivity analysis for Hasan 2024:

� "Leave-one-out sensitivity analyses showed that heterogeneity substantially decreased (I² reduced from 86.9% to 0%) and the confidence interval narrowed upon exclusion of Hasan 2024, the only case-control study, conducted exclusively in Iraqi men only, indicating disproportionate influence on the pooled estimate."

• Replaced all instances of "strength of recommendation" with "certainty of evidence" or “quality of evidence” throughout the manuscript.

• Revised Section 3.9 to transparently report GRADE ratings per outcome with explicit reasons for downgrading (e.g., TC and LDL downgraded to very low due to suspected publication bias; VLDL rated very low due to only two studies).

• Updated Figures 3 and 6 to label outcomes as "HDL dyslipidemia" and "TG dyslipidemia," with NCEP ATP III threshold definitions provided in figure footnotes.

Additional changes as suggested by other reviewers:

• To improve transparency regarding the adjusted analyses for HDL and TG, we have added the specific confounders adjusted for in each study directly into the Figure Legends. We have carefully revised the wording for LDL and VLDL findings to ensure clinical recommendations are cautious and reflect the limitations of unadjusted data.

Discussion

Comment: “This systematic review and meta-analysis revealed varying associations between e-cigarette use and serum lipid profiles. Overall, vaping was linked to higher total cholesterol (TC), LDL, VLDL, and triglyceride (TG) levels, and lower HDL levels, although the certainty of currently available evidence is very low. Adjusted odds ratios were only available for two lipids, and from only a few studies. For the rests of the lipids, only unadjusted mean differences were available for analysis.”- This paragraph appear overstated relative to the strength and consistency of the evidence. While the text states that vaping was “linked” to higher TC, LDL, VLDL, and TG and lower HDL levels, several of these associations—particularly TC—were not statistically significant, were based on very few studies (often n≈3–5), and relied largely on unadjusted mean differences. Only TG showed a statistically significant association based on adjusted effect estimates, whereas conclusions for other lipids are driven primarily by unadjusted analyses that are vulnerable to confounding. I recommend revising the language to clearly distinguish between adjusted and unadjusted findings, avoid causal or definitive phrasing (“linked”), and explicitly acknowledge that the observed mean differences are small, imprecise, and of uncertain clinical significance. These limitations should also be reflected consistently in the GRADE assessments and overall conclusions.

Response: We have revised the opening paragraph of the Discussion to accurately reflects statistical significance, adjustment status, and GRADE ratings. It now reads “This systematic review and meta-analysis revealed varying associations between e-cigarette use and serum lipid profiles. Overall, unadjusted analysis suggested higher mean levels of TC, LDL, VLDL, and triglycerides and lower HDL in vapers compared with non-vapers; total cholesterol showed a non-significant mean difference and the certainty of evidence was very low. Adjusted analyses, available only for HDL and triglycerides, indicated a stronger association with dyslipidemia patterns - specifically lower HDL and higher triglycerides - and the certainty of evidence was low.”.

Additional changes as suggested by other reviewers:

We have revised the text to strictly adhere to observational language, distinguish between mechanism and exposure, and explicitly address the "former smoker" and "health selection" phenomena as major interpretative factors. The following changes have been made:

• Causal Language: We have systematically replaced causal terms (e.g., "effect," "impact," "leads to") with observational terminology throughout the Discussion and Conclusion.

o Revised Text: "This systematic review and meta-analysis identified varying associations..." and "Adjusted analyses... indicated a stronger independent association..."

o Revised Text: "Though marketed as safer alternatives, observational evidence suggests e-cigarettes may negatively impact lipid metabolism..."

• Exposure Profiles vs. Mechanisms: We added a specific distinction between the shared mechanism (nicotine) and the differing exposure profiles (combustion products) to avoid over-reliance on the cigarette analogy.

o Inserted Text: "While e-cigarettes share the delivery of nicotine with these traditional products, it is important to note that the exposure profiles differ significantly; specifically, e-cigarettes lack the combustion-generated carbon monoxide and tar found in conventional cigarettes ..."

• Smoking History & Residual Confounding: We expanded the interpretation of the HDL and LDL findings to explicitly cite the lingering metabolic effects of past smoking as a primary alternative explanation, rather than relegating this solely to the limitations section.

o Inserted Text: "However, in interpreting these findings for e-cigarettes, the potential for residual confounding by past smoking history is substantial. Many e-cigarette users are former combustible cigarette smokers, and the observed lower HDL may partially reflect the lingering metabolic effects of past smoking rather than vaping alone ..."

• Reverse Causality / Health Selection: We incorporated the concept of "sick switchers" to address the potential for reverse causality.

o Inserted Text: "Furthermore, 'health selection' bias cannot be ruled out, wherein individuals with pre-existing health concerns or dyslipidemia switch from cigarettes to vaping to mitigate harm, thereby clustering higher-risk lipid profiles within the vaping group ..."

Conclusion

Comment: The conclusion should be revised to more accurately reflect the substantial limitations of the evidence and avoid overstating the findings. Phrases such as “vaping is likely associated with deranged lipid levels” imply a degree of certainty that is not supported by the data, given that most analyses were based on unadjusted observational studies, several associations were not statistically significant, heterogeneity was high, and overall GRADE certainty was low or very low. I recommend using more cautious, descriptive language (e.g., “limited and low-certainty evidence suggests possible differences in lipid levels, particularly triglycerides”) and explicitly noting the uncertainty and lack of causal inference. Aligning the conclusion more closely with the stated limitations will improve internal consistency and methodological rigor.

Response: The Conclusion has been revised to provide a transparent reflection of the study’s findings and their limitations without providing a false overstating of the conclusions. It now reads “This synthesis suggests possible differences in lipid levels among vapers compared with non-vapers. However, these findings are based on findings from a combination of adjusted and unadjusted analyses from observational studies. Several associations had substantial heterogeneity, and GRADE certainty ranged from very low to low. Consequently, the evidence does not support firm conclusions about an association - or its direction - between vaping and lipid profiles. Further robust longitudinal studies with adjustment for key confounders are needed to clarify these associations with greater certainty.”.

We are so grateful for the opportunity to improve our manuscript through this rigorous review. All changes have been implemented carefully, and we believe the revised version is substantially strengthened in transparency, accuracy, and interpretive balance of our manuscript. Thank you again for your valuable contribution to our work.

Response to Reviewer Comments

Dear Dr Regencia,

We sincerely thank you so much for your thoughtful, detailed, and constructive feedback. Your comments have substantially strengthened the methodological transparency, accuracy, and interpretive balance of our manuscript. Below we provide a point-by-point response to each comment, with all changes implemented in the revised manuscript.

Abstract

1) Overall / general impressions

Comment: This is a timely question and the manuscript is clearly structured (PRISMA, PROSPERO registration, GRADE). The narrative is generally easy to follow, and the authors are transparent that most evidence is low certainty and largely observational.

Comment: That said, the current evidence base is thin (7 studies; mostly cross-sectional), and the manuscript would benefit from tightening claims and strengthening how you handle confounding, “exclusive vaping” definitions, and the mixing of adjusted binary outcomes with unadjusted continuous outcomes. This is the biggest interpretability issue for readers.

Response: Thank you for your comment. We have revised the manuscript to now have softer language, better readability, and better flow of ideas.

2) Title / Abstract

Comment: Title: Clear and appropriate. Consider clarifying in the title (or subtitle) that the included evidence is observational (since causality cannot be inferred).

Comment: Abstract (Methods): You mention subgroup analyses (age/sex) but note they weren't possible. That's fine, but I'd recommend explicitly stating why (e.g., "insufficient stratified reporting") to pre-empt reviewer concerns.

Comment: Abstract (Results):

• The reporting mixes adjusted odds ratios (aORs) for dyslipidemia outcomes with weighted mean differences from unadjusted means. I’d strongly suggest explicitly labeling the WMD results as “unadjusted” in the abstract to avoid readers interpreting them as causal/adjusted effects.

• The TC result is reported as borderline (CI crosses 0). You might soften wording (“trend toward higher TC”) and keep emphasis proportional to certainty.

Comment: Abstract (Conclusion): The phrase "vaping is likely associated" is a bit strong given "very low–low" certainty; consider "may be associated" or "is associated in limited observational data."

Response: We appreciate these valuable suggestions to enhance precision and align language with the observational nature and low certainty of the evidence base. The Title and Abstract have been revised as follows:

• The title has been updated to explicitly state the observational design: "Association Between E Cigarette Use and Dyslipidemia: A Systematic Review and Meta Analysis of Observational Studies."

• The Abstract Methods section now explicitly states why subgroup analyses were not feasible: "Subgroup analyses by age and sex were planned a priori but could not be performed due to insufficient stratified reporting in the included studies."

• All continuous lipid outcomes in the Abstract Methods and Results are now explicitly labeled as unadjusted.

• The interpretation of the non significant TC finding has been softened to reflect the confidence interval crossing the null value: "showed a non significant trend toward higher total cholesterol"

• The Abstract Conclusion has been tempered to align with GRADE certainty ratings: "Current observational evidence suggests e cigarette use may be associated with dyslipidemia; however, the certainty of evidence is very low to low, precluding causal inference."

Additional changes as suggested by other reviewers:

• The abstract now includes the total number of participants across all included studies (n = 25,690).

• The p-value for heterogeneity has been removed to avoid potential misinterpretation, as it referred to statistical heterogeneity rather than the effect estimate.

3) Introduction

Comment: The rationale is strong: you connect ENDS uptake, cardiovascular risk, and dyslipidemia as a plausible mechanism.

Comment: Clarify why dyslipidemia matters clinically in this context—e.g., whether small shifts in LDL/HDL in young adults could translate into long-term ASCVD risk (even if speculative). Keep it balanced and not alarmist.

Comment: Pre-define what you mean by "exclusive vaping" in the introduction (because readers will immediately worry about former smoking/dual use).

Response: We appreciate the positive assessment of our rationale and the helpful suggestions to clarify clinical significance and definitions. The Introduction has been revised as follows:

• Text has been added to explain the clinical relevance of dyslipidemia in this context, noting that sustained lipid perturbations can accelerate atherosclerotic cardiovascular disease (ASCVD) pathogenesis: "potentially translating into long-term cardiovascular risk even among younger cohorts."

• A definition of "exclusive vaping" has been inserted to clarify that the review minimizes confounding from dual use: "focus on 'exclusive vaping'—defined here as the use of e-cigarettes without concurrent use of combustible tobacco products."

4) Methods

Comment: 4.1 Study design / protocol: PRISMA 2020 + PROSPERO registration are clear strengths.

• Briefly state any deviations from the registered protocol (if none, explicitly say "no deviations"). Reviewers often look for this.

Comment: 4.2 Search strategy: You searched major databases plus Google Scholar and updated to May 31, 2025—excellent coverage.

• Please report whether you applied any Google Scholar screening limits (e.g., first N pages/results) to ensure reproducibility.

• Consider adding the full search strings (not just "keywords") in the supplement if not already there.

Comment: 4.3 Eligibility criteria: You exclude dual users but allow participants "regardless of past vaping or smoking history." This opens a major confounding pathway: many "exclusive vapers" are former smokers, and prior smoking could drive lipid differences.

• Either restrict to never-smoker vapers (if data allow),

• or pre-specify a subgroup/sensitivity analysis comparing "exclusive vapers who never smoked" vs "exclusive vapers who are former smokers,"

• or at minimum clearly describe this as a key limitation and reflect it in GRADE downgrading decisions.

Comment: 4.4 Outcomes: The dyslipidemia cutoffs are provided—helpful.

• Specify whether you accepted each study's own dyslipidemia definitions if different, and how you harmonized them (or whether you did not). Outcome definition variability can create heterogeneity.

Comment: 4.5 Data extraction: The paired extraction process is reassuring.

• Add a short list of "core confounders" you attempted to extract (age, sex, BMI, smoking history, alcohol, diet, activity, socioeconomic status) to help readers assess residual confounding.

Comment: 4.6 Risk of bias / quality (MASTER): Using MASTER is interesting and defensible, but many readers are more familiar with NOS/ROBINS-I. Add 1–2 sentences justifying why MASTER was chosen and how to interpret its scores.

Comment: 4.7 Synthesis / statistics: You use a quality-effects (inverse heterogeneity) model and also random effects for comparison—that's thoughtful.

• Mixing adjusted ORs (binary outcomes) with unadjusted mean differences (continuous outcomes) creates an "apples vs oranges" feel. Consider reorganizing Results so it is crystal-clear which outcomes are adjusted vs unadjusted, and avoid implying they carry similar evidential weight.

• For very high heterogeneity (e.g., TG I² ~99%), consider whether pooling is meaningful; at minimum, add interpretive caution ("summary effect may not represent a common effect").

Response: We acknowledge that the methods section required some changes, and we appreciate your assistance to make this section comprehensive, detailed, organized, and transparent. We have carefully revised the methods section to state the following:

• Added a statement about deviation from PROSPERO protocol and mention explicitly that there were no major deviations from protocol. Subgroup analyses could not be conducted because of the insufficient reporting of stratified data in included studies but this was an additional or supplementary analysis in the protocol, not the main (overall) analysis as conducted in the study.

• We have added the complete search strategy for each database (PubMed, Cochrane CENTRAL, CINAHL, Embase, Scopus) as under the search strategy in supplementary file. We included all MeSH/Emtree terms, free-text keywords, Boolean operators, truncation symbols, and applied filters. We also included the search strings and the methods used to search google scholar, abiding by the best recommended practices in research methodology.

• We appreciate the reviewer highlighting the critical potential for confounding by past smoking history among exclusive vapers. The reported adjusted associations included prior tobacco smoking as a covariate to mitigate this specific bias brought by history of prior tobacco smoking. However, we have added a specific statement to the Limitations section acknowledging that, despite these statistical adjustments, residual confounding from past smoking history cannot be definitively ruled out: "residual confounding from prior combustible tobacco use remains a potential limitation.”

• We have reworked the outcomes section in the methods to specify that dyslipidemia was defined according to the The National Cholesterol Education Program (NCEP) Adult Treatment Panel III (ATP III) criteria. Studies reporting dyslipidemia mostly had this criteria as part of their definition for dyslipidemia, authors of included studies were also contacted to clarify the dyslipidemia definitions in their studies.

• The quality assessment section also needed a comprehensive revision to allow better readability and for a more clear and transparent approach. We have added a brief justification for selecting the MASTER scale: it provides a comprehensive, numerical assessment of methodological rigor across observational epidemiological studies, evaluating not only bias but also design, analysis, and interpretation quality, making it well-suited for this review. All seven MASTER domains are now explicitly named (Equal Recruitment, Retention, Ascertainment, Implementation, Prognosis, Sufficient Analysis, Temporal Precedence). We clarify that the same approach discussed in screening and data extraction was applied to quality assessment. Studies were divided into 3 thirds with and each third of studies was assessed independently by a pair of reviewers, with each reviewer assessing all studies then comparing with their pair. Disagreements were resolved by a senior reviewer. Quality scores were incorporated into the quality-effects model weighting but did not trigger study exclusion.

• We have now explicitly stated in the Results that all studies included in the odds ratio analysis reported adjusted estimates; no unadjusted ORs were used. We have clarified in the Methods that the analysis employed a Quality Effects model, which adjusts weights based on both the inverse variance and the quality of the evidence, thereby appropriately distinguishing the evidential weight of the findings. Regarding high heterogeneity (e.g., TG), we retained the pooled synthesis for transparency and to quantitatively assess whether a common effect exists, an insight not achievable through a purely descriptive approach. Furthermore, high heterogeneity likely comes from varying effect sizes in one direction, rather than evidence going in opposite direction. These outcomes are interpreted in text with appropriate caution to avoid overstating the results.

Additional changes as suggested by other reviewers:

• Screening of studies:

o Title/abstract screening was performed independently by six reviewers working in three pairs (two reviewers per record) using Rayyan (Rayyan.ai, 2023). Full-text assessment was also conducted independently by the same six reviewers in three pairs that are different from original three pairs matches as an attempt to minimize reviewer bias. Discrepancies at either stage were resolved by discussion or adjudication by a senior reviewer. This section was indeed redundant and was revised well to for better readability and clarity.

o The phrase "where applicable" regarding exclusion reasons has been removed; all exclusions after full-text screening did indeed include explicit reasons mentioned in the PRISMA diagram.

o EndNote (Version 21, Clarivate Analytics) is now cited as the reference management software.

• Eligibility Criteria

o Explicitly stated inclusion of any observational study design (cross-sectional, case–control, and cohort studies).

o Defined "exclusive e-cigarette users" as individuals reporting current e-cigarette use regardless of past smoking history, with no concurrent combustible tobacco use at the time of assessment.

o Specified the comparator as "non-smokers" (individuals reporting no current use of e-cigarettes or combustible tobacco), as consistently defined across included studies.

• Outcomes

o We have revised the Outcomes section to state that primary outcome was dyslipidemia, and defined as meeting any of the following thresholds based on the National Cholesterol Education Program Adult Treatment Panel III (NCEP ATP III) : TC ≥200 mg/dL (≥5.2 mmol/L), LDL C ≥130 mg/dL (≥3.4 mmol/L), HDL C <40 mg/dL (<1.0 mmol/L) in men or <50 mg/dL (<1.3 mmol/L) in women, and TG ≥150 mg/dL (≥1.7 mmol/L). Threshold sources are now cited in text. Secondary outcomes included a comparison of continuous lipid levels (mg/dL).

• Data Extraction: The Data Extraction section has been comprehensively revised and reworked to state that:

o Mean lipid levels and SDs were extracted from all studies whenever available, regardless of whether adjusted effect estimates were reported, to enable secondary analyses. Studies contributed to both primary (adjusted effects) and secondary (raw means) analyses when both data types were present.

o Six reviewers worked in three pairs; within each pair, reviewers independently extracted all data fields (study characteristics, demographics, outcomes) using a standardized Excel form. Discrepancies were resolved by discussion or third-reviewer adjudication. This was similar to the distribution in the screening processes mentioned above.

o The narrative now presents study characteristics before outcomes for improved logical flow.

• Data Synthesis: We have comprehensively revised this section and added needed details for the section to state that:

o The quality-effects model was the primary analysis; the random-effects model served as a sensitivity analysis to assess robustness and enable comparison with conventional meta-analyses.

o A three-tier hierarchy guided effect estimate selection: (1) adjusted estimates prioritized; (2) unadjusted estimates pooled when adjusted data unavailable; (3) raw means/SDs used only when neither adjusted nor unadjusted effects were reported.

o Leave-one-out sensitivity analyses were performed post-hoc for all outcomes; a study was deemed influential if its removal reduced I² by ≥20 percentage points or narrowed the 95% CI width by ≥30%.

o Publication bias was assessed using Doi plots (visual) and the LFK index (quantitative); the LFK index (>2 or <−2 indicating major asymmetry) provided the primary inference, with Doi plots as complementary visual assessment.

5) Results

Comment: 5.1 Study selection / PRISMA: Flow is clearly reported (4,160 identified; 7 included).

• Include the full exclusion table (with reasons per full-text) in the supplement if not already provided.

Comment: 5.2 Study characteristics (Table 1): The table is helpful but would benefit from additional columns for:

(a) how "exclusive vaping" was defined (self-report? timeframe?);

(b) smoking history (never/former/unknown); and

(c) lipid measurement method (fasting vs non-fasting; lab vs self-report).

These features are likely drivers of heterogeneity and bias.

Comment: 5.3 Lipid-specific findings

• (TC): High heterogeneity and publication bias signals are present; the leave-one-out sensitivity analysis suggests one study drives uncertainty. Highlight which clinical or design features of that study differ (e.g., population, fasting status, control definition).

• ( HDL & TG): The adjusted analyses (aORs) are the most convincing pieces—worth emphasizing—but also clarify which confounders were adjusted for in each study (in text or supplement).

• (LDL/VLDL): Results appear more consistent but remain largely unadjusted. Maintain cautious wording and avoid clinical recommendations based solely on unadjusted differences.

Comment: 5.4 GRADE: Evidence certainty ranging from very low to low is appropriately justified (downgrades for heterogeneity, bias, publication bias concerns). Consider presenting a compact "Summary of Findings" table in the main text (with detailed tables in supplement) as this is often the single most useful output for readers.

Response: We thank the reviewer for this detailed assessment of our findings. We have revised the Results and Discussion to address these points:

• The reasons for exclusion of full text screening are summarized in the PRISMA diagram under the “reports excluded” section.

• We appreciate the reviewer's suggestion to include these critical study features. We agree that these details are important for interpreting heterogeneity. However, adding these columns directly to Table 1 would compromise readability due to space constraints. Instead, we have moved the detailed lipid measurement methods to Supplementary Studies methods section. The definition of "exclusive vaping" was applied strictly according to the criteria outlined in the Methods section for all included studies. Regarding smoking history, we noted that reporting was very limited across the primary studies.

• We have now explicitly justified the sensitivity analysis for Hasan 2024: "Leave-one-out sensitivity analyses showed that heterogeneity substantially decreased (I² reduced from 86.9% to 0%) and the confidence interval narrowed upon exclusion of Hasan 2024, the only case-control study, conducted exclusively in Iraqi men with an imbalanced design (100 vapers vs. 50 controls), indicating disproportionate influence on the pooled estimate."

• To improve transparency regarding the adjusted analyses for HDL and TG, we have added the specific confounders adjusted for in each study directly into the Figure Legends. We have carefully revised the wording for LDL and VLDL findings to ensure clinical recommendations are cautious and reflect the limitations of unadjusted data.

• Added Table 2 summarizing pooled estimates, participants, heterogeneity, models, and GRADE certainty for each lipid outcome.

Additional changes as suggested by other reviewers:

• Specified and cited the six studies were cross-sectional (Lim 2024, Matheson 2024, Okafor 2022, Majid 2021, Kim 2020, Badea 2019) and one was case–control (Hasan 2024).

• Replaced all instances of "strength of recommendation" with "certainty of evidence" or “quality of evidence” throughout the manuscript.

• Revised Section 3.9 to transparently report GRADE ratings per outcome with explicit reasons for downgrading (e.g., TC and LDL downgraded to very low due to suspected publication bias; VLDL rated very low due to only two studies).

• Updated Figures 3 and 6 to label outcomes as "HDL dyslipidemia" and "TG dyslipidemia," with NCEP ATP III threshold definitions provided in figure footnotes.

• Additional changes made to section 3.4 “Association between vaping and levels of TC”:

o Clarified statistical interpretation and added clinical context directly within the Results section:

� "The overall weighted mean difference (WMD) was 3.66 mg/dL higher in vapers compared to non-vapers (95% CI −13.89 to 21.20). However, the confidence interval crossed the null value (zero), indicating no statistically significant difference. Even if statistically significant, a mean difference of 3.66 mg/dL would likely be of minimal clinical relevance for cardiovascular risk assessment."

o Added methodological caveats regarding heterogeneity and bias metrics with sparse data:

� "However, with only five studies included, both the magnitude of heterogeneity and the bias assessment should be interpreted cautiously, and potential sources of heterogeneity could not be explored due to insufficient data for meaningful subgroup or meta-regression analyses."

6) Discussion

Comment: Strengths section is fair and transparent, including plausible biological mechanisms (nicotine → catecholamines → lipolysis → FFAs → TG/VLDL).

Comment: Tighten causal language throughout: cross-sectional designs and residual confounding warrant "association" rather than "effect" terminology.

Comment: Expand discussion of smoking-history confounding: former smoking is likely the biggest alternative explanation for observed associations and deserves prominence in interpretation (not only in limitations).

Comment: Consider discussing reverse causality/health selection bias: individuals with dyslipidemia or health concerns may switch from cigarettes to vaping (or vice versa), which can bias observed associations.

Comment: When comparing to cigarette or waterpipe studies, explicitly acknowledge that while mechanisms may overlap (nicotine/inflammation), exposure profiles differ substantially (device types, aerosol constituents, puffing topography). This will make the discussion more nuanced and less dependent on cigarette analogies.

Response: We are grateful for these insightful comments, which have significantly improved the nuance and scientific rigor of our Discussion. We have revised the text to strictly adhere to observational language, distinguish between mechanism and exposure, and explicitly address the "former smoker" and "health selection" phenomena as major interpretative factors. The following changes have been made:

• Causal Language: We have systematically replaced causal terms (e.g., "effect," "impact," "leads to") with observational terminology throughout the Discussion and Conclusion.

o Revised Text: "This systematic review and meta-analysis identified varying associations..." and "Adjusted analyses... indicated a stronger independent association..."

o Revised Text: "Though marketed as safer alternatives, observational evidence suggests e-cigarettes may negatively impact lipid metabolism..."

• Exposure Profiles vs. Mechanisms: We added a specific distinction between the shared mechanism (nicotine) and the differing exposure profiles (combustion products) to avoid over-reliance on the cigarette analogy.

o Inserted Text: "While e-cigarettes share the delivery of nicotine with these traditional products, it is important to note that the exposure profiles differ significantly; specifically, e-cigarettes lack the combustion-generated carbon monoxide and tar found in conventional cigarettes ..."

• Smoking History & Residual Confounding: We expanded the interpretation of the HDL and LDL findings to explicitly cite the lingering metabolic effects of past smoking as a primary alternative explanation, rather than relegating this solely to the limitations section.

o Inserted Text: "However, in interpreting these findings for e-cigarettes, the potential for residual confounding by past smoking history is substantial. Many e-cigarette users are former combustible cigarette smokers, and the observed lower HDL may partially reflect the lingering metabolic effects of past smoking rather than vaping alone ..."

• Reverse Causality / Health Selection: We incorporated the concept of "sick switchers" to address the potential for reverse causality.

o Inserted Text: "Furthermore, 'health selection' bias cannot be ruled out, wherein individuals with pre-existing health concerns or dyslipidemia switch from cigarettes to vaping to mitigate harm, thereby clustering higher-risk lipid profiles within the vaping group ..."

Additional changes as suggested by other reviewers:

We have revised the opening paragraph of the Discussion to accurately reflects statistical significance, adjustment status, and GRADE ratings. It now reads “This systematic review and meta-analysis revealed varying associations between e-cigarette use and serum lipid profiles. Overall, unadjusted analysis suggested higher mean levels of TC, LDL, VLDL, and triglycerides and lower HDL in vapers compared with non-vapers; total cholesterol showed a non-significant mean difference and the certainty of evidence was very low. Adjusted analyses, available only for HDL and triglycerides, indicated a stronger association with dyslipidemia patterns - specifically lower HDL and higher triglycerides - and the certainty of evidence was low.”.

7) Conclusion

Comment: The conclusion aligns with findings but should make the "low certainty" message even more prominent, as this is what readers and journalists will most likely quote.

Comment: Consider adding a final sentence specifying what "robust longitudinal studies" should measure: device type, nicotine concentration, duration/intensity of use, fasting lipid panels, validated smoking history (never/former/never smoker), and ideally repeated measures to assess temporal relationships.

Response: The Conclusion has been revised to provide a transparent reflection of the study’s findings and their limitations without providing a false overstating of the conclusions. It now reads “This synthesis suggests possible differences in lipid levels among vapers compared with non-vapers. However, these findings are based on findings from a combination of adjusted and unadjusted analyses from observational studies. Several associations had substantial heterogeneity, and GRADE certainty ranged from very low to low. Consequently, the evidence does not support firm conclusions about an association - or its direction - between vaping and lipid profiles. Further robust longitudinal studies with adjustment for key confounders are needed to clarify these associations with greater certainty.”

We are grateful for the opportunity to improve our manuscript through this rigorous review. All changes have been implemented carefully, and we believe the revised version is substantially strengthened in transparency, accuracy, and interpretive balance of our manuscript. Thank you again for your valuable contribution to our work.

Attachment Submitted filename: Final Response To Reviewers 25 Feb.docx

# Peer review: Decision Letter 1

**DOI:** 10.1371/journal.pone.0361200.r003

**Authors:** Zypher Jude Regencia

20 Apr 2026

Dear Dr. Chivese,

Thank you for submitting your manuscript to PLOS ONE. After careful consideration, we feel that it has merit but does not fully meet PLOS ONE’s publication criteria as it currently stands. Therefore, we invite you to submit a revised version of the manuscript that addresses the points raised during the review process. As there were few reviewers available, I served as the Academic Editor and Reviewer 2.

Please submit your revised manuscript by Jun 04 2026 11:59PM. If you will need more time than this to complete your revisions, please reply to this message or contact the journal office at plosone@plos.org. When you're ready to submit your revision, log on to https://www.editorialmanager.com/pone/ and select the 'Submissions Needing Revision' folder to locate your manuscript file.

- A letter that responds to each point raised by the academic editor and reviewer(s). You should upload this letter as a separate file labeled 'Response to Reviewers'.
- A marked-up copy of your manuscript that highlights changes made to the original version. You should upload this as a separate file labeled 'Revised Manuscript with Track Changes'.
- An unmarked version of your revised paper without tracked changes. You should upload this as a separate file labeled 'Manuscript'.

If you would like to make changes to your financial disclosure, please include your updated statement in your cover letter. Guidelines for resubmitting your figure files are available below the reviewer comments at the end of this letter.

If applicable, we recommend that you deposit your laboratory protocols in protocols.io to enhance the reproducibility of your results. Protocols.io assigns your protocol its own identifier (DOI) so that it can be cited independently in the future. For instructions see: https://journals.plos.org/plosone/s/submission-guidelines#loc-laboratory-protocols. Additionally, PLOS ONE offers an option for publishing peer-reviewed Lab Protocol articles, which describe protocols hosted on protocols.io. Read more information on sharing protocols at https://plos.org/protocols?utm_medium=editorial-email&utm_source=authorletters&utm_campaign=protocols.

As the corresponding author, your ORCID iD is verified in the submission system and will appear in the published article. PLOS supports the use of ORCID, and we encourage all coauthors to register for an ORCID iD and use it as well. Please encourage your coauthors to verify their ORCID iD within the submission system before final acceptance, as unverified ORCID iDs will not appear in the published article. *Only*  the individual author can complete the verification step; PLOS staff *cannot*  verify ORCID iDs on behalf of authors.

We look forward to receiving your revised manuscript.

Kind regards,

Zypher Jude G. Regencia, Ph.D.

Academic Editor

PLOS One

Journal Requirements:

If the reviewer comments include a recommendation to cite specific previously published works, please review and evaluate these publications to determine whether they are relevant and should be cited. There is no requirement to cite these works unless the editor has indicated otherwise.

[Note: HTML markup is below. Please do not edit.]

Reviewers' comments:

Reviewer's Responses to Questions

**Comments to the Author**

Reviewer #1: All comments have been addressed

Reviewer #2: (No Response)

**********

2. Is the manuscript technically sound, and do the data support the conclusions?

Reviewer #1: Yes

Reviewer #2: Partly

**********

3. Has the statistical analysis been performed appropriately and rigorously?

Reviewer #1: Yes

Reviewer #2: Yes

**********

4. Have the authors made all data underlying the findings in their manuscript fully available?

The PLOS Data policy

Reviewer #1: Yes

Reviewer #2: No

**********

5. Is the manuscript presented in an intelligible fashion and written in standard English?

Reviewer #1: Yes

Reviewer #2: Yes

**********

Reviewer #1: The review explored an important and timely topic. The authors have adequately addressed the issues.

Reviewer #2: Dear Authors,

Thank you for the revised manuscript and accompanying response letter. The manuscript is certainly better than the original version. I am thankful for the revisions to temper the language, clarify the observational design, better define exclusive vaping and the comparison group, separate primary and secondary outcomes, and clarify the results and conclusion. Overall, these changes help the manuscript, and it is now better, fairer, and easier to read.

However, I do not think it is entirely responsive yet. I have a few concerns that were not fully addressed, or not addressed in a fully consistent way in the response letter and the manuscript. First, the distinction between adjusted binary lipid outcomes and unadjusted continuous lipid outcomes is now much clearer in the manuscript, and I appreciate the abstract, discussion, and conclusion now clearly state this as an issue. However, this is still the major interpretability issue with the paper, and I think it should be kept front and center throughout the manuscript. I appreciate the improvements to the discussion, where you now clearly state that only adjusted data were available for HDL and triglycerides, while the rest of the lipid analyses were mostly unadjusted. That is a major improvement. However, I would encourage one more pass to ensure every sentence in the results and discussion is equally clear about this issue, particularly when discussing the LDL and VLDL outcomes.

Second, I do not think the data extraction section fully addresses the original comment. You now clarify that 6 reviewers working in 3 pairs independently extracted all data fields using a standard Excel form. However, the original comment also asked if the Excel form was pilot-tested and if inter-extractor agreement was assessed before proceeding with full extraction. That part of the original comment appears to have gone unanswered. Please either fill this in in the manuscript (if this was done) or clearly state that this was not formally assessed. As it stands, I feel the response is incomplete.

Third, there appears to be some inconsistency regarding the publication bias analysis that should be rectified. In the response letter, you state that the Doi plots provided the primary inference and the LFK index was supplementary. However, in the text itself, the LFK index is stated to provide the primary inference and the Doi plots were used as a supplementary visual analysis. The two statements cannot both be true. Please pick one and apply it consistently in the manuscript and the response letter, and clarify how discrepancies between the visual and quantitative analyses were managed.

Fourth, some of the responses to big picture conceptual points are too vague. For example, one of the major comments highlighted the issues of confounding, the definition of exclusive vaping, and the problem of interpreting the mixture of adjusted and unadjusted outcomes. The response to that broad concern was essentially that the language is softer and the flow is better. While the flow was indeed improved in some of the later sections, the response would be better if it pointed to the exact changes made in the methods, results, and discussion. This would make the response letter more convincing and easier for editors to evaluate.

Fifth, I appreciate the conclusion is better aligned with the low certainty evidence, and I appreciate you now avoid the stronger causal language. However, I think one of the earlier suggestions is only partially fulfilled. You were asked to clarify what future robust longitudinal studies should actually measure, such as device type, nicotine concentration, duration and intensity of use, fasting lipid level assessment, validated smoking status, and repeated measurements over time. You now mention adjustment for key confounders in the conclusion, but do not provide the specific list of suggestions as requested. I think you could strengthen the conclusion by incorporating one additional sentence with concrete suggestions for future study designs.

Overall, I think the paper has improved a great deal, and many of the major concerns were addressed meaningfully. My suggestion is for a targeted revision, not a full redo. The bulk of the remaining work is not to reframe the paper, but to tighten up a few incomplete responses, resolve some internal inconsistencies, and ensure the response letter and manuscript match perfectly. Once that is done, the paper will be in a much stronger place.

**********

what does this mean?). If published, this will include your full peer review and any attached files.

If you choose “no”, your identity will remain anonymous but your review may still be made public.

**Do you want your identity to be public for this peer review?**  For information about this choice, including consent withdrawal, please see our Privacy Policy

Reviewer #1: No

Reviewer #2: **Yes:**  Zypher Jude G. Regencia, Ph.D.

# Author comment: Author response to Decision Letter 2

**DOI:** 10.1371/journal.pone.0361200.r004

26 Jun 2026

Response to Reviewer Comments

Manuscript title: The Association Between E-cigarette Use and Dyslipidemia: A Systematic Review and Meta-analysis of Observational Studies

Dear Editors and Reviewers,

We sincerely thank you for your continued engagement with our manuscript and for the detailed, constructive feedback provided in this second round of review. We are grateful that you found the revised manuscript substantially improved in clarity, balance, and readability. Your remaining comments helped us identify areas where our earlier responses were incomplete or inconsistent, and we have now addressed each point with targeted revisions. Below, we provide a point-by-point response.

Reviewer Comment 1

Thank you for the revised manuscript and accompanying response letter. The manuscript is certainly better than the original version. I am thankful for the revisions to temper the language, clarify the observational design, better define exclusive vaping and the comparison group, separate primary and secondary outcomes, and clarify the results and conclusion. Overall, these changes help the manuscript, and it is now better, fairer, and easier to read.

Response:

We appreciate your generous feedback. We are pleased that the revisions improved the manuscript’s clarity, balance, and readability. Your thoughtful comments throughout the review process have strengthened the work substantially, and we are grateful for the time and expertise you have invested.

Reviewer Comment 2

However, I do not think it is entirely responsive yet. I have a few concerns that were not fully addressed, or not addressed in a fully consistent way in the response letter and the manuscript. First, the distinction between adjusted binary lipid outcomes and unadjusted continuous lipid outcomes is now much clearer in the manuscript, and I appreciate that the abstract, discussion, and conclusion now clearly state this as an issue. However, this is still the major interpretability issue with the paper, and I think it should be kept front and center throughout the manuscript. I appreciate the improvements to the discussion, where you now clearly state that only adjusted data were available for HDL and triglycerides, while the rest of the lipid analyses were mostly unadjusted. That is a major improvement. However, I would encourage one more pass to ensure every sentence in the results and discussion is equally clear about this issue, particularly when discussing the LDL and VLDL outcomes.

Response:

Thank you for this important comment. We agree that the distinction between adjusted binary outcomes and unadjusted continuous lipid outcomes is central to interpreting this meta-analysis. We therefore made another pass through the Abstract, Results, Discussion, and Conclusion to ensure that this distinction remains explicit throughout the manuscript.

Specifically, we revised the Abstract to distinguish adjusted odds ratios for dyslipidemia from unadjusted weighted mean differences for continuous lipid levels. We also revised each lipid-specific Results section to state whether adjusted or unadjusted estimates were available:

• Section 3.4, TC: none of the studies reported adjusted effect sizes for the association between vaping and total cholesterol; the analysis was based on unadjusted mean differences.

• Section 3.5, HDL: adjusted odds ratios were available from two studies, while continuous HDL analyses were based on unadjusted mean HDL levels.

• Section 3.6, LDL: no adjusted effect sizes were available; the LDL meta-analysis was based on unadjusted mean differences.

• Section 3.7, VLDL: neither study reported adjusted effect sizes; the VLDL meta-analysis was based on two studies reporting unadjusted means.

• Section 3.8, TG: adjusted odds ratios were available from two studies, while the continuous TG analysis was based on unadjusted mean TG levels.

We also revised the Discussion to keep this issue central to interpretation. The LDL paragraph now states, in part:

“For LDL, our analysis was based entirely on unadjusted mean differences and showed higher LDL levels among e-cigarette users compared to non-users, with a WMD of 12.40 mg/dL and moderate heterogeneity. Our results correspond with those reported in observational studies exploring cigarette smoking and LDL. For instance, an observational study showed a measurable difference in lipid parameters between cigarette smokers and non-smokers, indicating markedly higher levels of LDL cholesterol in smokers compared to non-smokers (53). The observed LDL increases are likely linked to nicotine-induced lipolysis and FFA release (19, 43). Because these estimates were not adjusted for smoking history or other cardiovascular risk factors, they should be interpreted cautiously.”

The VLDL paragraph now states, in part:

“In relation to VLDL, two studies reporting unadjusted means showed higher VLDL levels among e-cigarette users than non-users, with a pooled uWMD of +3.23mg/dL. Our findings align with limited but growing evidence that suggests vaping may influence VLDL levels. One meta-analysis reported that young smokers aged between 8-19 showed substantially higher serum VLDL cholesterol levels compared to non-smokers. This increase was also associated with increased LDL cholesterol and triglycerides, along with reduced HDL cholesterol, suggesting that cigarette smoking may lead to early lipid profile disturbances (53). Because only two studies contributed to this analysis and neither provided adjusted estimates, the certainty of evidence remains very low.”

Reviewer Comment 3

Second, I do not think the data extraction section fully addresses the original comment. You now clarify that 6 reviewers working in 3 pairs independently extracted all data fields using a standard Excel form. However, the original comment also asked if the Excel form was pilot-tested and if inter-extractor agreement was assessed before proceeding with full extraction. That part of the original comment appears to have gone unanswered. Please either fill this in in the manuscript, if this was done, or clearly state that this was not formally assessed. As it stands, I feel the response is incomplete.

Response:

We appreciate the reviewer’s request for greater methodological transparency. We revised the Data Extraction section, Section 2.6, to explicitly address both pilot-testing and inter-extractor agreement.

The Excel extraction form was not formally pilot-tested on a subset of studies. Because only seven studies met the inclusion criteria, we developed a standardized form to capture all prespecified study, participant, exposure, comparator, and lipid-outcome variables and applied it uniformly to all included studies.

We also clarified that formal inter-rater reliability or inter-extractor agreement statistics were not calculated before full extraction. Six authors worked in three independent pairs, and within each pair, reviewers extracted all data fields independently. Discrepancies were resolved through discussion or adjudication by a third reviewer.

The revised manuscript text now states:

“Data were extracted using a standardized Microsoft Excel spreadsheet. The extraction form was not formally pilot-tested on a subset of studies because only seven studies met the inclusion criteria; instead, the form was developed to capture all prespecified study, participant, exposure, comparator, and lipid-outcome variables and was applied uniformly to all included studies. Six authors (HR, SM, AH, HA, WA, JN) worked in three pairs, and within each pair, reviewers extracted all data fields independently. Formal inter-rater reliability or inter-extractor agreement statistics were not calculated before full extraction. Discrepancies were resolved through discussion or adjudication by a third reviewer. Extracted data included study characteristics, participant demographics, exposure definitions, comparator definitions, covariates, and lipid outcome data.”

We hope this fully addresses the concern.

Reviewer Comment 4

Third, there appears to be some inconsistency regarding the publication bias analysis that should be rectified. In the response letter, you state that the Doi plots provided the primary inference and the LFK index was supplementary. However, in the text itself, the LFK index is stated to provide the primary inference and the Doi plots were used as a supplementary visual analysis. The two statements cannot both be true. Please pick one and apply it consistently in the manuscript and the response letter, and clarify how discrepancies between the visual and quantitative analyses were managed.

Response:

Thank you for identifying this inconsistency. We apologize for the confusion in our previous response letter. Our intended approach was to use the LFK index as the primary quantitative criterion for assessing asymmetry, with Doi plots used as complementary visual tools.

We have now aligned the response letter and manuscript accordingly. The Abstract Methods section now states that publication bias was assessed using Doi plots and the LFK index, with the LFK index used as the primary quantitative criterion. The Methods section further clarifies how discrepancies between the visual and quantitative assessments were managed.

The revised Methods text states:

“Publication bias was assessed using complementary visual and quantitative methods: (1) visual inspection of Doi plots, and (2) quantitative assessment via the LFK index (asymmetry >2 or <-2 interpreted as major bias) (32). The LFK index served as the primary basis for inference regarding bias presence; when visual and quantitative assessments diverged, the quantitative LFK result was prioritized to minimize subjectivity. Findings from both tools are reported to enhance transparency.”

Reviewer Comment 5

Fourth, some of the responses to big picture conceptual points are too vague. For example, one of the major comments highlighted the issues of confounding, the definition of exclusive vaping, and the problem of interpreting the mixture of adjusted and unadjusted outcomes. The response to that broad concern was essentially that the language is softer and the flow is better. While the flow was indeed improved in some of the later sections, the response would be better if it pointed to the exact changes made in the methods, results, and discussion. This would make the response letter more convincing and easier for editors to evaluate.

Response:

We agree and have revised this response to map each conceptual concern to specific manuscript changes.

First, regarding confounding by combustible tobacco smoking, we revised the Introduction to clarify that the review focused on exclusive vaping to reduce confounding from concurrent combustible tobacco use, while acknowledging that past combustible tobacco use may still confound observed lipid associations. The revised Introduction states:

“To reduce confounding from concurrent combustible tobacco use, this review focused on “exclusive vaping,” defined here as e-cigarette use without concurrent use of combustible tobacco products. However, past combustible tobacco use may still confound observed lipid associations and was therefore considered in the interpretation of the findings.”

Second, regarding the comparator group, we revised Section 2.4 to avoid overgeneralizing the comparator definition. The revised Eligibility Criteria state:

“Studies were eligible if they compared exclusive e-cigarette users, defined as current e-cigarette users without concurrent tobacco smoking, regardless of their past smoking history, with non-current e-cigarette users or non-smokers.”

Third, regarding adjusted and unadjusted outcomes, we revised the Abstract, Methods, Results, Discussion, and Conclusion to distinguish adjusted odds ratios for binary dyslipidemia outcomes from unadjusted weighted mean differences for continuous lipid outcomes. In the Methods, we describe the hierarchy of effect estimates, prioritizing adjusted estimates when available and clearly stating when unadjusted estimates or raw means were used. The revised Data Synthesis section states:

“For each lipid parameter (TC, HDL, LDL, VLDL, and TG), we conducted meta-analyses using a hierarchical approach to effect estimates: adjusted effect measures (aOR, and their 95% CIs) were prioritized when available; if adjusted estimates were absent, unadjusted effect measures were extracted and pooled; when neither adjusted nor unadjusted effect estimates were reported, raw continuous data (mean lipid levels and standard deviations) were used to compute weighted mean differences (WMDs). This hierarchy ensured maximal inclusion of eligible effect sizes while prioritizing estimates least susceptible to confounding.”

Fourth, we expanded the Discussion and Limitations to emphasize residual confounding and possible health-selection bias. The Limitations section now states:

“A major limitation is residual confounding by combustible tobacco history. Although the review focused on exclusive current e-cigarette use and excluded dual use where possible, many exclusive e-cigarette users may be former smokers. Lipid abnormalities caused by previous combustible tobacco exposure may persist after switching to e-cigarettes, making it difficult to isolate the metabolic effect of vaping itself. Reverse causality is also possible, because individuals with cardiovascular risk factors may switch from cigarettes to e-cigarettes as a perceived harm-reduction strategy.”

Reviewer Comment 6

Fifth, I appreciate the conclusion is better aligned with the low certainty evidence, and I appreciate you now avoid the stronger causal language. However, I think one of the earlier suggestions is only partially fulfilled. You were asked to clarify what future robust longitudinal studies should actually measure, such as device type, nicotine concentration, duration and intensity of use, fasting lipid level assessment, validated smoking status, and repeated measurements over time. You now mention adjustment for key confounders in the conclusion, but do not provide the specific list of suggestions as requested. I think you could strengthen the conclusion by incorporating one additional sentence with concrete suggestions for future study designs.

Response:

Thank you for noting this. We agree that the conclusion should provide more concrete guidance for future studies. We revised both the Abstract Conclusion and the main manuscript Conclusion to specify the key design elements that future longitudinal studies should include.

The revised Abstract Conclusion now calls for well-designed longitudinal studies using validated smoking-status assessment, detailed vaping exposure measures, fasting lipid panels, repeated lipid measurements, and adjustment for key cardiometabolic confounders.

The revised main Conclusion now has this added statement:

“Further well-designed longitudinal studies should prospectively collect detailed vaping exposure data, including device type, nicotine concentration, and duration and intensity of use; use validated assessments of smoking status to distinguish never smokers, former smokers, current smokers, and dual users; obtain fasting lipid panels; and adjust for key cardiometabolic and behavioral confounders to better assess temporality and clarify these associations.”

Reviewer Comment 7

Overall, I think the paper has improved a great deal, and many of the major concerns were addressed meaningfully. My suggestion is for a targeted revision, not a full redo. The bulk of the remaining work is not to reframe the paper, but to tighten up a few incomplete responses, resolve some internal inconsistencies, and ensure the response letter and manuscript match perfectly. Once that is done, the paper will be in a much stronger place.

Response:

We thank the reviewer for this constructive summary and agree that the remaining issues required targeted correction rather than a full reframing of the manuscript. We have carefully reviewed the manuscript and response letter together, corrected the remaining internal inconsistencies, clarified the methodological points identified above, and aligned the response letter with the revised manuscript. We believe these targeted revisions have strengthened the manuscript and improved the transparency and interpretability of the findings.

Sincerely,

Tawanda Chivese

Attachment Submitted filename: Response to Reviewer Comments June 18.docx

# Editor report: Decision Letter 2

**DOI:** 10.1371/journal.pone.0361200.r005

**Authors:** Zypher Jude Regencia

29 Jul 2026

Dear Dr. Chivese,

Please submit your revised manuscript by Sep 12 2026 11:59PM. If you will need more time than this to complete your revisions, please reply to this message or contact the journal office at plosone@plos.org. When you're ready to submit your revision, log on to https://www.editorialmanager.com/pone/ and select the 'Submissions Needing Revision' folder to locate your manuscript file.

- A letter that responds to each point raised by the academic editor and reviewer(s). You should upload this letter as a separate file labeled 'Response to Reviewers'.
- A marked-up copy of your manuscript that highlights changes made to the original version. You should upload this as a separate file labeled 'Revised Manuscript with Track Changes'.
- An unmarked version of your revised paper without tracked changes. You should upload this as a separate file labeled 'Manuscript'.

If you would like to make changes to your financial disclosure, please include your updated statement in your cover letter. Guidelines for resubmitting your figure files are available below the reviewer comments at the end of this letter.

If applicable, we recommend that you deposit your laboratory protocols in protocols.io to enhance the reproducibility of your results. Protocols.io assigns your protocol its own identifier (DOI) so that it can be cited independently in the future. For instructions see: https://journals.plos.org/plosone/s/submission-guidelines#loc-laboratory-protocols. Additionally, PLOS ONE offers an option for publishing peer-reviewed Lab Protocol articles, which describe protocols hosted on protocols.io. Read more information on sharing protocols at https://plos.org/protocols?utm_medium=editorial-email&utm_source=authorletters&utm_campaign=protocols.

As the corresponding author, your ORCID iD is verified in the submission system and will appear in the published article. PLOS supports the use of ORCID, and we encourage all coauthors to register for an ORCID iD and use it as well. Please encourage your coauthors to verify their ORCID iD within the submission system before final acceptance, as unverified ORCID iDs will not appear in the published article. *Only*  the individual author can complete the verification step; PLOS staff *cannot*  verify ORCID iDs on behalf of authors.

We look forward to receiving your revised manuscript.

Kind regards,

Zypher Jude G. Regencia, Ph.D.

Academic Editor

PLOS One

Journal Requirements:

1. If the reviewer comments include a recommendation to cite specific previously published works, please review and evaluate these publications to determine whether they are relevant and should be cited. There is no requirement to cite these works unless the editor has indicated otherwise.

2. Please review your reference list to ensure that it is complete and correct. If you have cited papers that have been retracted, please include the rationale for doing so in the manuscript text, or remove these references and replace them with relevant current references. Any changes to the reference list should be mentioned in the rebuttal letter that accompanies your revised manuscript. If you need to cite a retracted article, indicate the article’s retracted status in the References list and also include a citation and full reference for the retraction notice.

Additional Editor Comments:

Reviewers' comments:

The authors have substantially addressed the previous comments; however, several minor revisions remain necessary before the manuscript can be accepted. Please ensure that the response letter accurately reflects the changes made in the manuscript, particularly regarding the Abstract and Introduction. The Abstract should explicitly identify the LFK index as the primary quantitative criterion for assessing publication bias, consistent with the Methods, and the corresponding LFK values should be reported consistently for all outcomes where available. The Introduction should clearly acknowledge that previous combustible tobacco exposure may confound the observed associations, while the comparator definition should be harmonized across the manuscript and response letter. In the Discussion, please revise the statement that “the majority” of the LDL findings were unadjusted, as all LDL estimates were unadjusted, and clarify that neither VLDL study provided adjusted estimates and that the certainty of evidence was very low. Please also consolidate the repetitive recommendations in the Conclusion and correct the remaining duplicated, incomplete, or grammatically incorrect sentences, spacing problems, and formatting inconsistencies throughout the manuscript.

[NOTE: If reviewer comments were submitted as an attachment file, they will be attached to this email and accessible via the submission site. Please log into your account, locate the manuscript record, and check for the action link "View Attachments". If this link does not appear, there are no attachment files.]

To ensure your figures meet our technical requirements, please review our figure guidelines: https://journals.plos.org/plosone/s/figures

You may also use PLOS’s free figure tool, NAAS, to help you prepare publication quality figures: https://journals.plos.org/plosone/s/figures#loc-tools-for-figure-preparation.

NAAS will assess whether your figures meet our technical requirements by comparing each figure against our figure specifications.

# Author comment: Author response to Decision Letter 3

**DOI:** 10.1371/journal.pone.0361200.r006

8 Sep 2026

Response to reviewers - [PONE-D-25-42648R2]

Sept 7, 2026

Dear Editor, many thanks to the reviewers for their comments. Please find before the response to the reviewers’ comments

1. The authors have substantially addressed the previous comments; however, several minor revisions remain necessary before the manuscript can be accepted. Please ensure that the response letter accurately reflects the changes made in the manuscript, particularly regarding the Abstract and Introduction.

Response – we have done this

2. The Abstract should explicitly identify the LFK index as the primary quantitative criterion for assessing publication bias, consistent with the Methods, and the corresponding LFK values should be reported consistently for all outcomes where available.

Response – this has now been implemented

3. The Introduction should clearly acknowledge that previous combustible tobacco exposure may confound the observed associations, while the comparator definition should be harmonized across the manuscript and response letter.

Response – we have implemented this

4. In the Discussion, please revise the statement that “the majority” of the LDL findings were unadjusted, as all LDL estimates were unadjusted, and clarify that neither VLDL study provided adjusted estimates and that the certainty of evidence was very low.

Response – we have implemented this suggestion.

5. Please also consolidate the repetitive recommendations in the Conclusion and correct the remaining duplicated, incomplete, or grammatically incorrect sentences, spacing problems, and formatting inconsistencies throughout the manuscript.

Response – we have implemented this now.

Attachment Submitted filename: Response to reviewers Sept 7, 2026.docx

# Editor report: Decision Letter 3

**DOI:** 10.1371/journal.pone.0361200.r007

**Authors:** Zypher Jude Regencia

8 Oct 2026

The Association Between E-cigarette Use and Dyslipidemia: A Systematic Review and Meta-analysis of Observational Studies

PONE-D-25-42648R3

Dear Dr. Chievese,

We’re pleased to inform you that your manuscript has been judged scientifically suitable for publication and will be formally accepted for publication once it meets all outstanding technical requirements.

Within one week, you’ll receive an e-mail detailing the required amendments. When these have been addressed, you’ll receive a formal acceptance letter and your manuscript will be scheduled for publication.

An invoice will be generated when your article is formally accepted. Please note, if your institution has a publishing partnership with PLOS and your article meets the relevant criteria, all or part of your publication costs will be covered. Please make sure your user information is up-to-date by logging into Editorial Manager at Editorial Manager® and clicking the ‘Update My Information' link at the top of the page. For questions related to billing, please contact billing support.

If your institution or institutions have a press office, please notify them about your upcoming paper to help maximize its impact. If they’ll be preparing press materials, please inform our press team as soon as possible -- no later than 48 hours after receiving the formal acceptance. Your manuscript will remain under strict press embargo until 2 pm Eastern Time on the date of publication. For more information, please contact onepress@plos.org.

Kind regards,

Zypher Jude G. Regencia, Ph.D.

Academic Editor

PLOS One

Additional Editor Comments (optional):

Reviewers' comments:

# Editor report: Acceptance letter

**DOI:** 10.1371/journal.pone.0361200.r008

**Authors:** Zypher Jude Regencia

PONE-D-25-42648R3

PLOS One

Dear Dr. Chivese,

I'm pleased to inform you that your manuscript has been deemed suitable for publication in PLOS One. Congratulations! Your manuscript is now being handed over to our production team.

At this stage, our production department will prepare your paper for publication. This includes ensuring the following:

* All references, tables, and figures are properly cited

* All relevant supporting information is included in the manuscript submission,

* There are no issues that prevent the paper from being properly typeset

You will receive further instructions from the production team, including instructions on how to review your proof when it is ready. Please keep in mind that we are working through a large volume of accepted articles, so please give us a few days to review your paper and let you know the next and final steps.

Lastly, if your institution or institutions have a press office, please let them know about your upcoming paper now to help maximize its impact. If they'll be preparing press materials, please inform our press team within the next 48 hours. Your manuscript will remain under strict press embargo until 2 pm Eastern Time on the date of publication. For more information, please contact onepress@plos.org.

You will receive an invoice from PLOS for your publication fee after your manuscript has reached the completed accept phase. If you receive an email requesting payment before acceptance or for any other service, this may be a phishing scheme. Learn how to identify phishing emails and protect your accounts at https://explore.plos.org/phishing.

If we can help with anything else, please email us at customercare@plos.org.

Thank you for submitting your work to PLOS One and supporting open access.

Kind regards,

PLOS One Editorial Office Staff

on behalf of

Dr. Zypher Jude G. Regencia

Academic Editor

PLOS One
